# Pentaborate(1-) Salts and a Tetraborate(2-) Salt Derived from C_2_- or C_3_-Linked Bis(alkylammonium) Dications: Synthesis, Characterization, and Structural (XRD) Studies

**DOI:** 10.3390/molecules25010053

**Published:** 2019-12-23

**Authors:** Michael A. Beckett, Bashdar I. Meena, Thomas A. Rixon, Simon J. Coles, Peter N. Horton

**Affiliations:** 1 School of Natural Sciences, Bangor University, Bangor LL57 2UW, UK; chs030@bangor.ac.uk (B.I.M.); chp81d@bangor.ac.uk (T.A.R.); 2 Chemistry Department, University of Southampton, Southampton SO17 1BJ, UK; S.J.Coles@soton.ac.uk (S.J.C.); P.N.Horton@soton.ac.uk (P.N.H.)

**Keywords:** BET analysis, borate, diaminoalkane salts, oxidipolyborate, pentaborate(1-), tetraborate(2-), TGA, XRD

## Abstract

The synthesis of a number of pentaborate(1-) salts from cations arising from *N*-substituted α,α-, α,β-, and α,γ-diaminoalkanes has been attempted in aqueous solution from B(OH)_3_ and the appropriate diammine in a 10:1 ratio. Despite relatively mild work-up conditions the pentaborate(1-) salts prepared were not always as anticipated and the following compounds were isolated in good yield: [Me_2_NH(CH_2_)_2_NHMe_2_][B_5_O_6_(OH)_4_]_2_ (**1**), [Et_2_NH(CH_2_)_2_NHEt_2_][B_5_O_6_(OH)_4_]_2_ (**2**), [Et_2_NH_2_][B_5_O_6_(OH)_4_] (**3**), [Me_2_NH_2_][B_5_O_6_(OH)_4_] (**4**), [Me_2_NH(CH_2_)_3_NHMe_2_][B_5_O_6_(OH)_4_]_2_ (**5**), [Et_2_NH(CH_2_)_3_NHEt_2_][B_5_O_6_(OH)_4_]_2_ (**6**), [Me_3_NCH_2_CH=CH_2_][B_5_O_6_(OH)_4_] (**7**), and [Me_3_N(CH_2_)_3_NMe_3_] [B_5_O_6_(OH)_4_]_2_^.^0.5H_2_O (**8**). The tetraborate(2-) salt, [Me_3_N(CH_2_)_2_NMe_3_][B_4_O_5_(OH)_4_]^.^2B(OH)_3_^.^2H_2_O (**9**) was obtained in moderate yield (41%) from a 3:1 reaction of B(OH)_3_ with [Me_3_N(CH_2_)_2_NMe_3_](OH)_2_. All compounds were characterized by spectroscopy (^1^H, ^11^B, ^13^C NMR and IR) and thermal gravimetric analysis (TGA). BET analysis on materials derived thermally from selected samples (**1**, **2**, **6**, **7**) all had porosities of < 1 m^2^/g, demonstrating that they were non-porous. Single-crystal XRD structures were obtained for **2**, **3**, **7**, **8** and **9** and all contain extensive H-bonded polyborate lattices.

## 1. Introduction

**Figure 1:**
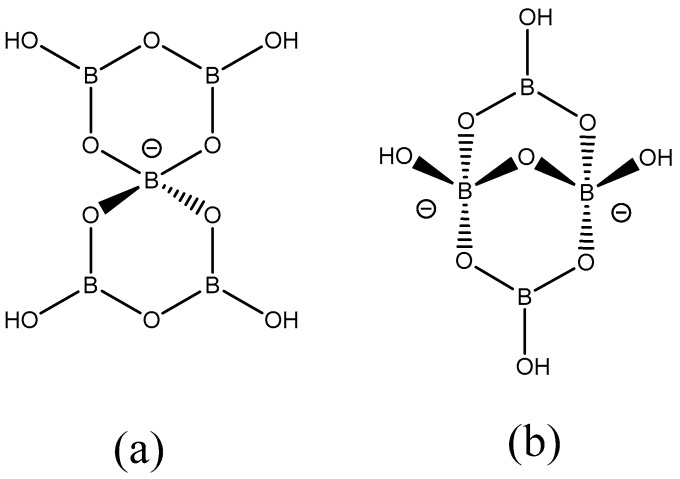
Schematic drawings of (**a**) pentaborate(1-) anion observed in **1**–**8**, and (**b**) tetraborate(2-) anion observed in **9**.

Salts and neutral species containing polyoxidoborate (polyborate) anions occur as minerals and many synthetic derivatives have now also been prepared [1,2,3,4,5]. These compounds are structurally diverse, with the polyborate anions generally comprised of trigonal planar 3-coordinate and tetrahedral 4-coordinate boron centres, linked via oxygen bridges with as many terminal OH or O^−^ groups as is necessary to complete the required geometry at boron [6,7,8,9,10,11]. Some polyborate salts are sold as bulk chemicals for use in the glass/vitreous, fire retardant, and agricultural industries [12,13,14], whilst other synthetic polyborates have interesting and specialized physical e.g., semiconducting, luminescent, non-linear optical (NLO) properties [5,15]. We are interested in synthetic polyborate chemistry and recently have started to investigate non-metal cation stabilized silicate/borate solutions as potential bio-stimulants for agricultural use [12,16,17]. Since choline-like cations and tetraalkylammonium cations are known to structure-direct silicate species towards cubic octasilicate [Si_8_O_12+n_(OH)_8-n_]^n-^ anions [18,19] our attention has been directed towards *N*-substituted aminoalkane and *N*-substituted α,ω-diaminoalkane cations. This has led us to investigate, as part of a broader parallel study on synthetic polyborate chemistry, the interaction in aqueous solution between B(OH)_3_ and cations derived from *N*-substituted α,ω-diaminoalkanes and the results of this investigation are presented in this manuscript. In particular we report herein the synthesis and characterization of seven new polyborate salts together with single-crystal XRD studies on five of these compounds. Schematic drawings of the pentaborate(1-) and tetraborate(2-) anions present in these salts are given in Figure 1. Aspects of solid-state H-bond interactions, important in stabilizing the solid-state structures, are also discussed.

## 2. Results and Discussion

### 2.1. Synthesis

**Scheme 1:**
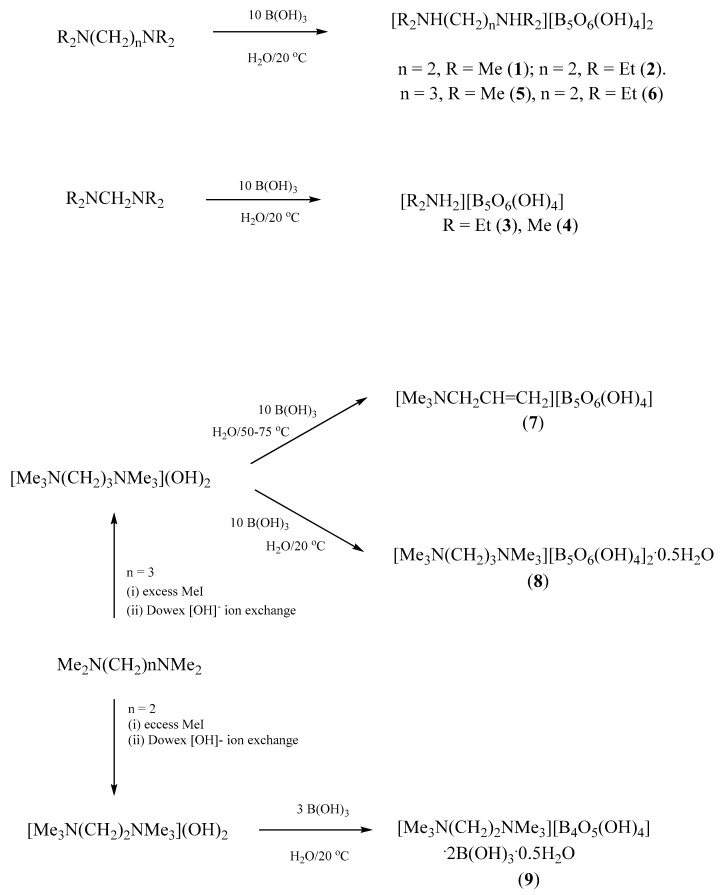
Reactions detailing the synthesis of **1**–**9**.

Synthetic strategies to prepare pentaborate(1-) salts partnered with organic cations include the reaction of a free base amine with B(OH)_3_ in aqueous solution or use of OH^-^ activated ion-exchange resins with quaternary ammonium halide salts [5]. Schubert and co-workers [19] have successfully applied the former method to α,ω-diaminoalkanes and have isolated a number of pentaborate(1-) salts containing a +2 diammonium cations of composition [H_3_N(CH_2_)_x_NH_3_][B_5_O_6_(OH)_4_]_2_ (x = 5, 6, 8-12). In this study, the pentaborate(1-) salts [Me_2_NH(CH_2_)_2_NHMe_2_][B_5_O_6_(OH)_4_]_2_ (**1**), [Et_2_NH(CH_2_)_2_NHEt_2_][B_5_O_6_(OH)_4_]_2_ (**2**), [Et_2_NH_2_][B_5_O_6_(OH)_4_] (**3**), [Me_2_NH_2_][B_5_O_6_(OH)_4_] (**4**), [Me_2_NH(CH_2_)_3_NHMe_2_][B_5_O_6_(OH)_4_]_2_ (**5**), and [Et_2_NH(CH_2_)_3_NHEt_2_][B_5_O_6_(OH)_4_]_2_ (**6**) were readily prepared as crystalline solids from aqueous solution in yields of 87–99% by the reaction of 10 equivalents of B(OH)_3_ with the diaminoalkane in aqueous solution at room temperature. Compounds **2**, **3**, **5** and **6** are previously unreported. Compound **1** has recently been prepared serendipitously in low yield (~10%) as a by-product from the reaction of a fluorinated borane CF_3_(CF_2_)_5_CH_2_(OLi)CH_2_PPh_2_^.^BH_3_ with [PtCl_2_(COD)] in the presence of Me_2_N(CH_2_)_2_NMe_2_ [20]. Likewise, **4** has been previously obtained in very low yield (a few crystals) as a by-product of a reaction of B(OH)_3_ with B_2_(NMe_2_)_4_ followed by crystallization from water [21]. *N*,*N*,*N*’*N*’-Tetraakyldiaminomethane cations are less stable than the tetrasubstituted diaminoethane or diaminopropane cations in aqueous solution and the formation of the corresponding dialkylammonium pentaborate(1-) salts **3** and **4** in high yields is not unexpected and further demonstrates the ready cleavage of the C-N aminal bonds [22,23,24,25]. Further characterization data for **1** and **4** are reported within this manuscript. Scheme 1 contains all the reactions studied in this work. 

The pentaborate(1-) salt [Me_3_NCH_2_CH=CH_2_][B_5_O_6_(OH)_4_] (**7**) was obtained as the isolated product from crystallization of the crude solid sample obtained from the reaction of [Me_3_NCH_2_CH_2_CH_2_NMe_3_](OH)_2_ (from by exchange from [Me_3_N(CH_2_)_3_NMe_3_]I_2_) with 10B(OH)_3_ followed by removal of the aqueous solvent on a hot water bath and oven drying (65 °C). This product rather than the expected product, [Me_3_N(CH_2_)_3_NMe_3_][B_5_O_6_(OH)_4_]_2_ was obtained since the [Me_3_N(CH_2_)_3_NMe_3_]^2+^ cation initially present rapidly undergoes a Hofmann elimination [26,27], with elimination of NMe_3_, under the workup conditions. Repeating the reaction but maintaining the reaction mixture at room temperature during the workup resulted in the high yield (96%) synthesis of expected product [Me_3_N(CH_2_)_3_NMe_3_][B_5_O_6_(OH)_4_]_2_ which after recrystallization at room temperature from aqueous solution by slow evaporation gave the compound as its hemihydrate, [Me_3_N(CH_2_)_3_NMe_3_][B_5_O_6_(OH)_4_]_2_^.^0.5H_2_O (**8**), as crystals suitable for single-crystal XRD studies.

The pentaborate(1-) salt, [Me_3_N(CH_2_)_2_NMe_3_][B_5_O_6_(OH)_4_]_2_, has been previously reported by us from the ion exchange method from [Me_3_N(CH_2_)_2_NMe_3_]I_2_ and B(OH)_3_ in good (80%) yield [28]. We now report that the related salt, [Me_3_N(CH_2_)_2_NMe_3_][B_4_O_5_(OH)_4_]^.^2B(OH)_3_^.^2H_2_O (**9**), could be obtained in moderate yield (41%) by crystallization of an aqueous solution containing a [Me_3_N(CH_2_)_2_NMe_3_](OH)_2_/B(OH)_3_ in a ratio of 1:3. Reactions of the other amines or [Me_3_N(CH_2_)_3_NMe_3_](OH)_2_ with B(OH)_3_ at this 1:3 ratio were not explored within this study. Compound **9** contains a tetraborate(2-) rather than a pentaborate(1-) anion, observed for **1**–**8**, and this anion is drawn schematically in Figure 1(b). Spectroscopic (IR and NMR) and thermal analysis data, including porosity measurements on materials obtained thermally, for these compounds **1**–**9** are described in Section 2.2 and XRD studies on **2**, **3**, **7**, **8** and **9** are described in Section 2.3.

### 2.2. Thermal Analysis, Porosity Measurements and Spectroscopic Data

Compounds **1**–**9** gave satisfactory C, H, N (combustion) elemental analysis data and thermal gravimetric analysis (TGA) data consistent with their formulations. Non-metal cation polyborates have been previously reported to decompose thermally (in air) to afford glassy B_2_O_3_ residues [5,19,29]. This thermal decomposition is usually a two-step processes with the lower temperature step associated with dehydration and cross-linking of hydroxyl groups of the polyborate anions to give condensed polyborate salts. The higher temperature step involves oxidation of the cations to afford the glassy residue. Thus for example, **1**, dehydrates to a condensed polyborate, [Me_2_NH(CH_2_)_2_NHMe_2_][B_10_O_16_] in the temperature range 150–300 °C with loss of 4H_2_O then with oxidation of organic cation to leave residual 5B_2_O_3_ at 300–650 °C. Compound **9**, which is formulated as a tetraborate(2-) salt with 2 interstitial B(OH)_3_ and 2 interstitial H_2_O molecules afforded 3B_2_O_3_ by a similar two-step process. Selected samples of **1**–**6** were either heated to 250 °C for 1h (**1**, **2**, **5**, **6**) or 600 °C for 1 h (**1**–**3**, **6**) to generate samples approximating to the ′condensed′ borate salt or the ′pyrolysed′ glassy solid that could be used for porosity measurements by use of the Brunauer-Emmett-Teller (BET) method [30]. Measured porosities of the thermally treated samples were in the range 0.06–0.94 m^2^/g indicating that they were all essentially non-porous and in accord with previous work [31,32].

^1^H-, ^13^C- and ^11^B-NMR spectra were recorded on compounds **1**–**9** (D_2_O solvent) and ^1^H- and ^13^C- spectra were in accord with the cations present. Exchangeable NH and B-OH protons were absent as specific signals and it is assumed were observed in the HOD signal present at +4.79 ppm as has been observed previously in related non-metal cation systems [31,32,33]. The pentaborate(1-) anion, although very stable in the solid-state and is readily templated by many organic cations [34], ′decomposes′ in aqueous solution as various equilibrium determined borate species are rapidly obtained and influenced by the boron concentration, the temperature and the pH of the aqueous solution [35,36]. However, ^11^B spectra of **1**–**8** were obtained and these samples all showed three peaks centred at 1 (~5) 13 (~35%) and 18 (~60%) ppm in a pattern typical of solutions arising from pentaborate(1-) salts [31,32,33,34,37]. The ^11^B spectra of **9**, originating from a tetraborate(2-) salt, was significantly different with three peaks now at 1.3 (5%), 7.4 (43%) and 11.7 (52%).The upfield shift of the ‘average’ ^11^B chemical shift on going from a pentaborate (1-) anion (**1–8**) to a tetraborate(2-) anion (**9**) is in accord with the average B/charge ratio changing from 5:1 to 2:1 [34].

IR spectra were obtained on all compounds and strong B-O stretches in the fingerprint region 1450–650 cm^−1^ are readily observed [38]. A band assigned to an asymmetric B-O stretch associated with the tetrahedral boron centre at ~925 cm^−1^ and usually diagnostic of a pentaborate(1-) anion [30,39] was clearly evident in samples **1**–**8**. There are many fewer reports of IR spectra of tetraborate(2-) species and some have been tabulated [38] but diagnostic bands have not been reported. It should be noted that the tetraborate(2-) salt **9** also displayed a stretch at 927 cm^−1^ in the region considered diagnostic for a pentaborate(1-) salt.

### 2.3. Single-Crystal XRD Studies

Compounds **2**, **3**, **7**, **8** and **9** have been investigated by single-crystal X-ray diffraction studies and pertinent crystallographic data are given in the experimental section, with full details available in the Supplementary Material. These studies confirm their formulation based on spectroscopic, thermal and analytical data described in Section 2.2. All structures were ionic and comprised of the expected organic cations for **2**, **3**, **8** and **9**, or the cation for **7** arising through the Hofmann elimination, with associated polyborate anions. 

**Figure 2:**
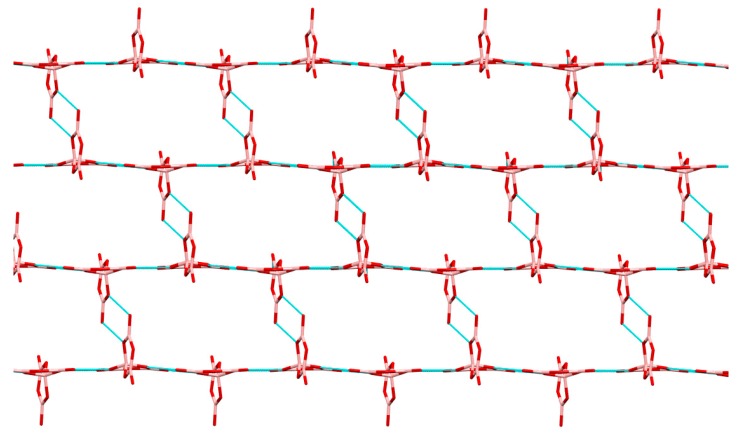
Giant lattice arising from pentaborate(1-)-pentaborate(1-) interactions in **3**, viewed along the *b* axis. Cations have been omitted for clarity, but are situated within ′cavities′. Within this ′plane′ each pentaborate(1-) in shown H-bonding to 3 other pentaborate(1-) anions by R_2_^2^(8) interactions. A fourth C(8) interaction (not shown) links these planes.

**Figure 3:**
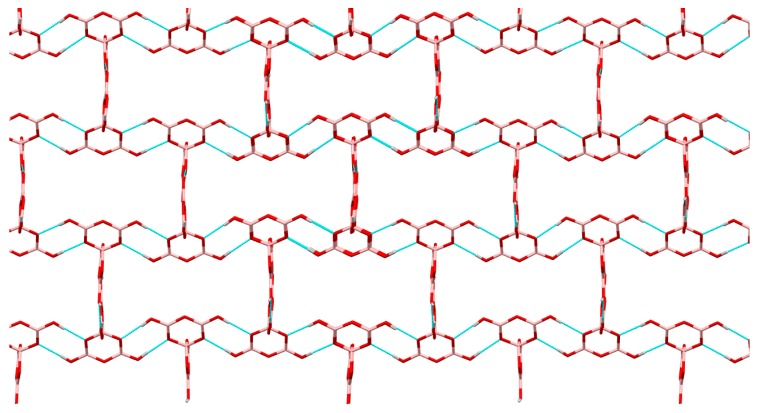
Giant lattice arising from pentaborate(1-)-pentaborate(1-) interactions in **2**, viewed along the *a* axis. Cations have been omitted for clarity, but are situated within ′cavities′. Within this ′plane′ each pentaborate(1-) in shown H-bonding to 3 other pentaborate(1-) anions by R_2_^2^(8) interactions. The fourth R_2_^2^(12) interaction is not visible from this view.

**Figure 4:**
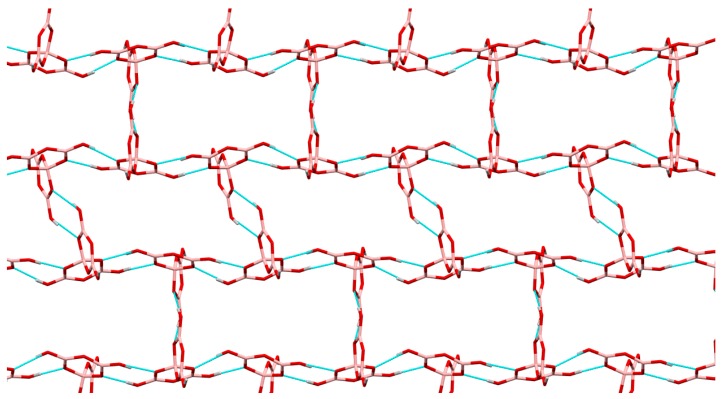
Giant lattice arising from pentaborate(1-)-pentaborate(1-) interactions in **8**, viewed along the *c* axis. Cations have been omitted for clarity, but are situated within rectangular ′cavities′. Within this ′plane′ each pentaborate(1-) in shown H-bonding to 3 other pentaborate(1-) anions by R_2_^2^(8) interactions. The C(8) Interaction (not shown) links these planes.

Compounds **2**, **3**, **7**, and **8** all contain the insular pentaborate(1-) anion, [B_5_O_6_(OH)_4_]^−^. Compound **8** is co-crystallized with 0.5 molecules of H_2_O. There is some disorder present in the cation and/or co-crystallized molecules all pentaborate(1-) anion structures except **3**. Bond lengths and bond angles found for C, N atoms within the organic cations [19,28,37,40,41] are as expected as are the B-O bond lengths, OBO and BOB angles observed for the [B_5_O_6_(OH)_4_]^−^ anion [19,21,28,29,31,32,33,34,35]; for full details see the Supplementary Information. H-bonding interactions within polyborate salts are extremely common and anion-anion interactions between pentaborate(1-) units, forming giant lattices, are well documented and are signaled as a strong driving force [5,29,34] in templating the crystallization of these anions from the Dynamic Combinatorial Library [42,43,44,45] arising from B(OH)_3_ under basic conditions [35,36]. The pentaborate (1-) anions in **2**, **3**, **7**, and **8** all form multiple anion-anion interactions and **2** and **3** have additional cation-anion NH^…^O H-bond interactions. The acceptor sites for the four anion-anion H-bond interactions from each independent pentaborate(1-) anion can be codified [19,37] as α,β,α,α and α,α,α,β (two independent anions) for **2**, α,α,α,γ for **3**, α,β,α,α for **7**, and α,β,α,α and α,α,β,α (two independent anions) for **8**. Using Etter nomenclature [46] the H-bond interactions per pentaborate are 3 R_2_^2^(8) and 1 R_2_^2^(12) for **2**, and 3 R_2_^2^(8) and 1 C(8) for **3**, **7**, and **8**. Compound **7** adopts the familiar ′herringbone′ structure [29,31] whereas **2**, **3** and **8** are unique. The structure of **3** (Figure 2) is similar to the ′stepped brickwall′ structure observed in [4-MepyH, 4-Mepy][B_5_O_6_(OH)_4_] [37]; however the structures are different since **3** has a C(8) interaction involving γ acceptor sites rather than β sites. Views of the anion giant structures in **2** and **8** are given in Figure 3 and Figure 4 respectively.

The structural characterization of **9** is considered in more detail in this manuscript since it contains a tetraborate(2-) anion and there are far fewer crystallographic reports in the literature featuring this anion; the anion is found partnered with 2Na^+^ in the well-known borax [47,48] but is less commonly found with non-metal cation [40,49,50,51,52] or transition metal cation salts [53,54,55,56].

**Figure 5:**
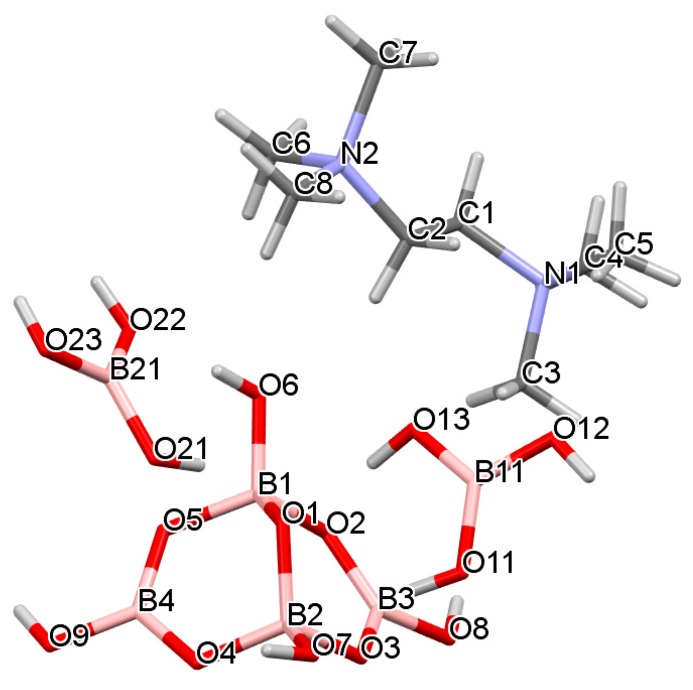
Drawing of the structure of **9** showing atomic labelling. Two water molecules (containing O31 and O32) are omitted for clarity. Compound **9** is disordered and only components with highest s.o.f are shown.

The tetraborate(2-) anion in **9** is found co-crystallized with 2B(OH)_3_ and 2H_2_O and the dication [Me_3_N(CH_2_)_2_NMe_3_]^2+^. The structure is disordered with the cation occupying three closely related positions with site occupancies of 47.4%, 28.7% and 23.9% and one of the two co-crystallized B(OH)_3_ units occupying 2 positions with site occupancies of 76% and 24%. A drawing of the atomic labelling for the components of highest site occupancy is given in Figure 5. Bond lengths and bond angles found for C, N atoms within the [Me_3_N(CH_2_)_2_NMe_3_]^2+^ cations are as expected and similar to those reported for the related pentaborate(1-) salt, [Me_3_N(CH_2_)_2_NMe_3_][B_5_O_6_(OH)_4_]_2_^.^ [28]. The B-O bond lengths, OBO and BOB angles observed for the [B_4_O_5_(OH)_4_]^2−^ and B(OH)_3_ motifs are in accord with previous tetraborate(2-) [47,48,49,50,51,52,53,54,55,56] and polyborates with co-crystallized B(OH)_3_ [32,33,39,55,57,58]; for full details see the Supplementary Information.

**Figure 6:**
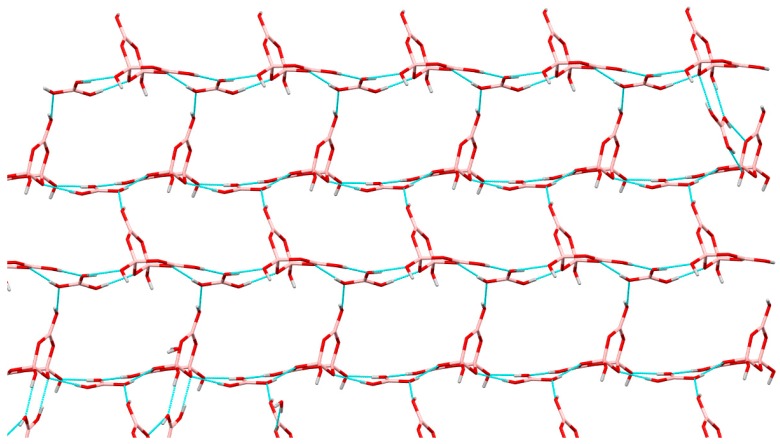
A 2D plane in **9** comprised of alternating B(OH)_3_/[B_4_O_5_(OH)_4_]^2−^ ′horizontal′ chains and ′vertical′ C_2_^2^(8) crosslinking.

**Figure 7:**
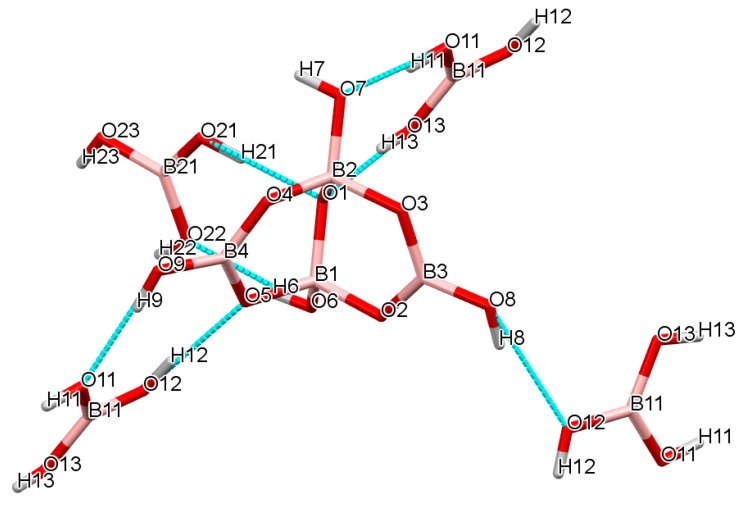
Drawing of the H-bond interactions around the tetraborate(2-) anion in **9**, showing three R_2_^2^(8) interactions and parts of the C_2_^2^(8) chain.

The tetraborate(2-) anion has four H-bond donor sites and nine potential H bond acceptor sites, and the B(OH)_3_ molecules each have three donor and three potential acceptor sites. Again, there are numerous H-bond interactions within the structure and a giant supramolecular anionic H-bonded lattice is formed, with cations situated within the cavities. However, there are no direct anion-anion interactions within the lattice and the B(OH)_3_ units serve to bridge tetraborate(2-) anions by acting as ′spacers′ to expand the lattice so that it can accommodate the relatively large cation, [Me_3_N(CH_2_)_2_NMe_3_]. This spacer role for B(OH)_3_ has been observed before [32,33,39,55,56,57,58]. With reference to Figure 6 it can be seen that the H-bond structure can be envisaged as ′horizontal′ chains of alternating [B_4_O_5_(OH)_4_]^2-^/B(OH)_3_ units held together by R_2_^2^(8) interaction, crosslinked by ′vertical′ C_2_^2^(8) chains (involving O8,H8^…^O12,H12^…^O5,B1,O2,B3) in a regular 2D arrangement. These planes are further linked by additional R_2_^2^(8) interactions into a 3D lattice. Further details of the interactions around the tetraborate(2-) anion is shown in Figure 7 and full details are available in the Supplementary Information. The O1 site is a double H-bond acceptor in two R_2_^2^(8) interactions: O21H21^…^O1 and O13H13^…^O1. The H_2_O of crystallization further help to H-bond the structure together. The cation is unable to get involved with H-bonding.

## 3. Materials and Methods 

### 3.1. General

All chemicals were obtained commercially. TGA/DSC analysis (in air) were undertaken on an SDT Q600 V4.1 Build 59 instrument (TA Instruments, New Castle, DE, USA) using Al_2_O_3_ crucibles, between 10–800 °C (ramp temperature rate of 10 °C min^−1^). FTIR spectra were obtained (KBr pellets) on a Perkin-Elmer 100 FTIR spectrometer (Perkin-Elmer, Seer Green, UK). NMR spectra were obtained on a Bruker Avance 400 spectrometer (Bruker, Coventry, UK) and reported in ppm with positive chemical shifts (δ) to high frequency (downfield) of TMS (^1^H, ^13^C) and BF_3_^.^OEt_2_ (^11^B). BET analysis were performed ion a Gemini 2375 analyser (Micromeritics Instrument Corporation, Norcross, GA, USA) with N_2_ gas as the adsorbent. CHN analysis were obtained from OEA Laboratories Ltd. (Callington, UK). See Section 3.11 for single-crystal XRD methods.

### 3.2. Synthesis, Spectroscopic, and Analytical Data for ***1***

B(OH)_3_ (6.2 g; 100.2 mmol) was dissolved in H_2_O (100 mL). To this solution was added Me_2_N(CH_2_)_2_NMe_2_ (1.18 g; 10.0 mmol) and the reaction mixture was stirred for 1 h. The solvent was partially removed by rotary evaporation to give white crystals. The crude product of **1** (5.5 g; 99%) was obtained after oven drying at 60 °C for 24 h. C_6_H_26_B_10_N_2_O_20_. Anal. Calc.: C = 13.0%, H = 4.7%, N = 5.1%. Found: C = 13.1%, H = 4.7%, N = 5.0%. TGA: 150–300 °C, condensation of pentaborate units with loss of 4H_2_O -14.0% (-13.0% calc.); 300–650 °C, oxidation of organic cation to leave residual 5B_2_O_3_ 62.5% (62.8% calc.). BET /m^2^g^-1^: condensed 0.94, pyrolysed 0.61. NMR. ^1^H/ppm: 2.47 (12H, s), 2.90 (4H, s). ^11^B: 1.1 (14%), 12.9 (29%), 19.2 (57%). ^13^C: 43.4 (CH_3_) 53.15 (CH_2_). IR (KBr/cm^−1^): 3412 s, 1424 s, 1300 s, 968 m, 928 m, 792 s, 706 m.

### 3.3. Synthesis, Spectroscopic, Analytical, and Crystallographic Data for ***2***

Following the method as described for **1**, B(OH)_3_ (6.2 g; 100.2 mmol) and Et_2_N(CH_2_)_2_NEt_2_ (1.74 g; 10.1 mmol) gave crude product of **2** (5.6 g; 91%). C_10_H_34_B_10_N_2_O_20_. Anal. Calc.: C = 19.6%, H = 5.6%, N = 4.6%. Found: C = 19.7%, H = 5.6%, N = 4.3%. Crystals suitable for single-crystal XRD studies were obtained by recrystallization of the crude product from H_2_O. TGA: 100–230 °C, condensation of pentaborate units with loss of 4H_2_O 12.0% (11.8% calc.); 230–650 °C, oxidation of organic cation to leave residual 5B_2_O_3_ 54.6% (55.3% calc.). BET/m^2^g^−1^: condensed 0.43, pyrolysed 0.36. NMR. ^1^H/ppm: 1.06 (12H, t), 2.90 (8H, q), 3.08 (4H, s). ^11^B: 1.1 (20%), 13.4 (30%), 18.6 (50%). ^13^C: 8.76 (CH_3_), 46.50 (4CH_2_), 47.28 (2CH_2_). IR (KBr/cm^−1^): 3445 s, 3143 s, 1316 m, 1230 s, 1101 s, 926 s, 783 m, 779 m. XRD crystallographic data: C_10_H_34_B_10_N_2_O_20,_ *M_r_* = 610.49, Triclinic, *P*−1, a = 8.3998(5) Å, b = 9.1406(7) Å, c = 18.2066(13) Å, *α* = 78.439(6)°, *β* = 86.810(5)°, *γ* = 88.118(6)°, *V* = 1367.07(17) Å^3^, *T* = 100(2) K, *Z* = 2, μ(MoKα) = 0.130 mm**^−^**^1^, 11455 reflections measured, 6146 unique (*R_int_* = 0.0461) which were used in all calculations. The final *wR_2_* was 0.1850 (all data) and *R_1_* was 0.0748 (*I* > 2σ(*I*)). 

### 3.4. Synthesis, Spectroscopic, Analytical, and Crystallographic Data for ***3***

Following the method as described for **1**, B(OH)_3_ (1.0 g; 16.2 mmol) and Et_2_NCH_2_NEt_2_ (0.26 g; 1.62 mmol) gave crude product of **3** (0.9 g; 99%). C_4_H_16_B_5_NO_10_. Anal. Calc.: C = 16.4%, H = 5.5%, N = 4.8%. Found: C = 16.7%, H = 5.6%, N = 4.7%. Crystals suitable for single-crystal XRD studies were obtained by recrystallization of the crude product from H_2_O. TGA: 100–230 °C, condensation of pentaborate units with loss of 2H_2_O 12.3% (14.3% calc.); 230–600 °C, oxidation of organic cation to leave residual 2.5B_2_O_3_ 58.3% (59.6% calc.). BET/m^2^g^-1^: pyrolysed 0.06. NMR. ^1^H/ppm: 1.16 (6H, t), 2.95 (4H, q). ^11^B: 1.2 (20%), 12.9 (25%), 18.3 (55%). ^13^C: 10.45 (CH_3_) 42.18 (CH_2_). IR (KBr/cm^−1^): 3436 s, 3377 s, 3228 m, 2977 m, 1435 s, 1361 s, 1251 s, 1103 s, 1026 s, 925 s, 882 m, 697 m. XRD crystallographic data: C_4_H_16_B_5_NO_10_, *M_r_* = 292.23, Triclinic, *P*−1, *a* = 8.3793(3) Å, *b* = 8.8187(4) Å, *c* = 10.0877(4) Å, *α* = 80.961(3)°, *β* = 76.057(3)°, *γ* = 68.269(4)°, *V* = 670.14(5) Å^3^, *T* = 100(2) K, *Z* = 2, μ(MoKα) = 0.129 mm**^−1^**, 5980 reflections measured, 3032 unique (*R_int_* = 0.0144) which were used in all calculations. The final *wR_2_* was 0.1024 (all data) and *R_1_* was 0.0363 (*I* > 2σ(*I*)).

### 3.5. Synthesis, Spectroscopic and Analytical Data for ***4***

Following the method as described for **1**, B(OH)_3_ (1.0 g; 16.2 mmol) and Me_2_NCH_2_NMe_2_ (0.17 g; 1.62 mmol) gave crude product of **4** (0.78 g; 92%). C_2_H_12_B_5_NO_10_. Anal. Calc.: C = 9.1%, H = 4.6%, N = 5.3%. Found: C = 9.4%, H = 4.6%, N = 5.3%. TGA: 100–230 °C, condensation of pentaborate units with loss of 2H_2_O 14.0% (13.6% calc.); 230–600 °C, oxidation of organic cation to leave residual 2.5B_2_O_3_ 65.6% (65.8% calc.). NMR. ^1^H/ppm: 2.59 (6H). ^11^B: 1.1 (15%), 13.2 (30%), 18.8 (55%). ^13^C: 34.42 (CH_3_). IR (KBr/cm^−1^): 3437 s, 3380 s, 3053 m, 1441 s, 1460 s, 1252 m, 1103 s, 1024 s, 925 s, 783 m, 765 m.

### 3.6. Synthesis, Spectroscopic and Analytical Data for ***5***

Following the method as described for **1**, B(OH)_3_ (6.2 g; 100.2 mmol) and Me_2_N(CH_2_)_3_NMe_2_ (1.30 g; 10.0 mmol) gave crude product of **5** (5.0 g; 88%). C_7_H_28_B_10_N_2_O_20_. Anal. Calc.: C = 14.8%, H = 5.0%, N = 4.9%. Found: C = 14.7%, H = 5.1%, N = 4.8%. TGA: 100–230 °C, condensation of pentaborate units with loss of 4H_2_O 13.2% (12.6% calc.); 230–630 °C, oxidation of organic cation to leave residual 5B_2_O_3_ 60.2% (61.2% calc.). BET/m^2^g^−1^: condensed 0.62. NMR. ^1^H/ppm: 3.03 (4H, t), 2.70 (12H, s), 2.05 (2H, m). ^11^B: 1.1 (20%), 13.2 (25%), 18.8 (55%). ^13^C: 42.8 (4CH_3_) 54.14 (2CH_2_), 19.9 (CH_2_). IR (KBr/cm^−1^): 3307 s, 2859 s, 1440 s, 1399 s, 1253 m, 1180 m, 1025 s, 924 s, 779 m.

### 3.7. Synthesis, Spectroscopic and Analytical Data for ***6***

Following the method as described for **1**, B(OH)_3_ (6.2 g; 100.2 mmol) and Et_2_N(CH_2_)_3_NEt_2_ (1.86 g; 10.0 mmol) gave crude product of **6** (5.4 g; 87%). C_11_H_36_B_10_N_2_O_20_. Anal. Calc.: C = 21.2%, H = 5.8%, N = 4.5%. Found: C = 21.4%, H = 5.9%, N = 4.4%. TGA: 100–230 °C, condensation of pentaborate units with loss of 4H_2_O 12.1% (11.5% calc.); 230–630 °C, oxidation of organic cation to leave residual 5B_2_O_3_ 55.3% (55.7% calc.). BET/m^2^g^−1^: condensed 0.43, pyrolysed 0.71. NMR. ^1^H/ppm: 1.13 (12H, t), 1.97 (2H, m), 3.05 (12H, m). ^11^B: 1.1 (25%), 12.7 (30%), 18.2 (45%). ^13^C: 43.4 (CH_3_) 53.15 (CH_2_). IR (KBr/cm^−1^): 3412 s, 1424 s, 1300 s, 968 m, 928 m, 792 s, 706 m.

### 3.8. Synthesis, Spectroscopic, Analytical, and Crystallographic Data for ***7***

Me_2_N(CH_2_)_3_NMe_2_ (2.6g, 20 mmol) dissolved in CH_3_CN (50 mL) was refluxed with MeI (11.4 g, 80 mmol) for 4 h. The white solid formed, [Me_3_N(CH_2_)_3_NMe_3_]I_2_ (8.28 g, 100%) was isolated by filtration, washed with Et_2_O and used without further purification. [NMR. ^1^H/ppm: 2.31 (2H, quin.), 3.13 (18H, s), 3.37 (4H, t). ^13^C: 17.41 (CH_2_), 53.25 (CH_3_), 62.39 (CH_2_). IR (KBr/cm^−1^): 3447s, 3011s, 2957m, 1624m, 1486s, 1475s, 1408m, 1244m, 1054m, 973s, 944s, 901s, 762m, 545m.] [Me_3_N(CH_2_)_3_NMe_3_]I_2_ (0.67 g, 1.62 mmol) was dissolved in H_2_O (20 mL) and stirred with excess (13.5 g) of DOWEX 550A ion-exchange resin (OH^-^ form) for 24 h. The slurry was filtered and B(OH)_3_ (1.0 g, 16.2 mmol) was added to the filtrate. The solution was left for 4 h before removal of solvent under reduced pressure at 85 °C to yield a ‘damp’ solid which was dried in an oven at 75 °C for 3 h to yield a white solid (0.93 g). ^1^H NMR analysis showed this to be a ~ 2:1 mixture of **7** and **8**. Recrystallization of 0.2 g of this solid resulted in 0.1g of **7** with crystals suitable for sc-XRD analysis. C_6_H_18_B_5_NO_10_. Anal. Calc.: C = 22.6%, H = 5.7%, N = 4.4%. Found: C = 23.0%, H = 5.7%, N = 4.4%. TGA: 100–285 °C, condensation of pentaborate units with loss of 2H_2_O 11.7% (11.3% calc.); 285–700 °C, oxidation of organic cation to leave residual 2.5B_2_O_3_ 55.2% (54.7% calc.). NMR. ^1^H/ppm: 3.00 (9H, s), 3.85 (2H, d), 5.63 (2H, dd), 5.96 (1H, m). ^11^B: 1.1 (1%), 13.4 (17%), 17.1 (82%). ^13^C: 52.30 (CH_3_), 68.39 (CH_2_), 124.43 (CH), 129.14 (CH_2_). IR (KBr/cm^−1^): 3437 s, 3262 m, 1471 m, 1427 s, 1414 s, 1389 s, 1311 s, 1168 m, 1105 s, 1012 s, 921 s, 777 s, 724 m, 708 s. XRD crystallographic data: C_6_H_18_B_5_NO_10,_ *M*r = 318.26, monoclinic, *P*2_1_/*c*, *a* = 9.54050(10) Å, *b* = 16.1031(2) Å, *c* = 9.43280(10) Å, α = γ = 90^o^, β = 90.1710(10)^o^, *V* = 1449.17(3) Å^3^, *T* = 100(2) K, *Z* = 4, *Z*’ = 1, μ(CuKα) = 1.096 mm**^−1^**, 13531 reflections measured, 2661 unique (*R_int_* = 0.0236) which were used in calculations. The final *wR_2_* was 0.0845 (all data) and *R_1_* was 0.0314 (I > 2σ(I)).

### 3.9. Synthesis, Spectroscopic, Analytical, and Crystallographic Data for ***8***

[Me_3_N(CH_2_)_3_NMe_3_]I_2_ (0.40 g, 0.97 mmol), prepared as described for **7**, was dissolved in H_2_O (20 mL) and stirred with excess (8.0 g) of DOWEX 550A ion-exchange resin (OH^-^ form) for 24 h. The slurry was filtered and B(OH)_3_ (0.6 g, 9.7 mmol) was added to the filtrate. Partial evaporation of the solution afforded a white crystalline solid (0.57 g, 96%), separated by filtration. These crystals were suitable for sc-XRD studies. C_9_H_33_B_10_N_2_O_20.5_. Anal. Calc.: C = 17.9%, H = 5.5%, N = 4.6%. Found: C = 18.2%, H = 5.4%, N = 4.6%. TGA: 250–300 °C, condensation of pentaborate units with loss of 4.5 H_2_O 13.0% (13.4% calc.); 300–700 °C, oxidation of organic cation to leave residual 5B_2_O_3_ 57.3% (57.5% calc.). NMR. ^1^H/ppm: 2.29 (2H, quint.), 3.11 (18H, s), 3.33 (4H, t). ^11^B: 1.1 (3%), 13.1 (35%), 18.0 (62%). ^13^C: 17.28 (CH_2_), 53.15 (CH_3_), 62.37 (CH_2_). IR (KBr/cm^−1^): 3437 s, 3082 s, 1638 m, 1438 m, 1359 s, 1251 m, 1102 s, 1026 m, 926 s, 782 m, 696 m. XRD crystallographic data: C_9_H_33_B_10_N_2_O_20.5_. *M*r = 605.47, monoclinic, *C*2/*c*, *a* = 26.8754(5) Å, *b* = 11.5269(2) Å, *c* = 17.9383(4) Å, α = γ = 90^o^, β =103.154(2)^o^, *V* = 5411.30(19) Å^3^, *T* = 100(2) K, *Z* = 8, *Z*’ = 1, μ(MoKα) = 0.132 mm**^−1^**, 31515 reflections measured, 6191 unique (*R_int_* = 0.0294) which were used in calculations. The final *wR_2_* was 0.0919 (all data) and *R_1_* was 0.0351 (I > 2σ(I)).

### 3.10. Synthesis, Spectroscopic, Analytical, and Crystallographic Data for ***9***

[Me_3_N(CH_2_)_2_NMe_3_]I_2_ (1.078 g, 2.7 mmol) [28] was dissolved in H_2_O (20 mL) and stirred with excess (15.0 g) of DOWEX 550A ion-exchange resin (OH^−^ form) for 24 h. The slurry was filtered and B(OH)_3_ (0.5 g, 8.1 mmol) was added to the filtrate. The solution was left stand for 4 h and concentrated to ca. 7 mL under reduced pressure. The solution was left to stand in a small sample vial for 7 days to yield a crystalline product (0.277 g). These crystals were suitable for sc-XRD studies. C_8_H_36_B_6_N_2_O_17_. Anal. Calc.: C = 19.3%, H = 7.3%, N = 5.6%. Found: C = 20.6%, H = 7.3%, N = 5.7%. TGA: 100–200 °C, condensation of tetraborate/B(OH)_3_ units and loss of 2 interstitial H_2_O (total 7H_2_O) 25.7% (25.3% calc.); 200–800 °C, oxidation of organic cation to leave residual 3B_2_O_3_ 32.3% (29.0% calc.). NMR. ^1^H/ppm: 3.00 (18H, s), 3.85 (H, m). ^11^B: 1.3 (5%), 7.4 (43%), 11.7 (52%). ^13^C: 53.82 (CH_3_) 57.77 (CH_2_). IR (KBr/cm^−1^): 3435 s, 1638 m, 1481 s, 1418 s, 1385 m, 1352 m, 1070 m, 958 s, 927 m. XRD crystallographic data: C_8_H_36_B_6_N_2_O_17_, *M*r = 497.25, monoclinic, *P*2_1_*, a* = 9.0242(2) Å, *b* = 12.0350(3) Å, *c* = 11.1688(4) Å, α = γ = 90^o^, β = 109.811(3)^o^, *V* = 1141.21(6) Å^3^, *T* = 100(2) K, *Z* = 2, *Z*’ = 1, μ(MoKα) = 0.131 mm**^−1^**, 24237 reflections measured, 5229 unique (*R_int_* = 0.0265) which were used in calculations. The final *wR_2_* was 0.0802 (all data) and *R_1_* was 0.0303 (I > 2σ(I)).

### 3.11. X-Ray Crystallography

Single-crystal X-ray crystallography (sc-XRD) was undertaken at the EPSRC National Crystallography Service at the University of Southampton. Selected crystals were mounted on a MITIGEN holder in perfluoroether oil. Suitable crystals of **2**, **3** and **8** were placed on a FRE+ instrument Rigaku (Wilmington, MA, USA) equipped with HF Varimax confocal mirrors and an AFC12 goniometer and HG Saturn 724+ detector diffractometer. Suitable crystals of **7** were placed on a Rigaku 007HF equipped with HF Varimax confocal mirrors and an AFC11 goniometer and HyPix 6000 detector diffractometer. Suitable crystals of **9** were placed on a Rigaku FRE+ equipped with VHF Varimax confocal mirrors and an AFC12 goniometer and HyPix 6000 diffractometer. The crystals were kept at *T* = 100(2) K during data collection. The structures were solved with ShelXT [59] using Olex2 [60]. The models were refined with ShelXL [61] (using Least Squares minimisation). CCDC: 1968475 (**2**), 1968476 (**3**), 1968477 (**7**), 1968478 (**8**) and 1968479 (**9**) contains the supplementary crystallographic data for this paper. These data can be obtained free of charge via http://www.ccdc.cam.ac.uk/conts/retrieving.html (or from the CCDC, 12 Union Road, Cambridge CB2 1EZ, UK; Fax: +44 1223 336033; E-mail: deposit@ccdc.cam.ac.uk) 

## 4. Conclusions

Six new pentaborate(1-) and one new tetraborate(2-) salts were synthesized by templated crystallization reactions from aqueous solutions containing B(OH)_3_ and the appropriate C_2_-linked or C_3_-linked bis(alkylammonium)(2+) cations. [Me_3_NCH_2_CH=CH_2_][B_5_O_6_(OH)_4_] was obtained in addition to [Me_3_N(CH_2_)_3_NMe_3_][B_5_O_6_(OH)_4_]_2_^.^0.5H_2_O, as a result of a Hofmann elimination during work up, from the reaction of B(OH)_3_ with [Me_3_N(CH_2_)_3_NMe_3_](OH)_2_ (10:1). All solid-state structures contain supramolecular H-bonded giant structures with cations situated within the cavities. The R_2_^2^(8) H-bonding motif is found to dominate H-bonding interactions within all structures and the role of B(OH)_3_ in [Me_3_N(CH_2_)_2_NMe_3_][B_4_O_5_(OH)_4_]^.^2B(OH)_3_^.^2H_2_O appears to be to expand the lattice in order to accommodate the large cationic counterion.

## Supplementary Materials

The following are available online at https://www.mdpi.com/1420-3049/25/1/53/s1, Single crystal XRD data, including Figures of the structures with numerous H-bond interactions.
