# Supplementary material for: Pentaborate(1-) Salts and a Tetraborate(2-) Salt Derived from C2- or C3-Linked Bis(alkylammonium) Dications: Synthesis, Characterization, and Structural (XRD) Studies
Source: Molecules. 2019 Dec 23;25(1):53. doi: 10.3390/molecules25010053 (PMC6982793; doi:10.3390/molecules25010053)
Supplement: Supplementary file 1 [file molecules-25-00053-s001.zip › MAB2.docx]

**MAB2**


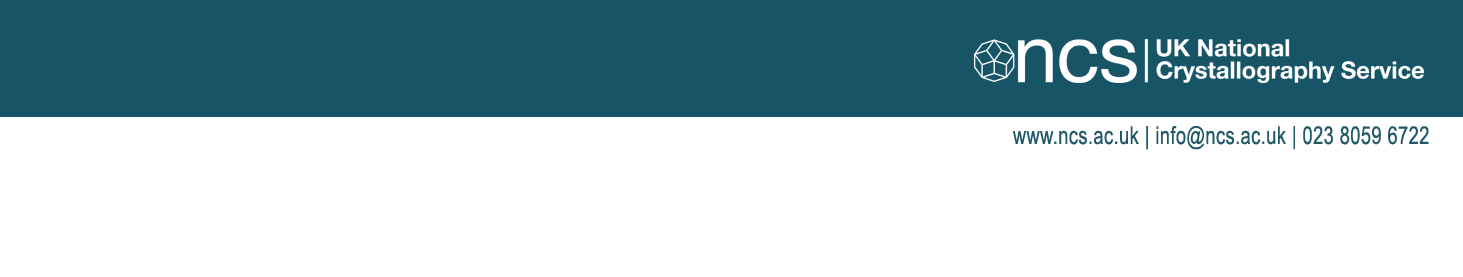


Submitted by: **None**

None

Solved by: **None**

Sample ID: **MAB2**

***R_1_*=7.48%**

Crystal Data and Experimental


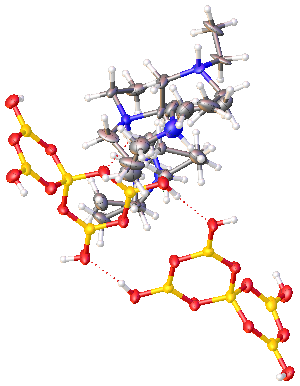


**Experimental.** Single colourless plate crystals of **MAB2** recrystallised from water. A suitable crystal with dimensions 0.100 × 0.040 × 0.010 mm^3^ was selected and mounted on a Rigaku FRE+ equipped with VHF Varimax confocal mirrors and an AFC12 goniometer and HG Saturn 724+ detector diffractometer. The crystal was kept at a steady *T* = 100(2) K during data collection. The structure was solved with the **ShelXT** 2018/2 (Sheldrick, 2018) solution program using dual methods and by using **Olex2** (Dolomanov et al., 2009) as the graphical interface. The model was refined with **ShelXL** 2018/3 (Sheldrick, 2015) using full matrix least squares minimisation on ***F*^2^**.

**Crystal Data.** C_10_H_34_B_10_N_2_O_20_, *M_r_* = 610.49, triclinic, *P*-1 (No. 2), a = 8.3998(5) Å, b = 9.1406(7) Å, c = 18.2066(13) Å, *α* = 78.439(6)^°^, *β* = 86.810(5)^°^, *γ* = 88.118(6)^°^, *V* = 1367.07(17) Å^3^, *T* = 100(2) K, *Z* = 2, *Z'* = 1, *μ*(Mo K*_α_*) = 0.130, 11455 reflections measured, 6146 unique (*R_int_* = 0.0461) which were used in all calculations. The final *wR_2_* was 0.1850 (all data) and *R_1_* was 0.0748 (I > 2(I)).

| **Compound** | **MAB2** |
| --- | --- |
|  |  |
| Formula | C_10_H_34_B_10_N_2_O_20_ |
| *D_calc._*/ g cm^-3^ | 1.483 |
| *μ*/mm^-1^ | 0.130 |
| Formula Weight | 610.49 |
| Colour | colourless |
| Shape | plate |
| Size/mm^3^ | 0.100×0.040×0.010 |
| *T*/K | 100(2) |
| Crystal System | triclinic |
| Space Group | *P*-1 |
| *a*/Å | 8.3998(5) |
| *b*/Å | 9.1406(7) |
| *c*/Å | 18.2066(13) |
| *α*/^°^ | 78.439(6) |
| *β*/^°^ | 86.810(5) |
| *γ*/^°^ | 88.118(6) |
| V/Å^3^ | 1367.07(17) |
| *Z* | 2 |
| *Z'* | 1 |
| Wavelength/Å | 0.71075 |
| Radiation type | Mo K*_α_* |
| *Θ_min_*/^°^ | 2.275 |
| *Θ_max_*/^°^ | 27.486 |
| Measured Refl's. | 11455 |
| Ind't Refl's | 6146 |
| Refl's with I > 2(I) | 2997 |
| *R_int_* | 0.0461 |
| Parameters | 561 |
| Restraints | 388 |
| Largest Peak | 0.485 |
| Deepest Hole | -0.255 |
| GooF | 1.005 |
| *wR_2_* (all data) | 0.1850 |
| *wR_2_* | 0.1460 |
| *R_1_* (all data) | 0.1666 |
| *R_1_* | 0.0748 |

**Table 1**: Fractional Atomic Coordinates (×10^4^) and Equivalent Isotropic Displacement Parameters (Å^2^×10^3^) for **MAB2**. *U_eq_* is defined as 1/3 of the trace of the orthogonalised *U_ij_*.

| **Atom** | **x** | **y** | **z** | ***U_eq_*** |
| --- | --- | --- | --- | --- |
| B1 | 8885(4) | 2797(5) | 1428(2) | 27.8(9) |
| B2 | 7699(4) | 4341(4) | 2296(2) | 21.7(8) |
| B3 | 5982(4) | 3073(4) | 1643(2) | 21.3(8) |
| B4 | 10893(4) | 775(5) | 1366(2) | 31.3(10) |
| B5 | 10880(4) | 2883(5) | 363(2) | 31.4(10) |
| O1 | 8986(2) | 3796(3) | 1968.9(12) | 25.3(5) |
| O2 | 6182(2) | 3988(3) | 2145.0(13) | 30.9(6) |
| O3 | 7248(2) | 2510(3) | 1299.7(12) | 29.0(6) |
| O4 | 9719(2) | 1394(3) | 1741.0(12) | 29.5(6) |
| O5 | 11456(2) | 1457(3) | 656.1(13) | 34.2(6) |
| O6 | 9703(2) | 3541(3) | 719.9(12) | 29.1(6) |
| O7 | 7810(2) | 5276(3) | 2787.2(13) | 35.9(7) |
| O8 | 4486(2) | 2768(3) | 1485.2(13) | 32.2(6) |
| O9 | 11605(3) | -562(3) | 1660.5(14) | 40.3(7) |
| O10 | 11589(3) | 3585(3) | -301.7(13) | 39.3(7) |
| B11 | 1000(4) | 6923(5) | 3465(2) | 29.0(10) |
| B12 | -981(4) | 8995(5) | 3457(2) | 30.2(10) |
| B13 | -1017(4) | 6970(5) | 4515(2) | 29.2(10) |
| B14 | 2175(4) | 5119(4) | 2722(2) | 24.4(9) |
| B15 | 3911(4) | 6444(4) | 3338(2) | 24.7(9) |
| O11 | 221(2) | 8328(3) | 3111.8(12) | 30.0(6) |
| O12 | -1586(2) | 8354(3) | 4167.0(13) | 31.5(6) |
| O13 | 174(2) | 6268(3) | 4185.1(12) | 30.4(6) |
| O14 | 894(2) | 5853(2) | 2961.2(12) | 26.3(6) |
| O15 | 3677(2) | 5378(3) | 2918.2(13) | 32.2(6) |
| O16 | 2654(2) | 7190(3) | 3595.5(13) | 33.6(6) |
| O17 | -1627(3) | 10346(3) | 3149.5(14) | 36.6(6) |
| O18 | -1687(3) | 6391(3) | 5203.9(13) | 35.9(6) |
| O19 | 2041(2) | 4124(3) | 2270.0(14) | 40.7(7) |
| O20 | 5395(2) | 6763(3) | 3498.6(13) | 32.5(6) |
| C1 | 4957(8) | 9163(7) | 71(4) | 23.5(19) |
| C2 | 4379(8) | 6848(7) | 1011(4) | 24.2(18) |
| C3 | 2956(14) | 6378(12) | 651(9) | 42(3) |
| C4 | 5452(7) | 9045(10) | 1455(4) | 27.0(19) |
| C5 | 7217(11) | 8715(16) | 1356(6) | 38(3) |
| N1 | 4458(7) | 8547(6) | 883(3) | 23.8(16) |
| C11 | 5030(8) | 9789(8) | 418(3) | 31.3(19) |
| C12 | 4607(7) | 7034(8) | 407(4) | 37.1(19) |
| C13 | 2870(11) | 7110(13) | 624(8) | 46(3) |
| C14 | 5700(8) | 7857(9) | 1513(3) | 34.1(18) |
| C15 | 7140(12) | 8559(14) | 1739(5) | 46(3) |
| N11 | 5575(7) | 8194(6) | 670(3) | 31.3(16) |
| C21 | 5104(14) | 9308(14) | 4804(8) | 42(3) |
| C22 | 2632(19) | 8093(15) | 5192(8) | 46(3) |
| C23 | 3720(20) | 6810(15) | 5477(8) | 61(3) |
| C24 | 2866(12) | 10394(15) | 4031(6) | 30(3) |
| C25 | 4022(13) | 10772(12) | 3337(5) | 44(3) |
| N21 | 3503(17) | 9031(15) | 4525(8) | 26(3) |
| C31 | 5011(14) | 10305(16) | 5369(7) | 44(3) |
| C32 | 7730(20) | 11480(13) | 4789(9) | 45(3) |
| C33 | 7383(13) | 12965(11) | 4349(5) | 46(3) |
| C34 | 7744(14) | 9579(16) | 5815(7) | 42(3) |
| C35 | 7065(17) | 8802(16) | 6578(7) | 62(4) |
| N31 | 6651(16) | 10842(15) | 5453(7) | 25(3) |
| C41 | 5530(30) | 9340(20) | 4965(11) | 49(5) |
| C42 | 4010(30) | 7370(30) | 5884(17) | 59(6) |
| C43 | 3690(60) | 6500(60) | 5300(30) | 62(9) |
| C44 | 6000(30) | 8680(40) | 6391(14) | 58(6) |
| C45 | 7760(30) | 8950(50) | 6390(20) | 43(7) |
| N41 | 5560(30) | 8193(17) | 5686(11) | 64(5) |

**Table 2**: Anisotropic Displacement Parameters (×10^4^) for **MAB2**. The anisotropic displacement factor exponent takes the form: *-2π^2^[h^2^a*^2^ × U_11_+ ... +2hka* × b* × U_12_]*

| **Atom** | ***U_11_*** | ***U_22_*** | ***U_33_*** | ***U_23_*** | ***U_13_*** | ***U_12_*** |
| --- | --- | --- | --- | --- | --- | --- |
| B1 | 17.3(19) | 35(3) | 36(2) | -19(2) | 0.7(16) | -0.7(16) |
| B2 | 23(2) | 18(2) | 25(2) | -6.2(17) | 2.4(15) | -1.2(15) |
| B3 | 20.1(19) | 23(2) | 23(2) | -9.5(17) | 1.2(15) | 0.4(15) |
| B4 | 19(2) | 36(3) | 46(3) | -26(2) | 2.4(18) | -2.9(17) |
| B5 | 23(2) | 45(3) | 33(2) | -22(2) | 1.5(17) | -3.3(18) |
| O1 | 20.3(12) | 28.6(15) | 31.0(13) | -16.7(11) | 3.4(9) | -3.2(10) |
| O2 | 17.8(12) | 37.2(16) | 45.2(15) | -27.1(12) | 3.3(10) | -2.6(10) |
| O3 | 19.4(12) | 37.8(16) | 36.5(14) | -24.3(12) | 2.4(10) | -2.6(10) |
| O4 | 18.9(12) | 32.3(16) | 41.0(15) | -18.3(12) | 6.2(10) | -2.3(10) |
| O5 | 23.6(13) | 39.2(17) | 44.6(16) | -22.0(13) | 5.9(11) | 1.4(11) |
| O6 | 21.7(12) | 39.5(16) | 28.5(13) | -14.5(11) | 5.1(10) | -0.3(10) |
| O7 | 16.9(12) | 42.3(17) | 58.0(17) | -34.4(14) | 5.2(11) | -4.3(11) |
| O8 | 18.0(12) | 41.2(17) | 44.5(16) | -26.5(13) | -0.2(10) | 1.8(11) |
| O9 | 28.1(14) | 37.5(18) | 56.3(18) | -16.8(14) | 13.8(12) | 4.1(12) |
| O10 | 31.0(15) | 50(2) | 37.7(15) | -13.7(13) | 12.9(11) | 2.7(12) |
| B11 | 20(2) | 34(3) | 39(2) | -21(2) | 2.6(17) | -1.8(17) |
| B12 | 20(2) | 34(3) | 42(3) | -21(2) | -0.8(17) | -2.1(17) |
| B13 | 17(2) | 37(3) | 39(3) | -22(2) | -0.5(17) | -4.3(17) |
| B14 | 22(2) | 24(2) | 28(2) | -8.4(18) | 3.2(15) | -0.1(16) |
| B15 | 23(2) | 24(2) | 29(2) | -11.0(18) | 2.3(16) | -2.3(16) |
| O11 | 23.2(12) | 26.5(15) | 43.6(15) | -16.7(12) | 7.1(10) | -3.3(10) |
| O12 | 22.1(12) | 32.5(16) | 43.5(16) | -19.0(12) | 5.3(10) | 3.0(10) |
| O13 | 22.5(12) | 33.0(16) | 39.0(14) | -16.8(12) | 5.4(10) | -0.1(10) |
| O14 | 19.5(12) | 26.3(15) | 38.0(14) | -18.9(11) | 3.1(10) | -3.4(10) |
| O15 | 19.4(12) | 38.8(17) | 46.6(15) | -28.8(13) | 2.0(10) | -0.8(10) |
| O16 | 21.4(12) | 36.6(17) | 51.8(16) | -31.3(13) | 2.3(10) | -3.4(10) |
| O17 | 31.2(14) | 35.3(17) | 44.7(16) | -14.8(13) | 6.2(11) | 6.7(12) |
| O18 | 32.0(14) | 34.0(18) | 42.9(16) | -14.3(12) | 8.6(12) | 5.5(11) |
| O19 | 18.9(13) | 54.0(19) | 61.7(18) | -42.2(15) | 0.8(12) | -0.6(12) |
| O20 | 20.9(13) | 37.0(17) | 46.9(16) | -26.6(12) | 1.9(11) | -3.4(11) |
| C1 | 24(4) | 30(5) | 20(5) | -15(5) | 5(3) | -1(3) |
| C2 | 29(4) | 15(4) | 32(5) | -14(3) | 2(3) | -3(3) |
| C3 | 52(6) | 30(7) | 45(6) | -7(7) | -14(4) | -16(6) |
| C4 | 23(4) | 35(6) | 27(4) | -15(4) | -1(3) | 1(3) |
| C5 | 36(5) | 41(7) | 42(7) | -21(7) | -6(5) | 1(4) |
| N1 | 23(4) | 26(4) | 26(3) | -13(3) | 5(3) | -3(3) |
| C11 | 27(4) | 41(6) | 26(5) | -8(5) | 4(3) | 0(3) |
| C12 | 40(4) | 34(5) | 41(5) | -19(4) | -3(3) | -3(3) |
| C13 | 37(5) | 59(9) | 48(6) | -25(8) | -2(4) | -12(6) |
| C14 | 38(4) | 30(5) | 35(4) | -11(4) | 0(3) | -1(3) |
| C15 | 54(6) | 39(6) | 47(7) | -4(7) | -14(6) | -13(4) |
| N11 | 26(3) | 39(4) | 34(4) | -19(3) | 1(3) | -3(3) |
| C21 | 22(5) | 54(7) | 63(6) | -40(5) | -22(4) | 17(4) |
| C22 | 77(7) | 41(8) | 28(5) | -28(5) | 12(5) | -26(5) |
| C23 | 109(10) | 32(7) | 45(9) | -16(5) | 2(7) | -19(6) |
| C24 | 25(6) | 30(5) | 43(6) | -20(4) | -14(4) | 10(5) |
| C25 | 66(7) | 29(6) | 38(5) | -12(4) | 2(5) | 11(5) |
| N21 | 18(5) | 25(6) | 41(6) | -17(4) | -4(4) | -1(4) |
| C31 | 29(5) | 50(7) | 67(7) | -42(6) | -10(5) | 12(4) |
| C32 | 72(8) | 28(6) | 42(6) | -25(5) | 24(5) | -33(5) |
| C33 | 69(7) | 39(6) | 33(6) | -13(5) | 3(5) | -38(5) |
| C34 | 42(7) | 25(6) | 65(7) | -23(5) | -16(5) | 10(5) |
| C35 | 90(11) | 33(7) | 65(8) | -4(6) | -21(8) | -15(8) |
| N31 | 27(5) | 25(6) | 29(5) | -19(4) | 8(4) | 2(4) |
| C41 | 37(10) | 53(10) | 62(9) | -26(8) | -12(9) | 21(9) |
| C42 | 69(10) | 58(12) | 53(11) | -23(9) | 6(10) | 3(9) |
| C43 | 79(18) | 59(18) | 51(16) | -16(14) | -15(15) | 0(14) |
| C44 | 55(11) | 56(12) | 63(10) | -9(10) | -9(10) | 13(10) |
| C45 | 50(12) | 50(18) | 27(15) | -6(14) | -1(12) | 18(12) |
| N41 | 59(8) | 61(9) | 74(8) | -19(7) | -11(7) | 8(7) |

**Table 3**: Bond Lengths in Å for **MAB2**.

| **Atom** | **Atom** | **Length/Å** |
| --- | --- | --- |
| B1 | O1 | 1.479(4) |
| B1 | O3 | 1.448(4) |
| B1 | O4 | 1.468(5) |
| B1 | O6 | 1.476(5) |
| B2 | O1 | 1.335(4) |
| B2 | O2 | 1.381(4) |
| B2 | O7 | 1.364(4) |
| B3 | O2 | 1.377(4) |
| B3 | O3 | 1.344(4) |
| B3 | O8 | 1.354(4) |
| B4 | O4 | 1.342(4) |
| B4 | O5 | 1.383(5) |
| B4 | O9 | 1.366(5) |
| B5 | O5 | 1.388(5) |
| B5 | O6 | 1.345(4) |
| B5 | O10 | 1.365(5) |
| B11 | O11 | 1.467(5) |
| B11 | O13 | 1.473(5) |
| B11 | O14 | 1.476(4) |
| B11 | O16 | 1.459(4) |
| B12 | O11 | 1.352(4) |
| B12 | O12 | 1.384(5) |
| B12 | O17 | 1.358(5) |
| B13 | O12 | 1.380(5) |
| B13 | O13 | 1.348(4) |
| B13 | O18 | 1.359(5) |
| B14 | O14 | 1.347(4) |
| B14 | O15 | 1.371(4) |
| B14 | O19 | 1.355(4) |
| B15 | O15 | 1.378(4) |
| B15 | O16 | 1.351(4) |
| B15 | O20 | 1.351(4) |
| C1 | C1^1^ | 1.503(12) |
| C1 | N1 | 1.514(8) |
| C2 | C3 | 1.508(11) |
| C2 | N1 | 1.527(8) |
| C4 | C5 | 1.511(10) |
| C4 | N1 | 1.517(7) |
| C11 | C11^1^ | 1.496(11) |
| C11 | N11 | 1.502(8) |
| C12 | C13 | 1.493(10) |
| C12 | N11 | 1.523(7) |
| C14 | C15 | 1.502(10) |
| C14 | N11 | 1.512(7) |
| C21 | N21 | 1.509(11) |
| C21 | C31 | 1.502(8) |
| C22 | C23 | 1.489(14) |
| C22 | N21 | 1.507(11) |
| C24 | C25 | 1.539(10) |
| C24 | N21 | 1.487(11) |
| C31 | N31 | 1.503(11) |
| C32 | C33 | 1.461(12) |
| C32 | N31 | 1.503(11) |
| C34 | C35 | 1.516(12) |
| C34 | N31 | 1.517(12) |
| C41 | C41^2^ | 1.50(2) |
| C41 | N41 | 1.507(15) |
| C42 | C43 | 1.490(17) |
| C42 | N41 | 1.514(15) |
| C44 | C45 | 1.505(16) |
| C44 | N41 | 1.507(15) |

––––

^1^1-x,2-y,-z; ^2^1-x,2-y,1-z

**Table 4**: Bond Angles in ^°^ for **MAB2**.

| **Atom** | **Atom** | **Atom** | **Angle/^°^** |
| --- | --- | --- | --- |
| O3 | B1 | O1 | 111.9(3) |
| O3 | B1 | O4 | 110.1(3) |
| O3 | B1 | O6 | 110.1(3) |
| O4 | B1 | O1 | 107.2(3) |
| O4 | B1 | O6 | 110.5(3) |
| O6 | B1 | O1 | 107.0(3) |
| O1 | B2 | O2 | 121.1(3) |
| O1 | B2 | O7 | 122.2(3) |
| O7 | B2 | O2 | 116.7(3) |
| O3 | B3 | O2 | 120.8(3) |
| O3 | B3 | O8 | 120.0(3) |
| O8 | B3 | O2 | 119.2(3) |
| O4 | B4 | O5 | 121.9(4) |
| O4 | B4 | O9 | 122.2(4) |
| O9 | B4 | O5 | 115.9(3) |
| O6 | B5 | O5 | 121.7(4) |
| O6 | B5 | O10 | 122.0(4) |
| O10 | B5 | O5 | 116.3(3) |
| B2 | O1 | B1 | 122.8(3) |
| B3 | O2 | B2 | 119.8(2) |
| B3 | O3 | B1 | 123.6(3) |
| B4 | O4 | B1 | 123.5(3) |
| B4 | O5 | B5 | 118.0(3) |
| B5 | O6 | B1 | 123.3(3) |
| O11 | B11 | O13 | 111.2(3) |
| O11 | B11 | O14 | 107.9(3) |
| O13 | B11 | O14 | 107.4(3) |
| O16 | B11 | O11 | 109.7(3) |
| O16 | B11 | O13 | 109.1(3) |
| O16 | B11 | O14 | 111.5(3) |
| O11 | B12 | O12 | 121.3(4) |
| O11 | B12 | O17 | 122.9(4) |
| O17 | B12 | O12 | 115.8(3) |
| O13 | B13 | O12 | 120.7(3) |
| O13 | B13 | O18 | 122.8(4) |
| O18 | B13 | O12 | 116.5(3) |
| O14 | B14 | O15 | 120.8(3) |
| O14 | B14 | O19 | 121.8(3) |
| O19 | B14 | O15 | 117.4(3) |
| O16 | B15 | O15 | 120.4(3) |
| O20 | B15 | O15 | 120.8(3) |
| O20 | B15 | O16 | 118.8(3) |
| B12 | O11 | B11 | 123.0(3) |
| B13 | O12 | B12 | 119.6(3) |
| B13 | O13 | B11 | 123.7(3) |
| B14 | O14 | B11 | 123.0(3) |
| B14 | O15 | B15 | 120.4(3) |
| B15 | O16 | B11 | 123.5(3) |
| C1^1^ | C1 | N1 | 110.6(8) |
| C3 | C2 | N1 | 111.1(6) |
| C5 | C4 | N1 | 113.8(6) |
| C1 | N1 | C2 | 109.5(5) |
| C1 | N1 | C4 | 115.1(5) |
| C4 | N1 | C2 | 111.8(5) |
| C11^1^ | C11 | N11 | 111.9(8) |
| C13 | C12 | N11 | 113.4(6) |
| C15 | C14 | N11 | 111.4(6) |
| C11 | N11 | C12 | 115.5(5) |
| C11 | N11 | C14 | 109.5(5) |
| C14 | N11 | C12 | 111.9(5) |
| C31 | C21 | N21 | 113.6(9) |
| C23 | C22 | N21 | 106.5(10) |
| N21 | C24 | C25 | 107.6(9) |
| C22 | N21 | C21 | 104.9(10) |
| C24 | N21 | C21 | 111.2(9) |
| C24 | N21 | C22 | 126.3(13) |
| C21 | C31 | N31 | 109.2(9) |
| C33 | C32 | N31 | 119.7(10) |
| C35 | C34 | N31 | 111.6(10) |
| C31 | N31 | C34 | 111.9(10) |
| C32 | N31 | C31 | 122.3(11) |
| C32 | N31 | C34 | 98.2(11) |
| C41^2^ | C41 | N41 | 112.5(16) |
| C43 | C42 | N41 | 110(2) |
| C45 | C44 | N41 | 112(2) |
| C41 | N41 | C42 | 113.4(17) |
| C44 | N41 | C41 | 118.7(18) |
| C44 | N41 | C42 | 106.0(16) |

––––

^1^1-x,2-y,-z; ^2^1-x,2-y,1-z

**Table 5**: Torsion Angles in ^°^ for **MAB2**.

| **Atom** | **Atom** | **Atom** | **Atom** | **Angle/^°^** |
| --- | --- | --- | --- | --- |
| O1 | B1 | O3 | B3 | -0.9(5) |
| O1 | B1 | O4 | B4 | -126.4(3) |
| O1 | B1 | O6 | B5 | 126.5(3) |
| O1 | B2 | O2 | B3 | -0.8(5) |
| O2 | B2 | O1 | B1 | 0.3(5) |
| O2 | B3 | O3 | B1 | 0.5(5) |
| O3 | B1 | O1 | B2 | 0.5(5) |
| O3 | B1 | O4 | B4 | 111.6(3) |
| O3 | B1 | O6 | B5 | -111.6(3) |
| O3 | B3 | O2 | B2 | 0.4(5) |
| O4 | B1 | O1 | B2 | -120.3(3) |
| O4 | B1 | O3 | B3 | 118.2(3) |
| O4 | B1 | O6 | B5 | 10.2(4) |
| O4 | B4 | O5 | B5 | 6.1(5) |
| O5 | B4 | O4 | B1 | 2.6(5) |
| O5 | B5 | O6 | B1 | -2.7(5) |
| O6 | B1 | O1 | B2 | 121.2(3) |
| O6 | B1 | O3 | B3 | -119.7(3) |
| O6 | B1 | O4 | B4 | -10.2(4) |
| O6 | B5 | O5 | B4 | -6.1(5) |
| O7 | B2 | O1 | B1 | -179.2(3) |
| O7 | B2 | O2 | B3 | 178.8(3) |
| O8 | B3 | O2 | B2 | -178.3(3) |
| O8 | B3 | O3 | B1 | 179.1(3) |
| O9 | B4 | O4 | B1 | -177.4(3) |
| O9 | B4 | O5 | B5 | -173.9(3) |
| O10 | B5 | O5 | B4 | 173.0(3) |
| O10 | B5 | O6 | B1 | 178.3(3) |
| O11 | B11 | O13 | B13 | -7.6(4) |
| O11 | B11 | O14 | B14 | 126.7(3) |
| O11 | B11 | O16 | B15 | -124.9(3) |
| O11 | B12 | O12 | B13 | -3.6(5) |
| O12 | B12 | O11 | B11 | -2.0(5) |
| O12 | B13 | O13 | B11 | 2.9(5) |
| O13 | B11 | O11 | B12 | 7.1(4) |
| O13 | B11 | O14 | B14 | -113.3(3) |
| O13 | B11 | O16 | B15 | 113.0(3) |
| O13 | B13 | O12 | B12 | 3.2(5) |
| O14 | B11 | O11 | B12 | 124.6(3) |
| O14 | B11 | O13 | B13 | -125.4(3) |
| O14 | B11 | O16 | B15 | -5.4(5) |
| O14 | B14 | O15 | B15 | -2.9(5) |
| O15 | B14 | O14 | B11 | -2.4(5) |
| O15 | B15 | O16 | B11 | 0.9(5) |
| O16 | B11 | O11 | B12 | -113.7(3) |
| O16 | B11 | O13 | B13 | 113.6(3) |
| O16 | B11 | O14 | B14 | 6.2(5) |
| O16 | B15 | O15 | B14 | 3.6(5) |
| O17 | B12 | O11 | B11 | 175.4(3) |
| O17 | B12 | O12 | B13 | 178.8(3) |
| O18 | B13 | O12 | B12 | -178.6(3) |
| O18 | B13 | O13 | B11 | -175.2(3) |
| O19 | B14 | O14 | B11 | 178.9(3) |
| O19 | B14 | O15 | B15 | 175.9(3) |
| O20 | B15 | O15 | B14 | -176.3(3) |
| O20 | B15 | O16 | B11 | -179.1(3) |
| C1^1^ | C1 | N1 | C2 | 179.9(7) |
| C1^1^ | C1 | N1 | C4 | -53.2(9) |
| C3 | C2 | N1 | C1 | -72.4(9) |
| C3 | C2 | N1 | C4 | 158.8(8) |
| C5 | C4 | N1 | C1 | -55.4(10) |
| C5 | C4 | N1 | C2 | 70.3(9) |
| C11^1^ | C11 | N11 | C12 | -55.8(9) |
| C11^1^ | C11 | N11 | C14 | 176.9(7) |
| C13 | C12 | N11 | C11 | -56.1(9) |
| C13 | C12 | N11 | C14 | 70.1(9) |
| C15 | C14 | N11 | C11 | -74.3(9) |
| C15 | C14 | N11 | C12 | 156.3(8) |
| C21 | C31 | N31 | C32 | 48.3(16) |
| C21 | C31 | N31 | C34 | -67.6(13) |
| C23 | C22 | N21 | C21 | -51.9(16) |
| C23 | C22 | N21 | C24 | 176.7(12) |
| C25 | C24 | N21 | C21 | 63.1(15) |
| C25 | C24 | N21 | C22 | -168.0(13) |
| N21 | C21 | C31 | N31 | -164.9(13) |
| C31 | C21 | N21 | C22 | -68.5(14) |
| C31 | C21 | N21 | C24 | 71.0(15) |
| C33 | C32 | N31 | C31 | 72(2) |
| C33 | C32 | N31 | C34 | -165.1(15) |
| C35 | C34 | N31 | C31 | -57.4(16) |
| C35 | C34 | N31 | C32 | 172.8(13) |
| C41^2^ | C41 | N41 | C42 | 70(4) |
| C41^2^ | C41 | N41 | C44 | -55(4) |
| C43 | C42 | N41 | C41 | 63(4) |
| C43 | C42 | N41 | C44 | -165(3) |
| C45 | C44 | N41 | C41 | -74(4) |
| C45 | C44 | N41 | C42 | 157(3) |

––––

^1^1-x,2-y,-z; ^2^1-x,2-y,1-z

**Table 6**: Hydrogen Fractional Atomic Coordinates (×10^4^) and Equivalent Isotropic Displacement Parameters (Å^2^×10^3^) for **MAB2**. *U_eq_* is defined as 1/3 of the trace of the orthogonalised *U_ij_*.

| **Atom** | **x** | **y** | **z** | ***U_eq_*** |
| --- | --- | --- | --- | --- |
| H7 | 8769.93 | 5481.35 | 2812.85 | 54 |
| H8 | 3820.29 | 3223.23 | 1724.08 | 48 |
| H9 | 11205.67 | -885.42 | 2093.72 | 60 |
| H10 | 11154.87 | 4429.76 | -437.78 | 59 |
| H17 | -1209.28 | 10641.3 | 2717.24 | 55 |
| H18 | -1186.53 | 5607.37 | 5392.2 | 54 |
| H19 | 1076.23 | 4047.03 | 2187.91 | 61 |
| H20 | 6069.33 | 6298.4 | 3268.84 | 49 |
| H1A | 6013.91 | 8733.9 | -49.72 | 28 |
| H1B | 4179.88 | 8870.84 | -259.54 | 28 |
| H2A | 5367.05 | 6444.32 | 795.69 | 29 |
| H2B | 4306.73 | 6428.65 | 1556.31 | 29 |
| H3A | 1982.58 | 6824.26 | 842.63 | 62 |
| H3B | 2888.4 | 5287.31 | 771.52 | 62 |
| H3C | 3074.1 | 6717.64 | 105.46 | 62 |
| H4A | 5285.65 | 10133.36 | 1419.9 | 32 |
| H4B | 5071.34 | 8540.04 | 1964.63 | 32 |
| H5A | 7384.21 | 7666.14 | 1317.11 | 57 |
| H5B | 7762.04 | 8904.06 | 1788.56 | 57 |
| H5C | 7649.6 | 9360.02 | 896.96 | 57 |
| H1 | 3345.08 | 8926.57 | 964.44 | 29 |
| H11A | 3956.05 | 9933.55 | 648.98 | 38 |
| H11B | 5767.72 | 10454.17 | 592.9 | 38 |
| H12A | 4740.7 | 7177.85 | -146.16 | 44 |
| H12B | 5033.36 | 6025.8 | 622.2 | 44 |
| H13A | 2725.65 | 6973.45 | 1171.58 | 68 |
| H13B | 2319.14 | 6320.85 | 452.78 | 68 |
| H13C | 2424.12 | 8086.56 | 391.35 | 68 |
| H14A | 4727.85 | 8238.51 | 1751.64 | 41 |
| H14B | 5767.91 | 6761.63 | 1694.33 | 41 |
| H15A | 8098.77 | 8222.89 | 1482.72 | 70 |
| H15B | 7230.01 | 8263.68 | 2283.49 | 70 |
| H15C | 7030.62 | 9648.76 | 1598.95 | 70 |
| H11 | 6682.81 | 8114.62 | 448.87 | 38 |
| H21A | 5597 | 8337.75 | 5033.82 | 50 |
| H21B | 5806.82 | 9767.29 | 4370.41 | 50 |
| H22A | 2377.42 | 8682.97 | 5585.57 | 55 |
| H22B | 1624.34 | 7733.44 | 5041.14 | 55 |
| H23A | 4225.74 | 6431.09 | 5051.71 | 91 |
| H23B | 3104.79 | 6014.45 | 5800.13 | 91 |
| H23C | 4540.59 | 7138.65 | 5765.04 | 91 |
| H24A | 2784.77 | 11234.91 | 4300.31 | 37 |
| H24B | 1789.95 | 10211.04 | 3876.65 | 37 |
| H25A | 3569.03 | 11595.71 | 2972.77 | 66 |
| H25B | 4188.02 | 9892.89 | 3108.03 | 66 |
| H25C | 5044.83 | 11069.35 | 3489.07 | 66 |
| H21 | 3754.29 | 8325.01 | 4178.41 | 32 |
| H31A | 4579.07 | 9747.1 | 5858.89 | 53 |
| H31B | 4287.61 | 11169.62 | 5200.48 | 53 |
| H32A | 8819.32 | 11494.4 | 4970.69 | 54 |
| H32B | 7768.09 | 10772.29 | 4441.51 | 54 |
| H33A | 6525.89 | 12903.49 | 4013.58 | 69 |
| H33B | 8341.84 | 13349.52 | 4050.92 | 69 |
| H33C | 7047.48 | 13638.31 | 4688.09 | 69 |
| H34A | 7898.27 | 8844.1 | 5483.87 | 50 |
| H34B | 8798.86 | 9983.05 | 5872.9 | 50 |
| H35A | 6239.11 | 8113.52 | 6511.24 | 94 |
| H35B | 6597.63 | 9549.05 | 6849.14 | 94 |
| H35C | 7918.55 | 8242.44 | 6864.93 | 94 |
| H31 | 6544.82 | 11586.06 | 5788.53 | 30 |
| H41A | 6628.26 | 9684.18 | 4816.9 | 59 |
| H41B | 5155.48 | 8868.14 | 4563.82 | 59 |
| H42A | 4072.23 | 6688.85 | 6377.62 | 70 |
| H42B | 3121.35 | 8099.71 | 5920.33 | 70 |
| H43A | 3943.35 | 7102.85 | 4801.38 | 93 |
| H43B | 2557.38 | 6242.61 | 5340.65 | 93 |
| H43C | 4346.79 | 5584.49 | 5375.64 | 93 |
| H44A | 5394.87 | 9603.82 | 6438.45 | 70 |
| H44B | 5695.7 | 7894.38 | 6831.4 | 70 |
| H45A | 7976.49 | 9387.02 | 6820.27 | 64 |
| H45B | 8086.62 | 9641.82 | 5923.45 | 64 |
| H45C | 8353.47 | 8003.17 | 6416.54 | 64 |
| H41 | 6378.67 | 7422.86 | 5596.01 | 76 |

**Table 7**: Hydrogen Bond information for **MAB2**.

| **D** | **H** | **A** | **d(D-H)/Å** | **d(H-A)/Å** | **d(D-A)/Å** | **D-H-A/deg** |
| --- | --- | --- | --- | --- | --- | --- |
| O7 | H7 | O14^1^ | 0.84 | 1.87 | 2.709(3) | 174.8 |
| O8 | H8 | O19 | 0.84 | 2.00 | 2.831(3) | 172.8 |
| O9 | H9 | O11^2^ | 0.84 | 1.99 | 2.833(3) | 178.8 |
| O10 | H10 | O6^3^ | 0.84 | 1.95 | 2.786(4) | 175.2 |
| O17 | H17 | O4^4^ | 0.84 | 1.91 | 2.745(3) | 177.6 |
| O18 | H18 | O13^5^ | 0.84 | 1.92 | 2.763(3) | 175.8 |
| O19 | H19 | O1^6^ | 0.84 | 1.85 | 2.692(3) | 176.5 |
| O20 | H20 | O7 | 0.84 | 1.97 | 2.806(3) | 174.4 |
| N1 | H1 | O9^4^ | 1.00 | 1.99 | 2.892(6) | 149.3 |
| N11 | H11 | O5^3^ | 1.00 | 2.45 | 3.351(6) | 149.1 |
| N11 | H11 | O10^3^ | 1.00 | 2.14 | 2.962(6) | 138.6 |
| N41 | H41 | O18^1^ | 1.00 | 2.02 | 2.99(2) | 163.1 |

––––

^1^1+x,+y,+z; ^2^1+x,-1+y,+z; ^3^2-x,1-y,-z; ^4^-1+x,1+y,+z; ^5^-x,1-y,1-z; ^6^-1+x,+y,+z

**Table 8**: Atomic Occupancies for all atoms that are not fully occupied in **MAB2**.

| **Atom** | **Occupancy** |
| --- | --- |
| C1 | 0.473(5) |
| H1A | 0.473(5) |
| H1B | 0.473(5) |
| C2 | 0.473(5) |
| H2A | 0.473(5) |
| H2B | 0.473(5) |
| C3 | 0.473(5) |
| H3A | 0.473(5) |
| H3B | 0.473(5) |
| H3C | 0.473(5) |
| C4 | 0.473(5) |
| H4A | 0.473(5) |
| H4B | 0.473(5) |
| C5 | 0.473(5) |
| H5A | 0.473(5) |
| H5B | 0.473(5) |
| H5C | 0.473(5) |
| N1 | 0.473(5) |
| H1 | 0.473(5) |
| C11 | 0.527(5) |
| H11A | 0.527(5) |
| H11B | 0.527(5) |
| C12 | 0.527(5) |
| H12A | 0.527(5) |
| H12B | 0.527(5) |
| C13 | 0.527(5) |
| H13A | 0.527(5) |
| H13B | 0.527(5) |
| H13C | 0.527(5) |
| C14 | 0.527(5) |
| H14A | 0.527(5) |
| H14B | 0.527(5) |
| C15 | 0.527(5) |
| H15A | 0.527(5) |
| H15B | 0.527(5) |
| H15C | 0.527(5) |
| N11 | 0.527(5) |
| H11 | 0.527(5) |
| C21 | 0.4335(19) |
| H21A | 0.4335(19) |
| H21B | 0.4335(19) |
| C22 | 0.4335(19) |
| H22A | 0.4335(19) |
| H22B | 0.4335(19) |
| C23 | 0.4335(19) |
| H23A | 0.4335(19) |
| H23B | 0.4335(19) |
| H23C | 0.4335(19) |
| C24 | 0.4335(19) |
| H24A | 0.4335(19) |
| H24B | 0.4335(19) |
| C25 | 0.4335(19) |
| H25A | 0.4335(19) |
| H25B | 0.4335(19) |
| H25C | 0.4335(19) |
| N21 | 0.4335(19) |
| H21 | 0.4335(19) |
| C31 | 0.4335(19) |
| H31A | 0.4335(19) |
| H31B | 0.4335(19) |
| C32 | 0.4335(19) |
| H32A | 0.4335(19) |
| H32B | 0.4335(19) |
| C33 | 0.4335(19) |
| H33A | 0.4335(19) |
| H33B | 0.4335(19) |
| H33C | 0.4335(19) |
| C34 | 0.4335(19) |
| H34A | 0.4335(19) |
| H34B | 0.4335(19) |
| C35 | 0.4335(19) |
| H35A | 0.4335(19) |
| H35B | 0.4335(19) |
| H35C | 0.4335(19) |
| N31 | 0.4335(19) |
| H31 | 0.4335(19) |
| C41 | 0.133(4) |
| H41A | 0.133(4) |
| H41B | 0.133(4) |
| C42 | 0.133(4) |
| H42A | 0.133(4) |
| H42B | 0.133(4) |
| C43 | 0.133(4) |
| H43A | 0.133(4) |
| H43B | 0.133(4) |
| H43C | 0.133(4) |
| C44 | 0.133(4) |
| H44A | 0.133(4) |
| H44B | 0.133(4) |
| C45 | 0.133(4) |
| H45A | 0.133(4) |
| H45B | 0.133(4) |
| H45C | 0.133(4) |
| N41 | 0.133(4) |
| H41 | 0.133(4) |
