# Supplementary material for: Pentaborate(1-) Salts and a Tetraborate(2-) Salt Derived from C2- or C3-Linked Bis(alkylammonium) Dications: Synthesis, Characterization, and Structural (XRD) Studies
Source: Molecules. 2019 Dec 23;25(1):53. doi: 10.3390/molecules25010053 (PMC6982793; doi:10.3390/molecules25010053)

checkCIF/PLATON report

Structure factors have been supplied for datablock(s) MAB2

THIS REPORT IS FOR GUIDANCE ONLY. IF USED AS PART OF A REVIEW PROCEDURE FOR PUBLICATION, IT SHOULD NOT REPLACE THE EXPERTISE OF AN EXPERIENCED CRYSTALLOGRAPHIC REFEREE.

No syntax errors found. CIF dictionary Interpreting this report

Datablock: MAB2

```
Bond precision:  O- B = 0.0043 A         Wavelength=0.71075

Cell:           a=8.3998(5)       b=9.1406(7)      c=18.2066(13)
                alpha=78.439(6)   beta=86.810(5)   gamma=88.118(6)
Temperature:    100 K

                 Calculated                 Reported
Volume           1367.07(17)                1367.07(17)
Space group      P -1                       P -1
Hall group       -P 1                       -P 1
Moiety formula   2(B5 H4 O10), C10 H26 N2   2(B5 H4 O10), 2(C5 H13 N)
Sum formula      C10 H34 B10 N2 O20         C10 H34 B10 N2 O20
Mr               610.49                     610.49
Dx,g cm-3        1.483                      1.483
Z                2                          2
Mu (mm-1)        0.130                      0.130
F000             636.0                      636.0
F000'            636.45
h,k,lmax         10,11,23                   10,11,23
Nref             6265                       6146
Tmin,Tmax        0.994,0.999                0.727,1.000
Tmin'            0.987

Correction method= # Reported T Limits: Tmin=0.727 Tmax=1.000
AbsCorr = MULTI-SCAN

Data completeness= 0.981          Theta(max)= 27.486

R(reflections)= 0.0748( 2997)     wR2(reflections)= 0.1850( 6146)

S = 1.005                 Npar= 561
```

The following ALERTS were generated. Each ALERT has the format
test-name_ALERT_alert-type_alert-level.
Click on the hyperlinks for more details of the test.

Alert level C

```
PLAT026_ALERT_3_C Ratio Observed / Unique Reflections (too) Low ..        49% Check
PLAT250_ALERT_2_C Large U3/U1 Ratio for Average U(i,j) Tensor ....        2.7 Note
PLAT250_ALERT_2_C Large U3/U1 Ratio for Average U(i,j) Tensor ....        2.7 Note
PLAT250_ALERT_2_C Large U3/U1 Ratio for Average U(i,j) Tensor ....        2.7 Note
PLAT430_ALERT_2_C Short Inter D...A Contact  O2       ..O15      .       2.87 Ang.
                                                    x,y,z  =      1_555 Check
PLAT906_ALERT_3_C Large K Value in the Analysis of Variance ......     14.957 Check
PLAT906_ALERT_3_C Large K Value in the Analysis of Variance ......      3.420 Check
PLAT911_ALERT_3_C Missing FCF Refl Between Thmin & STh/L=    0.600         77 Report
```

Alert level G

```
PLAT002_ALERT_2_G Number of Distance or Angle Restraints on AtSite         30 Note
PLAT003_ALERT_2_G Number of Uiso or Uij Restrained non-H Atoms ...         18 Report
PLAT007_ALERT_5_G Number of Unrefined Donor-H Atoms ..............         13 Report
PLAT042_ALERT_1_G Calc. and Reported MoietyFormula Strings  Differ     Please Check
PLAT172_ALERT_4_G The CIF-Embedded .res File Contains DFIX Records          1 Report
PLAT175_ALERT_4_G The CIF-Embedded .res File Contains SAME Records          4 Report
PLAT176_ALERT_4_G The CIF-Embedded .res File Contains SADI Records          1 Report
PLAT178_ALERT_4_G The CIF-Embedded .res File Contains SIMU Records          1 Report
PLAT187_ALERT_4_G The CIF-Embedded .res File Contains RIGU Records          1 Report
PLAT302_ALERT_4_G Anion/Solvent/Minor-Residue Disorder (Resd  3  )       100% Note
PLAT302_ALERT_4_G Anion/Solvent/Minor-Residue Disorder (Resd  4  )       100% Note
PLAT302_ALERT_4_G Anion/Solvent/Minor-Residue Disorder (Resd  5  )       100% Note
PLAT302_ALERT_4_G Anion/Solvent/Minor-Residue Disorder (Resd  6  )       100% Note
PLAT304_ALERT_4_G Non-Integer Number of Atoms in ...... Resd  3         20.03 Check
PLAT304_ALERT_4_G Non-Integer Number of Atoms in ...... Resd  4         17.97 Check
PLAT304_ALERT_4_G Non-Integer Number of Atoms in ...... Resd  5         16.47 Check
PLAT304_ALERT_4_G Non-Integer Number of Atoms in ...... Resd  6          5.05 Check
PLAT432_ALERT_2_G Short Inter X...Y Contact  O12      ..C41              2.96 Ang.
                                                 -1+x,y,z  =      1_455 Check
PLAT789_ALERT_4_G Atoms with Negative _atom_site_disorder_group  #         38 Check
PLAT811_ALERT_5_G No ADDSYM Analysis: Too Many Excluded Atoms ....          ! Info
PLAT860_ALERT_3_G Number of Least-Squares Restraints ............         388 Note
PLAT910_ALERT_3_G Missing # of FCF Reflection(s) Below Theta(Min).          1 Note
PLAT912_ALERT_4_G Missing # of FCF Reflections Above STh/L=  0.600         41 Note
```

0 **ALERT level A** = Most likely a serious problem - resolve or explain
0 **ALERT level B** = A potentially serious problem, consider carefully
8 **ALERT level C** = Check. Ensure it is not caused by an omission or oversight
23 **ALERT level G** = General information/check it is not something unexpected

1 ALERT type 1 CIF construction/syntax error, inconsistent or missing data
7 ALERT type 2 Indicator that the structure model may be wrong or deficient
6 ALERT type 3 Indicator that the structure quality may be low
15 ALERT type 4 Improvement, methodology, query or suggestion
2 ALERT type 5 Informative message, check

It is advisable to attempt to resolve as many as possible of the alerts in all categories. Often the minor alerts point to easily fixed oversights, errors and omissions in your CIF or refinement strategy, so attention to these fine details can be worthwhile. In order to resolve some of the more serious problems it may be necessary to carry out additional measurements or structure refinements. However, the purpose of your study may justify the reported deviations and the more serious of these should normally be commented upon in the discussion or experimental section of a paper or in the "special_details" fields of the CIF. checkCIF was carefully designed to identify outliers and unusual parameters, but every test has its limitations and alerts that are not important in a particular case may appear. Conversely, the absence of alerts does not guarantee there are no aspects of the results needing attention. It is up to the individual to critically assess their own results and, if necessary, seek expert advice.

Publication of your CIF in IUCr journals

A basic structural check has been run on your CIF. These basic checks will be run on all CIFs submitted for publication in IUCr journals (*Acta Crystallographica*, *Journal of Applied Crystallography*, *Journal of Synchrotron Radiation*); however, if you intend to submit to *Acta Crystallographica Section C* or *E* or *IUCrData*, you should make sure that full publication checks are run on the final version of your CIF prior to submission.

Publication of your CIF in other journals

Please refer to the *Notes for Authors* of the relevant journal for any special instructions relating to CIF submission.

PLATON version of 07/08/2019; check.def file version of 30/07/2019

Datablock MAB2 - ellipsoid plot

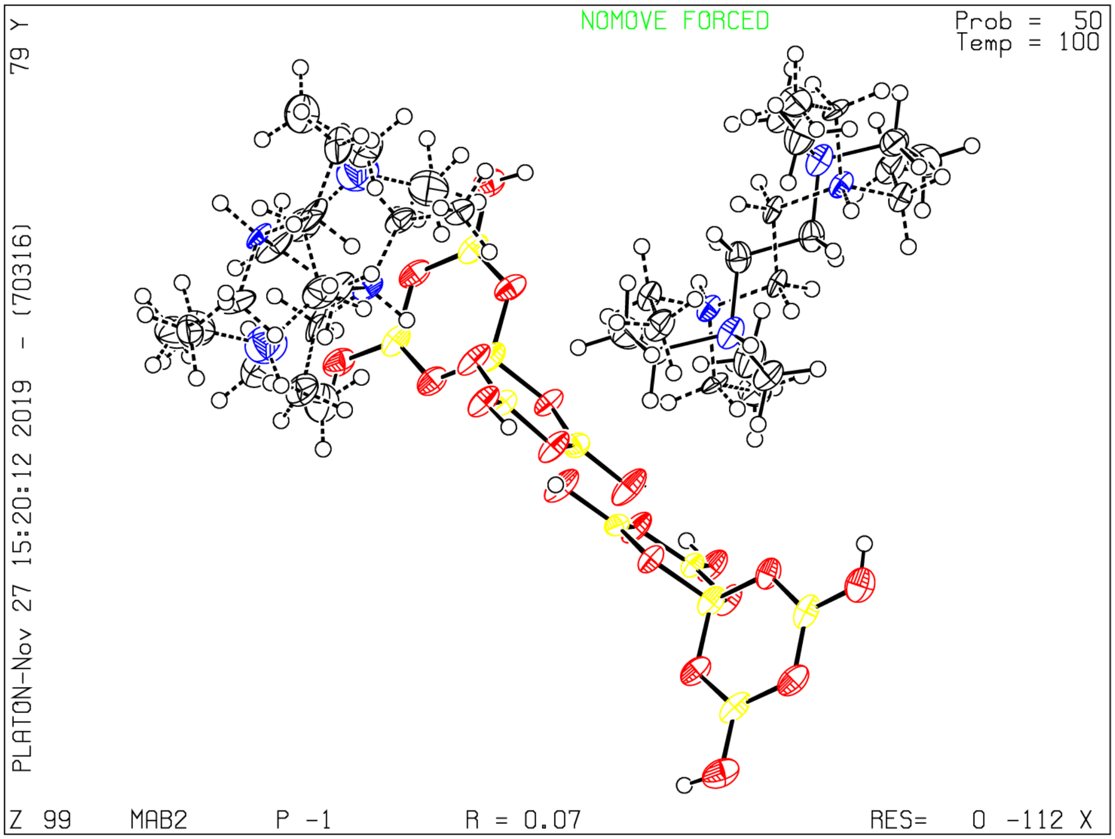

Supplement: Supplementary file 1 [file molecules-25-00053-s001.zip › MAB2_checkcif.pdf]
