# Supplementary material for: Pentaborate(1-) Salts and a Tetraborate(2-) Salt Derived from C2- or C3-Linked Bis(alkylammonium) Dications: Synthesis, Characterization, and Structural (XRD) Studies
Source: Molecules. 2019 Dec 23;25(1):53. doi: 10.3390/molecules25010053 (PMC6982793; doi:10.3390/molecules25010053)
Supplement: Supplementary file 1 [file molecules-25-00053-s001.zip › MAB3.docx]

**MAB3**


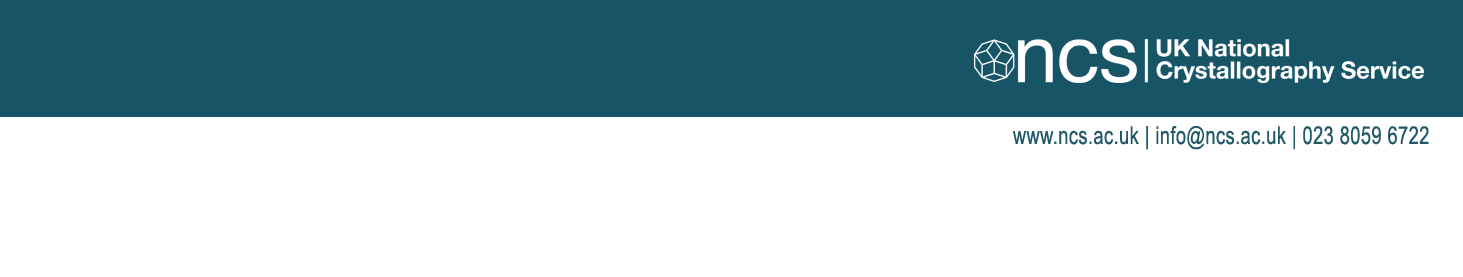


Submitted by: **None**

None

Solved by: **None**

Sample ID: **MAB3**

***R_1_*=3.63%**

Crystal Data and Experimental


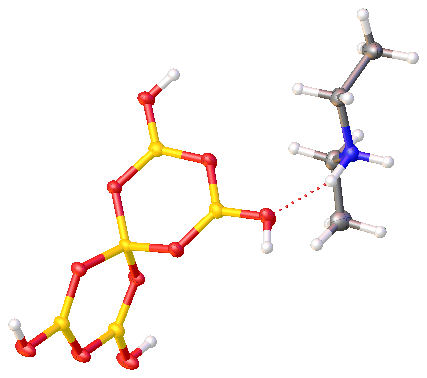


**Experimental.** Single colourless slab crystals of **MAB3** recrystallised from water. A suitable crystal with dimensions 0.150 × 0.100 × 0.050 mm^3^ was selected and mounted on a Rigaku FRE+ equipped with HF Varimax confocal mirrors and an AFC12 goniometer and HG Saturn 724+ detector diffractometer. The crystal was kept at a steady *T* = 100(2) K during data collection. The structure was solved with the **ShelXT** 2018/2 (Sheldrick, 2018) solution program using dual methods and by using **Olex2** (Dolomanov et al., 2009) as the graphical interface. The model was refined with **ShelXL** 2018/3 (Sheldrick, 2015) using full matrix least squares minimisation on ***F*^2^**.

**Crystal Data.** C_4_H_16_B_5_NO_10_, *M_r_* = 292.23, triclinic, *P*-1 (No. 2), a = 8.3793(3) Å, b = 8.8187(4) Å, c = 10.0877(4) Å, *α* = 80.961(3)^°^, *β* = 76.057(3)^°^, *γ* = 68.269(4)^°^, *V* = 670.14(5) Å^3^, *T* = 100(2) K, *Z* = 2, *Z'* = 1, *μ*(Mo K*_α_*) = 0.129, 5980 reflections measured, 3032 unique (*R_int_* = 0.0144) which were used in all calculations. The final *wR_2_* was 0.1024 (all data) and *R_1_* was 0.0363 (I > 2(I)).

| **Compound** | **MAB3** |
| --- | --- |
|  |  |
| Formula | C_4_H_16_B_5_NO_10_ |
| *D_calc._*/ g cm^-3^ | 1.448 |
| *μ*/mm^-1^ | 0.129 |
| Formula Weight | 292.23 |
| Colour | colourless |
| Shape | slab |
| Size/mm^3^ | 0.150×0.100×0.050 |
| *T*/K | 100(2) |
| Crystal System | triclinic |
| Space Group | *P*-1 |
| *a*/Å | 8.3793(3) |
| *b*/Å | 8.8187(4) |
| *c*/Å | 10.0877(4) |
| *α*/^°^ | 80.961(3) |
| *β*/^°^ | 76.057(3) |
| *γ*/^°^ | 68.269(4) |
| V/Å^3^ | 670.14(5) |
| *Z* | 2 |
| *Z'* | 1 |
| Wavelength/Å | 0.71075 |
| Radiation type | Mo K*_α_* |
| *Θ_min_*/^°^ | 2.493 |
| *Θ_max_*/^°^ | 27.480 |
| Measured Refl's. | 5980 |
| Ind't Refl's | 3032 |
| Refl's with I > 2(I) | 2764 |
| *R_int_* | 0.0144 |
| Parameters | 207 |
| Restraints | 0 |
| Largest Peak | 0.629 |
| Deepest Hole | -0.235 |
| GooF | 1.037 |
| *wR_2_* (all data) | 0.1024 |
| *wR_2_* | 0.0994 |
| *R_1_* (all data) | 0.0396 |
| *R_1_* | 0.0363 |

**Table 1**: Fractional Atomic Coordinates (×10^4^) and Equivalent Isotropic Displacement Parameters (Å^2^×10^3^) for **MAB3**. *U_eq_* is defined as 1/3 of the trace of the orthogonalised *U_ij_*.

| **Atom** | **x** | **y** | **z** | ***U_eq_*** |
| --- | --- | --- | --- | --- |
| B1 | 2717.4(16) | 6020.2(14) | 7850.1(12) | 13.8(2) |
| B2 | 3766.5(17) | 3703.9(15) | 6373.6(13) | 16.6(2) |
| B3 | 1613.9(16) | 3713.6(15) | 8435.3(13) | 15.8(2) |
| B4 | 4677.5(16) | 6976.1(15) | 8683.0(13) | 16.2(2) |
| B5 | 2598.9(16) | 8869.1(15) | 7423.2(13) | 15.4(2) |
| O1 | 3694.6(10) | 5206.4(9) | 6588.5(8) | 15.55(18) |
| O2 | 2795.9(11) | 2904.2(9) | 7338.7(8) | 18.72(19) |
| O3 | 1437.6(10) | 5272.7(9) | 8606.0(8) | 14.47(18) |
| O4 | 3945.4(10) | 5828.4(9) | 8746.2(8) | 15.96(18) |
| O5 | 4082.6(11) | 8473.8(9) | 7971.9(8) | 17.60(19) |
| O6 | 1815.5(10) | 7742.8(9) | 7495.1(8) | 14.88(18) |
| O7 | 4725.4(12) | 2915.6(10) | 5250.2(9) | 23.5(2) |
| O8 | 656.5(12) | 2897.8(10) | 9303.5(9) | 21.6(2) |
| O9 | 6027.0(11) | 6723.5(10) | 9317.6(9) | 20.4(2) |
| O10 | 1887.1(11) | 10373.2(10) | 6836.2(9) | 19.09(19) |
| C1 | 6932.4(16) | 10286.8(15) | 8411.7(14) | 24.2(3) |
| C2 | 7514.1(18) | 11701.5(16) | 7757.3(17) | 32.9(3) |
| C3 | 8039.8(16) | 8647.0(15) | 6325.3(12) | 22.3(3) |
| C4 | 9024.8(18) | 6935.8(16) | 5880.5(14) | 28.0(3) |
| N1 | 8073.6(13) | 8705.9(12) | 7791.1(10) | 18.7(2) |

**Table 2**: Anisotropic Displacement Parameters (×10^4^) for **MAB3**. The anisotropic displacement factor exponent takes the form: *-2π^2^[h^2^a*^2^ × U_11_+ ... +2hka* × b* × U_12_]*

| **Atom** | ***U_11_*** | ***U_22_*** | ***U_33_*** | ***U_23_*** | ***U_13_*** | ***U_12_*** |
| --- | --- | --- | --- | --- | --- | --- |
| B1 | 14.8(5) | 12.8(5) | 14.3(6) | -1.1(4) | -0.9(4) | -6.7(4) |
| B2 | 18.0(6) | 15.8(6) | 16.4(6) | -1.2(4) | -1.0(5) | -7.9(5) |
| B3 | 16.7(6) | 15.1(5) | 16.2(6) | -1.2(4) | -1.4(4) | -7.5(5) |
| B4 | 16.7(6) | 16.3(6) | 16.1(6) | -0.9(4) | -1.4(4) | -7.7(5) |
| B5 | 16.6(6) | 14.6(5) | 14.8(6) | -2.4(4) | -0.2(4) | -6.3(5) |
| O1 | 17.3(4) | 14.6(4) | 14.9(4) | -2.1(3) | 1.5(3) | -8.3(3) |
| O2 | 23.0(4) | 14.9(4) | 18.2(4) | -3.9(3) | 4.2(3) | -10.6(3) |
| O3 | 15.2(4) | 13.6(4) | 14.8(4) | -2.4(3) | 1.0(3) | -7.2(3) |
| O4 | 17.6(4) | 14.2(4) | 18.0(4) | 1.5(3) | -5.2(3) | -7.7(3) |
| O5 | 19.6(4) | 15.5(4) | 20.8(4) | 1.6(3) | -6.1(3) | -9.5(3) |
| O6 | 14.8(4) | 12.6(4) | 17.9(4) | -0.7(3) | -3.2(3) | -5.9(3) |
| O7 | 30.1(5) | 19.1(4) | 21.1(4) | -6.8(3) | 8.5(4) | -14.5(4) |
| O8 | 25.9(5) | 17.9(4) | 21.0(4) | -5.4(3) | 6.5(3) | -13.2(4) |
| O9 | 22.7(4) | 19.8(4) | 23.4(4) | 5.2(3) | -9.8(3) | -12.1(3) |
| O10 | 21.5(4) | 13.7(4) | 24.1(4) | 0.4(3) | -7.0(3) | -7.6(3) |
| C1 | 18.6(6) | 23.1(6) | 28.8(7) | -6.4(5) | 0.5(5) | -6.4(5) |
| C2 | 23.9(6) | 21.4(6) | 50.6(9) | -6.9(6) | 0.3(6) | -7.8(5) |
| C3 | 21.2(6) | 25.3(6) | 19.6(6) | 0.1(5) | -5.1(4) | -7.2(5) |
| C4 | 31.8(7) | 26.9(6) | 26.6(7) | -4.1(5) | -10.6(5) | -8.0(5) |
| N1 | 17.1(5) | 18.8(5) | 20.6(5) | -0.2(4) | -3.4(4) | -7.5(4) |

**Table 3**: Bond Lengths in Å for **MAB3**.

| **Atom** | **Atom** | **Length/Å** |
| --- | --- | --- |
| B1 | O1 | 1.4679(14) |
| B1 | O3 | 1.4620(14) |
| B1 | O4 | 1.4751(14) |
| B1 | O6 | 1.4548(14) |
| B2 | O1 | 1.3533(14) |
| B2 | O2 | 1.3907(15) |
| B2 | O7 | 1.3447(15) |
| B3 | O2 | 1.3823(15) |
| B3 | O3 | 1.3614(14) |
| B3 | O8 | 1.3487(15) |
| B4 | O4 | 1.3511(14) |
| B4 | O5 | 1.3765(15) |
| B4 | O9 | 1.3622(15) |
| B5 | O5 | 1.3869(15) |
| B5 | O6 | 1.3638(14) |
| B5 | O10 | 1.3429(15) |
| C1 | C2 | 1.5054(19) |
| C1 | N1 | 1.4995(15) |
| C3 | C4 | 1.5072(18) |
| C3 | N1 | 1.4951(15) |

**Table 4**: Bond Angles in ^°^ for **MAB3**.

| **Atom** | **Atom** | **Atom** | **Angle/^°^** |
| --- | --- | --- | --- |
| O1 | B1 | O4 | 109.32(9) |
| O3 | B1 | O1 | 110.89(9) |
| O3 | B1 | O4 | 107.40(9) |
| O6 | B1 | O1 | 108.97(9) |
| O6 | B1 | O3 | 109.50(9) |
| O6 | B1 | O4 | 110.75(9) |
| O1 | B2 | O2 | 119.81(10) |
| O7 | B2 | O1 | 123.59(10) |
| O7 | B2 | O2 | 116.60(10) |
| O3 | B3 | O2 | 120.42(10) |
| O8 | B3 | O2 | 117.19(10) |
| O8 | B3 | O3 | 122.39(11) |
| O4 | B4 | O5 | 121.23(10) |
| O4 | B4 | O9 | 122.24(10) |
| O9 | B4 | O5 | 116.53(10) |
| O6 | B5 | O5 | 120.44(10) |
| O10 | B5 | O5 | 120.79(10) |
| O10 | B5 | O6 | 118.76(10) |
| B2 | O1 | B1 | 123.16(9) |
| B3 | O2 | B2 | 119.97(9) |
| B3 | O3 | B1 | 121.88(9) |
| B4 | O4 | B1 | 120.55(9) |
| B4 | O5 | B5 | 119.23(9) |
| B5 | O6 | B1 | 120.72(9) |
| N1 | C1 | C2 | 112.77(11) |
| N1 | C3 | C4 | 110.30(10) |
| C3 | N1 | C1 | 115.35(10) |

**Table 5**: Torsion Angles in ^°^ for **MAB3**.

| **Atom** | **Atom** | **Atom** | **Atom** | **Angle/^°^** |
| --- | --- | --- | --- | --- |
| O1 | B1 | O3 | B3 | 22.75(14) |
| O1 | B1 | O4 | B4 | 92.18(11) |
| O1 | B1 | O6 | B5 | -91.05(11) |
| O1 | B2 | O2 | B3 | 7.24(17) |
| O2 | B2 | O1 | B1 | 4.10(17) |
| O2 | B3 | O3 | B1 | -13.85(16) |
| O3 | B1 | O1 | B2 | -17.99(14) |
| O3 | B1 | O4 | B4 | -147.42(10) |
| O3 | B1 | O6 | B5 | 147.50(9) |
| O3 | B3 | O2 | B2 | -2.40(16) |
| O4 | B1 | O1 | B2 | 100.24(11) |
| O4 | B1 | O3 | B3 | -96.64(11) |
| O4 | B1 | O6 | B5 | 29.24(13) |
| O4 | B4 | O5 | B5 | 6.30(16) |
| O5 | B4 | O4 | B1 | 11.50(16) |
| O5 | B5 | O6 | B1 | -14.33(15) |
| O6 | B1 | O1 | B2 | -138.60(10) |
| O6 | B1 | O3 | B3 | 143.04(10) |
| O6 | B1 | O4 | B4 | -27.89(14) |
| O6 | B5 | O5 | B4 | -4.99(16) |
| O7 | B2 | O1 | B1 | -176.70(10) |
| O7 | B2 | O2 | B3 | -172.02(10) |
| O8 | B3 | O2 | B2 | 177.17(10) |
| O8 | B3 | O3 | B1 | 166.60(10) |
| O9 | B4 | O4 | B1 | -168.80(10) |
| O9 | B4 | O5 | B5 | -173.42(10) |
| O10 | B5 | O5 | B4 | 173.70(10) |
| O10 | B5 | O6 | B1 | 166.96(10) |
| C2 | C1 | N1 | C3 | -64.94(14) |
| C4 | C3 | N1 | C1 | -171.96(10) |

**Table 6**: Hydrogen Fractional Atomic Coordinates (×10^4^) and Equivalent Isotropic Displacement Parameters (Å^2^×10^3^) for **MAB3**. *U_eq_* is defined as 1/3 of the trace of the orthogonalised *U_ij_*.

| **Atom** | **x** | **y** | **z** | ***U_eq_*** |
| --- | --- | --- | --- | --- |
| H1A | 6944.59 | 10160.31 | 9402.28 | 29 |
| H1B | 5711.27 | 10534.02 | 8315.17 | 29 |
| H2A | 8714.87 | 11473.79 | 7864.41 | 49 |
| H2B | 6730.44 | 12699.72 | 8200.82 | 49 |
| H2C | 7475.52 | 11851.93 | 6780.64 | 49 |
| H3A | 8581.76 | 9411.44 | 5742.23 | 27 |
| H3B | 6810.35 | 8996.68 | 6211.6 | 27 |
| H4A | 10241.23 | 6591.8 | 5994.09 | 42 |
| H4B | 9006.39 | 6917.22 | 4915.62 | 42 |
| H4C | 8467.42 | 6185.87 | 6442.64 | 42 |
| H1C | 9190(20) | 8453(18) | 7871(15) | 21(4) |
| H8 | -100(30) | 3540(20) | 9970(20) | 45(5) |
| H1D | 7690(20) | 7860(20) | 8277(18) | 32(4) |
| H10 | 2360(30) | 11070(20) | 6931(19) | 45(5) |
| H7 | 5290(30) | 3520(30) | 4710(20) | 52(6) |
| H9 | 6230(20) | 5800(20) | 9810(20) | 41(5) |

**Table 7**: Hydrogen Bond information for **MAB3**.

| **D** | **H** | **A** | **d(D-H)/Å** | **d(H-A)/Å** | **d(D-A)/Å** | **D-H-A/deg** |
| --- | --- | --- | --- | --- | --- | --- |
| N1 | H1C | O6^1^ | 0.895(16) | 2.010(16) | 2.8807(13) | 163.7(14) |
| O8 | H8 | O3^2^ | 0.90(2) | 1.80(2) | 2.7053(12) | 174.2(18) |
| N1 | H1D | O9 | 0.944(18) | 2.009(18) | 2.9103(13) | 159.0(15) |
| O10 | H10 | O2^3^ | 0.87(2) | 1.91(2) | 2.7602(11) | 165.4(19) |
| O7 | H7 | O1^4^ | 0.88(2) | 1.85(2) | 2.7223(12) | 172.1(19) |
| O9 | H9 | O4^5^ | 0.87(2) | 1.90(2) | 2.7353(12) | 162.1(18) |

––––

^1^1+x,+y,+z; ^2^-x,1-y,2-z; ^3^+x,1+y,+z; ^4^1-x,1-y,1-z; ^5^1-x,1-y,2-z
