# Supplementary material for: Pentaborate(1-) Salts and a Tetraborate(2-) Salt Derived from C2- or C3-Linked Bis(alkylammonium) Dications: Synthesis, Characterization, and Structural (XRD) Studies
Source: Molecules. 2019 Dec 23;25(1):53. doi: 10.3390/molecules25010053 (PMC6982793; doi:10.3390/molecules25010053)

checkCIF/PLATON report

Structure factors have been supplied for datablock(s) MAB3

THIS REPORT IS FOR GUIDANCE ONLY. IF USED AS PART OF A REVIEW PROCEDURE FOR PUBLICATION, IT SHOULD NOT REPLACE THE EXPERTISE OF AN EXPERIENCED CRYSTALLOGRAPHIC REFEREE.

No syntax errors found.   CIF dictionary   Interpreting this report

Datablock: MAB3

```
Bond precision:  C-C = 0.0019 A          Wavelength=0.71075

Cell:           a=8.3793(3)       b=8.8187(4)      c=10.0877(4)
                alpha=80.961(3)   beta=76.057(3)   gamma=68.269(4)
Temperature:    100 K

                Calculated                 Reported
Volume          670.14(5)                  670.14(5)
Space group     P -1                       P -1
Hall group      -P 1                       -P 1
Moiety formula  B5 H4 O10, C4 H12 N        B5 H4 O10, C4 H12 N
Sum formula     C4 H16 B5 N O10            C4 H16 B5 N O10
Mr              292.23                     292.23
Dx,g cm-3       1.448                      1.448
Z               2                          2
Mu (mm-1)       0.129                      0.129
F000            304.0                      304.0
F000'           304.22
h,k,lmax        10,11,13                   10,11,13
Nref            3071                       3032
Tmin,Tmax       0.985,0.994                0.775,1.000
Tmin'           0.981

Correction method= # Reported T Limits: Tmin=0.775 Tmax=1.000
AbsCorr = MULTI-SCAN

Data completeness= 0.987          Theta(max)= 27.480

R(reflections)= 0.0363( 2764)     wR2(reflections)= 0.1024( 3032)

S = 1.037                   Npar= 207
```

The following ALERTS were generated. Each ALERT has the format
test-name_ALERT_alert-type_alert-level.
Click on the hyperlinks for more details of the test.

Alert level C

```
DIFMX02_ALERT_1_C  The maximum difference density is > 0.1*ZMAX*0.75
            The relevant atom site should be identified.
PLAT094_ALERT_2_C Ratio of Maximum / Minimum Residual Density ....       2.68 Report
PLAT097_ALERT_2_C Large Reported Max.  (Positive) Residual Density       0.63 eA-3
PLAT911_ALERT_3_C Missing FCF Refl Between Thmin & STh/L=    0.600         30 Report
```

Alert level G

```
PLAT910_ALERT_3_G Missing # of FCF Reflection(s) Below Theta(Min).          1 Note
PLAT912_ALERT_4_G Missing # of FCF Reflections Above STh/L=  0.600          9 Note
PLAT913_ALERT_3_G Missing # of Very Strong Reflections in FCF ....          2 Note
PLAT978_ALERT_2_G Number C-C Bonds with Positive Residual Density.          1 Info
```

```
   0 ALERT level A = Most likely a serious problem - resolve or explain
   0 ALERT level B = A potentially serious problem, consider carefully
   4 ALERT level C = Check. Ensure it is not caused by an omission or oversight
   4 ALERT level G = General information/check it is not something unexpected

   1 ALERT type 1 CIF construction/syntax error, inconsistent or missing data
   3 ALERT type 2 Indicator that the structure model may be wrong or deficient
   3 ALERT type 3 Indicator that the structure quality may be low
   1 ALERT type 4 Improvement, methodology, query or suggestion
   0 ALERT type 5 Informative message, check
```

It is advisable to attempt to resolve as many as possible of the alerts in all categories. Often the minor alerts point to easily fixed oversights, errors and omissions in your CIF or refinement strategy, so attention to these fine details can be worthwhile. In order to resolve some of the more serious problems it may be necessary to carry out additional measurements or structure refinements. However, the purpose of your study may justify the reported deviations and the more serious of these should normally be commented upon in the discussion or experimental section of a paper or in the "special_details" fields of the CIF. checkCIF was carefully designed to identify outliers and unusual parameters, but every test has its limitations and alerts that are not important in a particular case may appear. Conversely, the absence of alerts does not guarantee there are no aspects of the results needing attention. It is up to the individual to critically assess their own results and, if necessary, seek expert advice.

Publication of your CIF in IUCr journals

A basic structural check has been run on your CIF. These basic checks will be run on all CIFs submitted for publication in IUCr journals (*Acta Crystallographica*, *Journal of Applied Crystallography*, *Journal of Synchrotron Radiation*); however, if you intend to submit to *Acta Crystallographica Section C* or *E* or *IUCrData*, you should make sure that full publication checks are run on the final version of your CIF prior to submission.

Publication of your CIF in other journals

Please refer to the *Notes for Authors* of the relevant journal for any special instructions relating to CIF submission.

PLATON version of 07/08/2019; check.def file version of 30/07/2019

Datablock MAB3 - ellipsoid plot

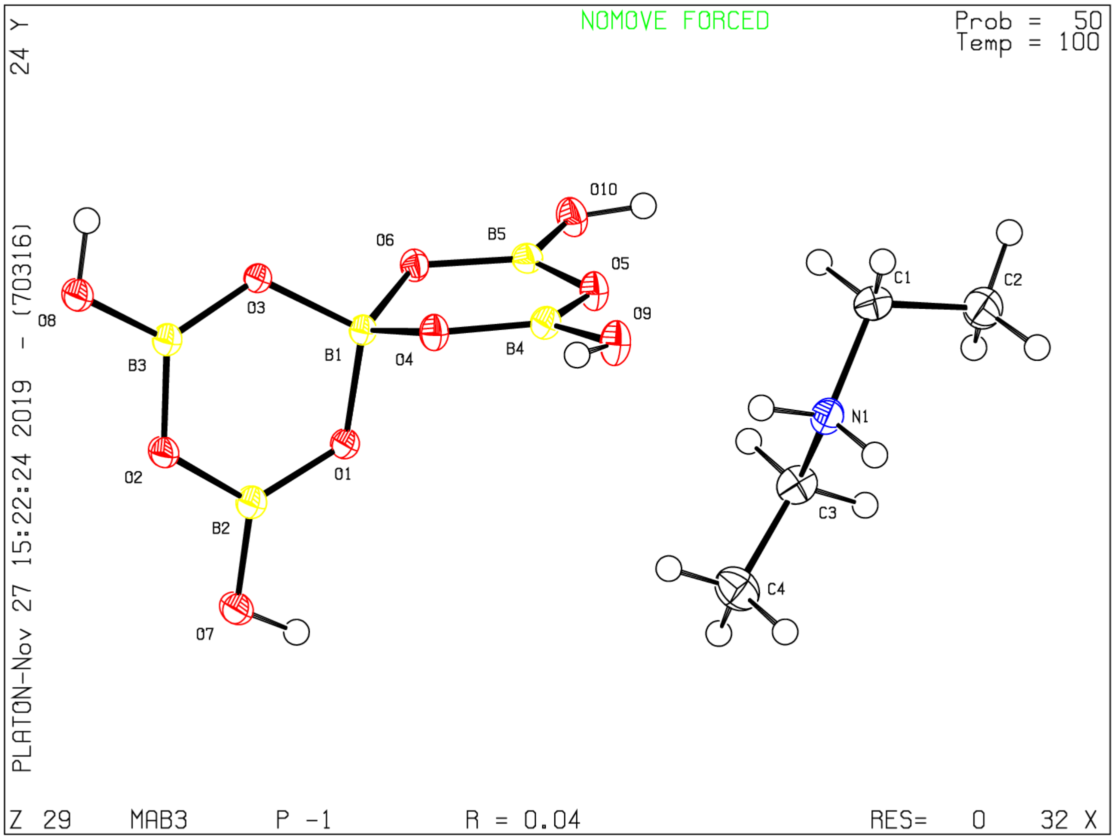

Supplement: Supplementary file 1 [file molecules-25-00053-s001.zip › MAB3_checkcif.pdf]
