# Supplementary material for: Pentaborate(1-) Salts and a Tetraborate(2-) Salt Derived from C2- or C3-Linked Bis(alkylammonium) Dications: Synthesis, Characterization, and Structural (XRD) Studies
Source: Molecules. 2019 Dec 23;25(1):53. doi: 10.3390/molecules25010053 (PMC6982793; doi:10.3390/molecules25010053)
Supplement: Supplementary file 1 [file molecules-25-00053-s001.zip › MAB7.docx]

**MAB7**


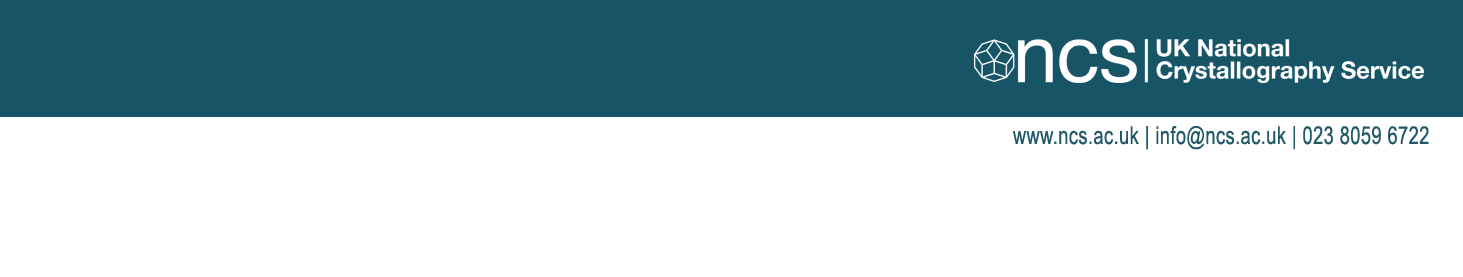


Submitted by: **None**

None

Solved by: **None**

Sample ID: **MAB7**

***R_1_*=3.14%**

Crystal Data and Experimental


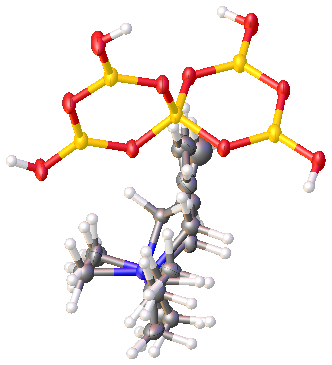


**Experimental.** Single colourless block crystals of **MAB7** recrystallised from a mixture of water and methanol. A suitable crystal with dimensions 0.140 × 0.120 × 0.040 mm^3^ was selected and mounted on a Rigaku 007HF equipped with Varimax confocal mirrors and an AFC11 goniometer and HyPix 6000 detector diffractometer. The crystal was kept at a steady *T* = 100(2) K during data collection. The structure was solved with the **ShelXT** 2018/2 (Sheldrick, 2018) solution program using dual methods and by using **Olex2** (Dolomanov et al., 2009) as the graphical interface. The model was refined with **ShelXL** 2018/3 (Sheldrick, 2015) using full matrix least squares minimisation on ***F*^2^**.

**Crystal Data.** C_6_H_18_B_5_NO_10_, *M_r_* = 318.26, monoclinic, *P*2_1_/*c* (No. 14), a = 9.54050(10) Å, b = 16.1031(2) Å, c = 9.43280(10) Å, *β* = 90.1710(10)^°^, *α* = *γ* = 90^°^, *V* = 1449.17(3) Å^3^, *T* = 100(2) K, *Z* = 4, *Z'* = 1, *μ*(Cu K*_α_*) = 1.096, 13531 reflections measured, 2661 unique (*R_int_* = 0.0236) which were used in all calculations. The final *wR_2_* was 0.0845 (all data) and *R_1_* was 0.0314 (I > 2(I)).

| **Compound** | **MAB7** |
| --- | --- |
|  |  |
| Formula | C_6_H_18_B_5_NO_10_ |
| *D_calc._*/ g cm^-3^ | 1.459 |
| *μ*/mm^-1^ | 1.096 |
| Formula Weight | 318.26 |
| Colour | colourless |
| Shape | block |
| Size/mm^3^ | 0.140×0.120×0.040 |
| *T*/K | 100(2) |
| Crystal System | monoclinic |
| Space Group | *P*2_1_/*c* |
| *a*/Å | 9.54050(10) |
| *b*/Å | 16.1031(2) |
| *c*/Å | 9.43280(10) |
| *α*/^°^ | 90 |
| *β*/^°^ | 90.1710(10) |
| *γ*/^°^ | 90 |
| V/Å^3^ | 1449.17(3) |
| *Z* | 4 |
| *Z'* | 1 |
| Wavelength/Å | 1.54178 |
| Radiation type | Cu K*_α_* |
| *Θ_min_*/^°^ | 4.635 |
| *Θ_max_*/^°^ | 68.183 |
| Measured Refl's. | 13531 |
| Ind't Refl's | 2661 |
| Refl's with I > 2(I) | 2427 |
| *R_int_* | 0.0236 |
| Parameters | 271 |
| Restraints | 562 |
| Largest Peak | 0.211 |
| Deepest Hole | -0.238 |
| GooF | 1.068 |
| *wR_2_* (all data) | 0.0845 |
| *wR_2_* | 0.0827 |
| *R_1_* (all data) | 0.0342 |
| *R_1_* | 0.0314 |

**Table 1**: Fractional Atomic Coordinates (×10^4^) and Equivalent Isotropic Displacement Parameters (Å^2^×10^3^) for **MAB7**. *U_eq_* is defined as 1/3 of the trace of the orthogonalised *U_ij_*.

| **Atom** | **x** | **y** | **z** | ***U_eq_*** |
| --- | --- | --- | --- | --- |
| O1 | 2409.8(8) | 6740.8(5) | 5128.6(8) | 20.45(19) |
| O2 | -25.8(8) | 6453.1(5) | 4881.2(9) | 24.0(2) |
| O3 | 1612.6(8) | 5749.2(5) | 3386.0(8) | 19.38(19) |
| O4 | 4013.8(8) | 5756.8(5) | 4148.3(8) | 18.75(19) |
| O5 | 5567.0(8) | 6652.6(5) | 2890.6(9) | 22.4(2) |
| O6 | 3099.4(8) | 6869.5(5) | 2694.6(8) | 21.0(2) |
| O7 | 734.2(9) | 7382.8(6) | 6628.2(10) | 32.0(2) |
| O8 | -770.4(8) | 5421.2(6) | 3242.0(9) | 26.8(2) |
| O9 | 6488.5(8) | 5555.0(5) | 4199.6(9) | 23.6(2) |
| O10 | 4722.5(8) | 7674.0(6) | 1362.9(10) | 29.7(2) |
| B1 | 2767.9(12) | 6272.1(8) | 3833.6(13) | 17.6(3) |
| B2 | 1065.6(13) | 6861.7(9) | 5547.5(14) | 21.7(3) |
| B3 | 280.9(13) | 5874.2(8) | 3837.6(14) | 19.5(3) |
| B4 | 5335.3(13) | 5985.4(8) | 3773.5(14) | 19.1(3) |
| B5 | 4429.6(13) | 7072.8(9) | 2319.0(14) | 20.9(3) |
| N1 | 2139(7) | 3811(3) | 917(5) | 22.0(15) |
| C1 | 3063(3) | 4326.1(18) | -38(3) | 30.3(7) |
| C2 | 3033(3) | 5225.5(19) | 243(3) | 32.3(7) |
| C3 | 2984(14) | 5798(5) | -832(12) | 45(3) |
| C4 | 642(6) | 4096(9) | 828(18) | 31(2) |
| C5 | 2629(5) | 3851(3) | 2432(4) | 29.1(12) |
| C6 | 2180(5) | 2930(2) | 385(5) | 34.2(8) |
| N1B | 2137(11) | 3837(5) | 1063(8) | 25(3) |
| C1B | 3144(5) | 4553(3) | 872(5) | 25.2(11) |
| C2B | 3134(8) | 4910(4) | -536(10) | 26(2) |
| C3B | 2866(18) | 5735(6) | -738(13) | 23(3) |
| C4B | 673(11) | 4089(15) | 670(30) | 32(5) |
| C5B | 2204(9) | 3531(5) | 2554(7) | 36(3) |
| C6B | 2613(8) | 3118(5) | 173(10) | 22.3(19) |
| N1C | 1911(8) | 3849(5) | 981(10) | 20(3) |
| C1C | 2373(5) | 4704(3) | 564(5) | 17.8(12) |
| C2C | 2975(10) | 4903(5) | -736(9) | 23(3) |
| C3C | 3317(16) | 5711(8) | -1097(17) | 58(5) |
| C4C | 343(8) | 3828(5) | 1004(10) | 30(2) |
| C5C | 2342(16) | 3702(10) | 2506(11) | 14(3) |
| C6C | 2406(15) | 3188(6) | -18(11) | 33(3) |

**Table 2**: Anisotropic Displacement Parameters (×10^4^) for **MAB7**. The anisotropic displacement factor exponent takes the form: *-2π^2^[h^2^a*^2^ × U_11_+ ... +2hka* × b* × U_12_]*

| **Atom** | ***U_11_*** | ***U_22_*** | ***U_33_*** | ***U_23_*** | ***U_13_*** | ***U_12_*** |
| --- | --- | --- | --- | --- | --- | --- |
| O1 | 13.0(4) | 25.1(4) | 23.2(4) | -4.7(3) | 0.0(3) | 0.0(3) |
| O2 | 12.8(4) | 31.0(5) | 28.2(4) | -9.7(4) | 1.5(3) | -0.2(3) |
| O3 | 12.4(4) | 22.6(4) | 23.2(4) | -3.3(3) | 1.3(3) | -1.0(3) |
| O4 | 11.8(4) | 21.4(4) | 23.0(4) | 1.9(3) | 1.0(3) | 1.0(3) |
| O5 | 11.7(4) | 27.9(5) | 27.5(4) | 7.8(4) | 0.1(3) | -0.1(3) |
| O6 | 13.1(4) | 24.7(4) | 25.1(4) | 5.2(3) | -0.1(3) | 0.7(3) |
| O7 | 15.3(4) | 42.2(6) | 38.6(5) | -21.0(4) | 1.3(4) | -1.9(4) |
| O8 | 13.0(4) | 34.0(5) | 33.4(5) | -12.5(4) | 2.9(3) | -2.6(3) |
| O9 | 13.0(4) | 28.3(5) | 29.5(5) | 9.2(4) | 1.6(3) | 1.8(3) |
| O10 | 14.0(4) | 35.9(5) | 39.1(5) | 17.1(4) | -0.7(4) | -0.1(3) |
| B1 | 12.1(6) | 20.4(6) | 20.4(6) | -0.3(5) | -0.2(5) | 1.0(5) |
| B2 | 14.9(6) | 24.9(7) | 25.2(7) | -3.4(5) | -0.3(5) | 0.7(5) |
| B3 | 14.5(6) | 22.3(6) | 21.7(6) | -0.5(5) | 0.9(5) | 0.1(5) |
| B4 | 14.3(6) | 23.4(7) | 19.7(6) | -0.6(5) | 0.7(5) | -0.2(5) |
| B5 | 14.7(6) | 24.6(7) | 23.5(6) | 0.9(5) | -1.0(5) | -0.5(5) |
| N1 | 14.0(19) | 27(2) | 25(2) | -1.1(10) | 3.1(13) | 2.7(11) |
| C1 | 25.4(14) | 39.4(17) | 26.1(14) | -5.6(12) | 5.2(11) | -4.8(12) |
| C2 | 30.2(15) | 37.3(16) | 29.6(15) | -6.0(14) | 3.5(12) | -6.9(12) |
| C3 | 41(4) | 35(3) | 59(4) | 9(2) | -4(3) | -12(3) |
| C4 | 15(2) | 40(4) | 38(5) | -1(3) | -0.9(15) | 6.9(16) |
| C5 | 32(3) | 26(2) | 29.2(18) | 2.6(13) | -4.6(15) | -3.9(19) |
| C6 | 31(2) | 24.4(17) | 47(2) | -11.8(17) | -6.3(16) | 4.9(16) |

**Table 3**: Bond Lengths in Å for **MAB7**.

| **Atom** | **Atom** | **Length/Å** |
| --- | --- | --- |
| O1 | B1 | 1.4769(14) |
| O1 | B2 | 1.3571(15) |
| O2 | B2 | 1.3813(15) |
| O2 | B3 | 1.3877(16) |
| O3 | B1 | 1.4489(14) |
| O3 | B3 | 1.3563(15) |
| O4 | B1 | 1.4790(14) |
| O4 | B4 | 1.3611(15) |
| O5 | B4 | 1.3777(16) |
| O5 | B5 | 1.3865(15) |
| O6 | B1 | 1.4771(15) |
| O6 | B5 | 1.3587(15) |
| O7 | B2 | 1.3584(16) |
| O8 | B3 | 1.3602(15) |
| O9 | B4 | 1.3601(15) |
| O10 | B5 | 1.3529(16) |
| N1 | C1 | 1.510(5) |
| N1 | C4 | 1.502(5) |
| N1 | C5 | 1.503(5) |
| N1 | C6 | 1.506(5) |
| C1 | C2 | 1.473(4) |
| C2 | C3 | 1.371(6) |
| N1B | C1B | 1.511(7) |
| N1B | C4B | 1.500(7) |
| N1B | C5B | 1.492(7) |
| N1B | C6B | 1.501(7) |
| C1B | C2B | 1.448(9) |
| C2B | C3B | 1.367(8) |
| N1C | C1C | 1.498(7) |
| N1C | C4C | 1.497(7) |
| N1C | C5C | 1.514(7) |
| N1C | C6C | 1.499(7) |
| C1C | C2C | 1.392(7) |
| C2C | C3C | 1.384(8) |

**Table 4**: Bond Angles in ^°^ for **MAB7**.

| **Atom** | **Atom** | **Atom** | **Angle/^°^** |
| --- | --- | --- | --- |
| B2 | O1 | B1 | 122.36(9) |
| B2 | O2 | B3 | 118.85(9) |
| B3 | O3 | B1 | 122.33(9) |
| B4 | O4 | B1 | 122.73(9) |
| B4 | O5 | B5 | 119.26(9) |
| B5 | O6 | B1 | 123.28(9) |
| O1 | B1 | O4 | 107.97(9) |
| O1 | B1 | O6 | 108.62(10) |
| O3 | B1 | O1 | 111.12(9) |
| O3 | B1 | O4 | 110.04(10) |
| O3 | B1 | O6 | 109.32(9) |
| O6 | B1 | O4 | 109.74(9) |
| O1 | B2 | O2 | 120.72(11) |
| O1 | B2 | O7 | 121.96(11) |
| O7 | B2 | O2 | 117.32(10) |
| O3 | B3 | O2 | 121.48(11) |
| O3 | B3 | O8 | 118.75(11) |
| O8 | B3 | O2 | 119.77(10) |
| O4 | B4 | O5 | 121.23(10) |
| O9 | B4 | O4 | 122.32(11) |
| O9 | B4 | O5 | 116.41(10) |
| O6 | B5 | O5 | 120.77(11) |
| O10 | B5 | O5 | 116.46(10) |
| O10 | B5 | O6 | 122.76(11) |
| C4 | N1 | C1 | 110.8(6) |
| C4 | N1 | C5 | 109.4(8) |
| C4 | N1 | C6 | 107.2(7) |
| C5 | N1 | C1 | 111.3(4) |
| C5 | N1 | C6 | 110.4(4) |
| C6 | N1 | C1 | 107.6(4) |
| C2 | C1 | N1 | 114.9(3) |
| C3 | C2 | C1 | 122.0(6) |
| C4B | N1B | C1B | 110.9(8) |
| C4B | N1B | C6B | 110.7(13) |
| C5B | N1B | C1B | 109.8(6) |
| C5B | N1B | C4B | 111.0(12) |
| C5B | N1B | C6B | 105.1(7) |
| C6B | N1B | C1B | 109.2(7) |
| C2B | C1B | N1B | 114.2(5) |
| C3B | C2B | C1B | 121.0(9) |
| C1C | N1C | C5C | 108.3(7) |
| C1C | N1C | C6C | 113.2(7) |
| C4C | N1C | C1C | 108.7(6) |
| C4C | N1C | C5C | 104.5(8) |
| C4C | N1C | C6C | 108.0(9) |
| C6C | N1C | C5C | 113.6(9) |
| C2C | C1C | N1C | 124.4(6) |
| C3C | C2C | C1C | 122.1(9) |

**Table 5**: Torsion Angles in ^°^ for **MAB7**.

| **Atom** | **Atom** | **Atom** | **Atom** | **Angle/^°^** |
| --- | --- | --- | --- | --- |
| B1 | O1 | B2 | O2 | 8.94(18) |
| B1 | O1 | B2 | O7 | -171.11(11) |
| B1 | O3 | B3 | O2 | -7.42(17) |
| B1 | O3 | B3 | O8 | 172.26(10) |
| B1 | O4 | B4 | O5 | -9.86(17) |
| B1 | O4 | B4 | O9 | 172.67(10) |
| B1 | O6 | B5 | O5 | 7.36(17) |
| B1 | O6 | B5 | O10 | -173.96(11) |
| B2 | O1 | B1 | O3 | -19.35(15) |
| B2 | O1 | B1 | O4 | -140.11(10) |
| B2 | O1 | B1 | O6 | 100.94(12) |
| B2 | O2 | B3 | O3 | -5.13(18) |
| B2 | O2 | B3 | O8 | 175.19(11) |
| B3 | O2 | B2 | O1 | 4.25(18) |
| B3 | O2 | B2 | O7 | -175.70(11) |
| B3 | O3 | B1 | O1 | 18.48(15) |
| B3 | O3 | B1 | O4 | 138.01(10) |
| B3 | O3 | B1 | O6 | -101.40(12) |
| B4 | O4 | B1 | O1 | -99.10(11) |
| B4 | O4 | B1 | O3 | 139.47(10) |
| B4 | O4 | B1 | O6 | 19.13(14) |
| B4 | O5 | B5 | O6 | 4.45(17) |
| B4 | O5 | B5 | O10 | -174.31(11) |
| B5 | O5 | B4 | O4 | -3.19(17) |
| B5 | O5 | B4 | O9 | 174.42(10) |
| B5 | O6 | B1 | O1 | 99.88(12) |
| B5 | O6 | B1 | O3 | -138.71(10) |
| B5 | O6 | B1 | O4 | -17.95(15) |
| N1 | C1 | C2 | C3 | -137.7(7) |
| C4 | N1 | C1 | C2 | 57.2(8) |
| C5 | N1 | C1 | C2 | -64.8(5) |
| C6 | N1 | C1 | C2 | 174.1(4) |
| N1B | C1B | C2B | C3B | 123.2(11) |
| C4B | N1B | C1B | C2B | -55.6(13) |
| C5B | N1B | C1B | C2B | -178.6(7) |
| C6B | N1B | C1B | C2B | 66.7(9) |
| N1C | C1C | C2C | C3C | 176.5(10) |
| C4C | N1C | C1C | C2C | -108.8(9) |
| C5C | N1C | C1C | C2C | 138.1(9) |
| C6C | N1C | C1C | C2C | 11.2(11) |

**Table 6**: Hydrogen Fractional Atomic Coordinates (×10^4^) and Equivalent Isotropic Displacement Parameters (Å^2^×10^3^) for **MAB7**. *U_eq_* is defined as 1/3 of the trace of the orthogonalised *U_ij_*.

| **Atom** | **x** | **y** | **z** | ***U_eq_*** |
| --- | --- | --- | --- | --- |
| H7 | 1467.07 | 7609.2 | 6937.33 | 48 |
| H8 | -1538.68 | 5558.23 | 3606.37 | 40 |
| H9 | 6241.97 | 5155.66 | 4714.6 | 35 |
| H10 | 3970.99 | 7866.49 | 1032.95 | 45 |
| H1A | 4040.63 | 4128.36 | 62.17 | 36 |
| H1B | 2772.53 | 4230.33 | -1033.22 | 36 |
| H2 | 3047.54 | 5414.54 | 1196.48 | 39 |
| H3A | 2968.25 | 5618.74 | -1791.75 | 54 |
| H3B | 2965.17 | 6374.55 | -617.84 | 54 |
| H4A | 563.34 | 4656.67 | 1225.26 | 46 |
| H4B | 45.04 | 3715.23 | 1367.11 | 46 |
| H4C | 339.61 | 4102.69 | -165.65 | 46 |
| H5A | 2545.57 | 4422.39 | 2779.97 | 44 |
| H5B | 3610.03 | 3674.04 | 2488.04 | 44 |
| H5C | 2049.97 | 3483.1 | 3014.75 | 44 |
| H6A | 1607.87 | 2577.43 | 1002.09 | 51 |
| H6B | 3149.95 | 2729.97 | 392 | 51 |
| H6C | 1808.51 | 2909.18 | -583.63 | 51 |
| H1BA | 2907.11 | 4992.62 | 1564.95 | 30 |
| H1BB | 4104.22 | 4357.3 | 1091.21 | 30 |
| H2B | 3316.83 | 4565.1 | -1332.18 | 32 |
| H3BA | 2682.69 | 6083.45 | 52.73 | 28 |
| H3BB | 2863.45 | 5960.34 | -1668.73 | 28 |
| H4BA | 358.1 | 4531.91 | 1308.57 | 48 |
| H4BB | 47.1 | 3609.09 | 762.17 | 48 |
| H4BC | 656.05 | 4289.61 | -308.06 | 48 |
| H5BA | 3161.87 | 3350.49 | 2772.15 | 54 |
| H5BB | 1559.69 | 3061.98 | 2668.14 | 54 |
| H5BC | 1935.1 | 3979.1 | 3201.16 | 54 |
| H6BA | 2182.92 | 2605.6 | 524.29 | 33 |
| H6BB | 3635.48 | 3070.48 | 228.42 | 33 |
| H6BC | 2330.32 | 3209.4 | -814.53 | 33 |
| H1CA | 1540.15 | 5066.14 | 660.84 | 21 |
| H1CB | 3048.92 | 4886.37 | 1299.09 | 21 |
| H2C | 3157.1 | 4470.83 | -1395.18 | 28 |
| H3CA | 3141.26 | 6150.9 | -449.79 | 69 |
| H3CB | 3726.18 | 5826.43 | -1992.73 | 69 |
| H4CA | -6.56 | 4271.47 | 1620.35 | 45 |
| H4CB | 27.67 | 3289.04 | 1366.88 | 45 |
| H4CC | -20.02 | 3908.53 | 40.99 | 45 |
| H5CA | 1979.76 | 4151.42 | 3101.6 | 22 |
| H5CB | 3367.43 | 3688.28 | 2573.45 | 22 |
| H5CC | 1959.27 | 3170.43 | 2830.68 | 22 |
| H6CA | 2332.33 | 3390.43 | -995.13 | 49 |
| H6CB | 1824.21 | 2691.07 | 93.66 | 49 |
| H6CC | 3384.93 | 3050.85 | 195 | 49 |

**Table 7**: Hydrogen Bond information for **MAB7**.

| **D** | **H** | **A** | **d(D-H)/Å** | **d(H-A)/Å** | **d(D-A)/Å** | **D-H-A/deg** |
| --- | --- | --- | --- | --- | --- | --- |
| O7 | H7 | O6^1^ | 0.84 | 1.91 | 2.7458(11) | 178.2 |
| O8 | H8 | O9^2^ | 0.84 | 1.97 | 2.7777(11) | 162.5 |
| O9 | H9 | O4^3^ | 0.84 | 1.84 | 2.6693(11) | 171.3 |
| O10 | H10 | O1^4^ | 0.84 | 1.83 | 2.6639(11) | 173.9 |

––––

^1^+x,3/2-y,1/2+z; ^2^-1+x,+y,+z; ^3^1-x,1-y,1-z; ^4^+x,3/2-y,-1/2+z

**Table 8**: Atomic Occupancies for all atoms that are not fully occupied in **MAB7**.

| **Atom** | **Occupancy** |
| --- | --- |
| N1 | 0.485(3) |
| C1 | 0.485(3) |
| H1A | 0.485(3) |
| H1B | 0.485(3) |
| C2 | 0.485(3) |
| H2 | 0.485(3) |
| C3 | 0.485(3) |
| H3A | 0.485(3) |
| H3B | 0.485(3) |
| C4 | 0.485(3) |
| H4A | 0.485(3) |
| H4B | 0.485(3) |
| H4C | 0.485(3) |
| C5 | 0.485(3) |
| H5A | 0.485(3) |
| H5B | 0.485(3) |
| H5C | 0.485(3) |
| C6 | 0.485(3) |
| H6A | 0.485(3) |
| H6B | 0.485(3) |
| H6C | 0.485(3) |
| N1B | 0.292(3) |
| C1B | 0.292(3) |
| H1BA | 0.292(3) |
| H1BB | 0.292(3) |
| C2B | 0.292(3) |
| H2B | 0.292(3) |
| C3B | 0.292(3) |
| H3BA | 0.292(3) |
| H3BB | 0.292(3) |
| C4B | 0.292(3) |
| H4BA | 0.292(3) |
| H4BB | 0.292(3) |
| H4BC | 0.292(3) |
| C5B | 0.292(3) |
| H5BA | 0.292(3) |
| H5BB | 0.292(3) |
| H5BC | 0.292(3) |
| C6B | 0.292(3) |
| H6BA | 0.292(3) |
| H6BB | 0.292(3) |
| H6BC | 0.292(3) |
| N1C | 0.223(3) |
| C1C | 0.223(3) |
| H1CA | 0.223(3) |
| H1CB | 0.223(3) |
| C2C | 0.223(3) |
| H2C | 0.223(3) |
| C3C | 0.223(3) |
| H3CA | 0.223(3) |
| H3CB | 0.223(3) |
| C4C | 0.223(3) |
| H4CA | 0.223(3) |
| H4CB | 0.223(3) |
| H4CC | 0.223(3) |
| C5C | 0.223(3) |
| H5CA | 0.223(3) |
| H5CB | 0.223(3) |
| H5CC | 0.223(3) |
| C6C | 0.223(3) |
| H6CA | 0.223(3) |
| H6CB | 0.223(3) |
| H6CC | 0.223(3) |
