# Supplementary material for: Pentaborate(1-) Salts and a Tetraborate(2-) Salt Derived from C2- or C3-Linked Bis(alkylammonium) Dications: Synthesis, Characterization, and Structural (XRD) Studies
Source: Molecules. 2019 Dec 23;25(1):53. doi: 10.3390/molecules25010053 (PMC6982793; doi:10.3390/molecules25010053)
Supplement: Supplementary file 1 [file molecules-25-00053-s001.zip › MAB8.docx]

**MAB8**


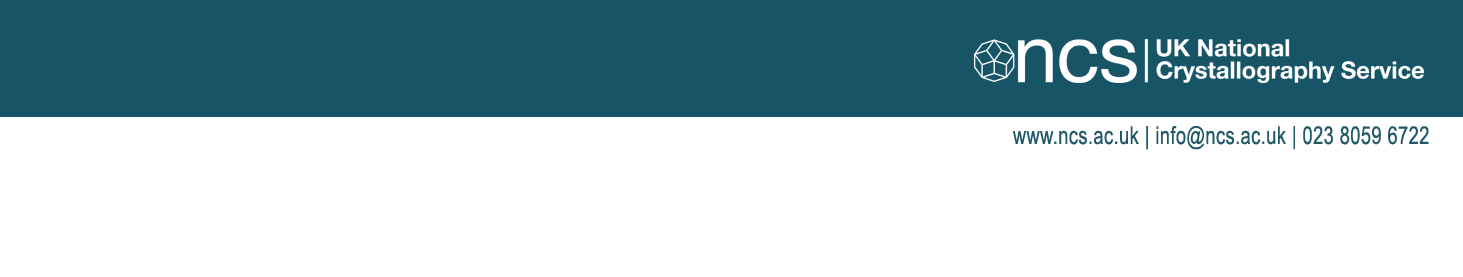


Submitted by: **None**

None

Solved by: **None**

Sample ID: **MAB8**

***R_1_*=3.51%**

Crystal Data and Experimental


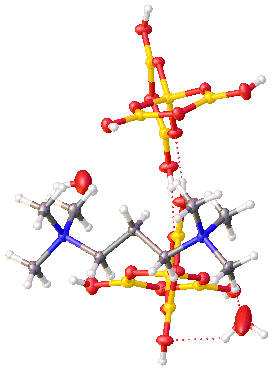


**Experimental.** Single colourless blade crystals of **MAB8** recrystallised from water. A suitable crystal with dimensions 0.260 × 0.140 × 0.035 mm^3^ was selected and mounted on a Rigaku FRE+ equipped with HF Varimax confocal mirrors and an AFC12 goniometer and HG Saturn 724+ detector diffractometer. The crystal was kept at a steady *T* = 100(2) K during data collection. The structure was solved with the **ShelXT** 2018/2 (Sheldrick, 2018) solution program using dual methods and by using **Olex2** (Dolomanov et al., 2009) as the graphical interface. The model was refined with **ShelXL** 2018/3 (Sheldrick, 2015) using full matrix least squares minimisation on ***F*^2^**.

**Crystal Data.** C_9_H_33_B_10_N_2_O_20.5_, *M_r_* = 605.47, monoclinic, *C*2/*c* (No. 15), a = 26.8754(5) Å, b = 11.5269(2) Å, c = 17.9383(4) Å, *β* = 103.154(2)^°^, *α* = *γ* = 90^°^, *V* = 5411.30(19) Å^3^, *T* = 100(2) K, *Z* = 8, *Z'* = 1, *μ*(Mo K*_α_*) = 0.132, 31515 reflections measured, 6191 unique (*R_int_* = 0.0294) which were used in all calculations. The final *wR_2_* was 0.0919 (all data) and *R_1_* was 0.0351 (I > 2(I)).

| **Compound** | **MAB8** |
| --- | --- |
|  |  |
| Formula | C_9_H_33_B_10_N_2_O_20.5_ |
| *D_calc._*/ g cm^-3^ | 1.486 |
| *μ*/mm^-1^ | 0.132 |
| Formula Weight | 605.47 |
| Colour | colourless |
| Shape | blade |
| Size/mm^3^ | 0.260×0.140×0.035 |
| *T*/K | 100(2) |
| Crystal System | monoclinic |
| Space Group | *C*2/*c* |
| *a*/Å | 26.8754(5) |
| *b*/Å | 11.5269(2) |
| *c*/Å | 17.9383(4) |
| *α*/^°^ | 90 |
| *β*/^°^ | 103.154(2) |
| *γ*/^°^ | 90 |
| V/Å^3^ | 5411.30(19) |
| *Z* | 8 |
| *Z'* | 1 |
| Wavelength/Å | 0.71075 |
| Radiation type | Mo K*_α_* |
| *Θ_min_*/^°^ | 2.332 |
| *Θ_max_*/^°^ | 27.482 |
| Measured Refl's. | 31515 |
| Ind't Refl's | 6191 |
| Refl's with I > 2(I) | 5188 |
| *R_int_* | 0.0294 |
| Parameters | 409 |
| Restraints | 1 |
| Largest Peak | 0.295 |
| Deepest Hole | -0.219 |
| GooF | 1.029 |
| *wR_2_* (all data) | 0.0919 |
| *wR_2_* | 0.0863 |
| *R_1_* (all data) | 0.0447 |
| *R_1_* | 0.0351 |

**Table 1**: Fractional Atomic Coordinates (×10^4^) and Equivalent Isotropic Displacement Parameters (Å^2^×10^3^) for **MAB8**. *U_eq_* is defined as 1/3 of the trace of the orthogonalised *U_ij_*.

| **Atom** | **x** | **y** | **z** | ***U_eq_*** |
| --- | --- | --- | --- | --- |
| N1 | 5270.0(3) | 2544.2(8) | 5594.3(5) | 17.2(2) |
| N2 | 7180.2(4) | 2530.6(8) | 6850.8(5) | 18.0(2) |
| C1 | 5769.2(4) | 1929.0(10) | 5921.5(7) | 18.1(2) |
| C2 | 6229.8(4) | 2722.7(10) | 6189.4(6) | 18.1(2) |
| C3 | 6672.2(4) | 1929.3(10) | 6555.7(7) | 18.8(2) |
| C4 | 5115.6(4) | 3297.4(11) | 6185.7(7) | 23.4(3) |
| C5 | 5299.8(5) | 3269.5(11) | 4911.9(7) | 22.4(3) |
| C6 | 4870.7(4) | 1623.0(10) | 5346.5(7) | 21.3(2) |
| C7 | 7363.1(5) | 3102.2(12) | 6213.3(7) | 25.5(3) |
| C8 | 7557.3(4) | 1610.2(11) | 7205.1(7) | 23.5(3) |
| C9 | 7151.2(5) | 3412.1(11) | 7449.4(7) | 25.6(3) |
| O11 | 6188.6(3) | 7543.8(6) | 3946.8(4) | 16.12(17) |
| O12 | 5932.9(3) | 6486.3(7) | 2781.3(4) | 20.30(18) |
| O13 | 6390.3(3) | 5504.1(7) | 3892.4(4) | 18.81(18) |
| O14 | 6052.8(3) | 6241.6(7) | 4924.8(4) | 17.49(17) |
| O15 | 6753.0(3) | 6058.3(7) | 6013.0(4) | 19.04(18) |
| O16 | 6905.9(3) | 6770.1(7) | 4831.6(4) | 17.44(17) |
| O17 | 5827.6(3) | 8489.8(7) | 2766.0(5) | 22.74(19) |
| O18 | 6129.0(4) | 4527.3(7) | 2691.4(4) | 25.4(2) |
| O19 | 5891.3(3) | 5721.3(7) | 6102.5(4) | 20.73(18) |
| O20 | 7570.5(3) | 6703.0(8) | 5965.0(5) | 23.8(2) |
| B11 | 6384.3(5) | 6507.7(11) | 4409.2(7) | 15.8(2) |
| B12 | 5984.8(5) | 7523.0(11) | 3180.5(7) | 16.1(3) |
| B13 | 6151.6(5) | 5491.3(11) | 3141.7(7) | 17.4(3) |
| B14 | 6235.3(5) | 6010.0(11) | 5678.9(7) | 16.4(3) |
| B15 | 7078.2(5) | 6522.0(11) | 5589.9(7) | 17.6(3) |
| O1 | 5644.7(3) | 1489.9(7) | 2572.0(4) | 18.17(17) |
| O2 | 5844.8(3) | 1018.8(7) | 1376.2(4) | 21.87(19) |
| O3 | 6525.2(3) | 1313.0(7) | 2476.6(4) | 18.06(17) |
| O4 | 6234.2(3) | 418.3(7) | 3511.2(4) | 17.24(17) |
| O5 | 6516.4(3) | 1518.6(7) | 4652.0(4) | 22.88(19) |
| O6 | 6311.7(3) | 2506.5(7) | 3448.9(4) | 16.65(17) |
| O7 | 4991.2(3) | 1109.5(8) | 1458.3(5) | 26.3(2) |
| O8 | 6715.0(3) | 973.9(8) | 1286.5(5) | 23.45(19) |
| O9 | 6392.2(3) | -477.3(7) | 4738.1(4) | 21.62(19) |
| O10 | 6609.9(4) | 3516.9(7) | 4625.0(5) | 28.7(2) |
| B1 | 6181.7(5) | 1433.2(11) | 2994.1(7) | 16.2(3) |
| B2 | 5491.6(5) | 1215.9(11) | 1815.8(7) | 19.2(3) |
| B3 | 6360.4(5) | 1109.8(11) | 1717.3(7) | 17.7(3) |
| B4 | 6380.7(5) | 474.1(11) | 4284.4(7) | 16.7(3) |
| B5 | 6476.0(5) | 2530.4(11) | 4225.1(7) | 18.7(3) |
| O21 | 5126.7(19) | 4084(5) | 3064(3) | 68.6(18) |
| O22 | 7512(2) | 98(7) | 5744(6) | 124(4) |

**Table 2**: Anisotropic Displacement Parameters (×10^4^) for **MAB8**. The anisotropic displacement factor exponent takes the form: *-2π^2^[h^2^a*^2^ × U_11_+ ... +2hka* × b* × U_12_]*

| **Atom** | ***U_11_*** | ***U_22_*** | ***U_33_*** | ***U_23_*** | ***U_13_*** | ***U_12_*** |
| --- | --- | --- | --- | --- | --- | --- |
| N1 | 13.7(5) | 20.2(5) | 15.9(5) | -0.9(4) | -0.7(4) | -0.1(4) |
| N2 | 14.2(5) | 21.6(5) | 16.3(5) | 1.3(4) | -0.4(4) | 2.0(4) |
| C1 | 15.3(5) | 19.0(6) | 18.3(6) | 0.5(4) | 0.4(4) | 2.2(4) |
| C2 | 14.7(5) | 21.1(6) | 16.2(5) | -0.1(4) | -1.0(4) | 1.2(4) |
| C3 | 15.3(5) | 20.1(6) | 19.1(6) | 0.4(4) | 0.4(4) | 0.4(4) |
| C4 | 16.9(6) | 29.3(7) | 22.1(6) | -7.3(5) | 0.8(5) | 1.9(5) |
| C5 | 19.6(6) | 25.7(6) | 19.5(6) | 4.0(5) | -0.6(5) | 0.2(5) |
| C6 | 16.8(6) | 23.0(6) | 21.8(6) | -2.4(5) | -0.8(5) | -4.6(4) |
| C7 | 18.7(6) | 33.7(7) | 22.6(6) | 7.3(5) | 1.8(5) | -2.3(5) |
| C8 | 16.6(6) | 25.1(6) | 26.5(6) | 4.8(5) | -0.1(5) | 6.5(5) |
| C9 | 19.2(6) | 29.5(7) | 24.9(6) | -9.1(5) | -1.9(5) | 2.1(5) |
| O11 | 18.9(4) | 14.7(4) | 12.9(4) | 0.2(3) | -0.2(3) | 0.2(3) |
| O12 | 27.8(4) | 16.8(4) | 13.0(4) | -0.1(3) | -2.2(3) | 1.3(3) |
| O13 | 23.9(4) | 15.5(4) | 14.6(4) | 0.3(3) | -0.7(3) | 2.0(3) |
| O14 | 15.7(4) | 20.7(4) | 14.2(4) | 1.9(3) | -0.6(3) | -1.7(3) |
| O15 | 16.8(4) | 22.8(4) | 15.0(4) | 3.4(3) | -1.7(3) | -2.8(3) |
| O16 | 15.6(4) | 21.7(4) | 13.6(4) | 1.3(3) | 0.3(3) | -1.4(3) |
| O17 | 32.9(5) | 16.4(4) | 14.9(4) | 0.0(3) | -3.1(3) | 0.8(3) |
| O18 | 42.7(5) | 16.9(4) | 14.1(4) | 1.0(3) | 1.4(4) | 4.7(4) |
| O19 | 18.6(4) | 27.4(4) | 14.5(4) | 3.2(3) | 0.3(3) | -2.3(3) |
| O20 | 16.2(4) | 36.4(5) | 16.8(4) | 5.3(4) | -0.3(3) | -4.5(3) |
| B11 | 16.7(6) | 15.5(6) | 13.3(6) | 0.0(5) | -0.7(5) | -0.3(5) |
| B12 | 14.4(6) | 18.2(6) | 14.9(6) | 0.1(5) | 1.7(5) | -0.7(5) |
| B13 | 19.6(6) | 18.1(6) | 14.2(6) | 0.7(5) | 3.1(5) | -1.1(5) |
| B14 | 18.9(6) | 14.0(6) | 14.5(6) | 0.7(5) | 0.0(5) | -0.4(5) |
| B15 | 17.8(6) | 18.0(6) | 15.3(6) | 0.5(5) | 0.1(5) | 0.0(5) |
| O1 | 17.6(4) | 21.9(4) | 13.3(4) | -0.5(3) | -0.2(3) | 0.2(3) |
| O2 | 22.7(4) | 28.0(5) | 12.9(4) | -2.5(3) | -0.2(3) | -0.2(3) |
| O3 | 17.8(4) | 21.4(4) | 13.9(4) | -1.5(3) | 1.3(3) | -1.1(3) |
| O4 | 21.5(4) | 15.3(4) | 13.0(4) | 0.2(3) | -0.1(3) | -1.1(3) |
| O5 | 37.7(5) | 16.0(4) | 12.5(4) | -0.2(3) | 0.7(4) | -0.5(3) |
| O6 | 20.5(4) | 15.0(4) | 12.9(4) | -0.4(3) | 0.6(3) | -0.6(3) |
| O7 | 19.9(4) | 42.1(5) | 14.9(4) | -2.5(4) | -0.4(3) | -1.9(4) |
| O8 | 26.4(5) | 28.4(5) | 15.1(4) | -3.8(4) | 3.8(3) | -2.4(4) |
| O9 | 34.1(5) | 16.0(4) | 13.6(4) | -0.6(3) | 3.0(3) | -1.2(3) |
| O10 | 51.7(6) | 15.4(4) | 14.1(4) | 0.7(3) | -2.8(4) | -0.4(4) |
| B1 | 18.1(6) | 16.9(6) | 11.9(6) | -0.1(5) | 0.1(5) | -0.6(5) |
| B2 | 21.8(7) | 19.3(6) | 14.2(6) | 1.3(5) | -0.9(5) | -0.4(5) |
| B3 | 22.7(7) | 13.7(6) | 15.5(6) | 0.0(5) | 1.6(5) | -0.8(5) |
| B4 | 16.9(6) | 17.8(6) | 14.7(6) | -0.3(5) | 2.0(5) | 0.9(5) |
| B5 | 21.7(7) | 18.4(6) | 14.7(6) | 0.3(5) | 1.5(5) | 1.2(5) |
| O21 | 42(3) | 72(4) | 96(5) | -13(3) | 26(3) | -5(2) |
| O22 | 39(3) | 112(6) | 225(10) | -117(7) | 38(5) | -15(4) |

**Table 3**: Bond Lengths in Å for **MAB8**.

| **Atom** | **Atom** | **Length/Å** |
| --- | --- | --- |
| N1 | C1 | 1.5129(14) |
| N1 | C4 | 1.5006(15) |
| N1 | C5 | 1.4994(15) |
| N1 | C6 | 1.5033(14) |
| N2 | C3 | 1.5151(14) |
| N2 | C7 | 1.4963(15) |
| N2 | C8 | 1.5045(14) |
| N2 | C9 | 1.4932(15) |
| C1 | C2 | 1.5262(15) |
| C2 | C3 | 1.5254(15) |
| O11 | B11 | 1.4807(14) |
| O11 | B12 | 1.3591(14) |
| O12 | B12 | 1.3838(15) |
| O12 | B13 | 1.3806(15) |
| O13 | B11 | 1.4848(15) |
| O13 | B13 | 1.3534(15) |
| O14 | B11 | 1.4555(15) |
| O14 | B14 | 1.3565(14) |
| O15 | B14 | 1.3852(15) |
| O15 | B15 | 1.3882(15) |
| O16 | B11 | 1.4652(14) |
| O16 | B15 | 1.3638(15) |
| O17 | B12 | 1.3529(15) |
| O18 | B13 | 1.3669(15) |
| O19 | B14 | 1.3647(15) |
| O20 | B15 | 1.3571(15) |
| O1 | B1 | 1.4708(14) |
| O1 | B2 | 1.3620(15) |
| O2 | B2 | 1.3839(16) |
| O2 | B3 | 1.3855(15) |
| O3 | B1 | 1.4570(15) |
| O3 | B3 | 1.3533(15) |
| O4 | B1 | 1.4797(14) |
| O4 | B4 | 1.3542(15) |
| O5 | B4 | 1.3813(15) |
| O5 | B5 | 1.3859(15) |
| O6 | B1 | 1.4794(14) |
| O6 | B5 | 1.3619(15) |
| O7 | B2 | 1.3574(15) |
| O8 | B3 | 1.3656(16) |
| O9 | B4 | 1.3619(15) |
| O10 | B5 | 1.3490(15) |

**Table 4**: Bond Angles in ^°^ for **MAB8**.

| **Atom** | **Atom** | **Atom** | **Angle/^°^** |
| --- | --- | --- | --- |
| C4 | N1 | C1 | 111.12(9) |
| C4 | N1 | C6 | 108.90(9) |
| C5 | N1 | C1 | 111.76(9) |
| C5 | N1 | C4 | 109.23(9) |
| C5 | N1 | C6 | 108.64(9) |
| C6 | N1 | C1 | 107.10(9) |
| C7 | N2 | C3 | 111.16(9) |
| C7 | N2 | C8 | 109.06(9) |
| C8 | N2 | C3 | 106.96(9) |
| C9 | N2 | C3 | 111.85(9) |
| C9 | N2 | C7 | 109.26(10) |
| C9 | N2 | C8 | 108.45(9) |
| N1 | C1 | C2 | 115.16(9) |
| C3 | C2 | C1 | 105.85(9) |
| N2 | C3 | C2 | 115.36(9) |
| B12 | O11 | B11 | 124.22(9) |
| B13 | O12 | B12 | 119.53(9) |
| B13 | O13 | B11 | 124.04(9) |
| B14 | O14 | B11 | 122.71(9) |
| B14 | O15 | B15 | 118.31(9) |
| B15 | O16 | B11 | 122.70(9) |
| O11 | B11 | O13 | 109.36(9) |
| O14 | B11 | O11 | 109.58(9) |
| O14 | B11 | O13 | 109.13(9) |
| O14 | B11 | O16 | 111.53(9) |
| O16 | B11 | O11 | 107.41(9) |
| O16 | B11 | O13 | 109.80(9) |
| O11 | B12 | O12 | 120.57(10) |
| O17 | B12 | O11 | 123.02(10) |
| O17 | B12 | O12 | 116.41(10) |
| O13 | B13 | O12 | 120.78(10) |
| O13 | B13 | O18 | 122.96(11) |
| O18 | B13 | O12 | 116.20(10) |
| O14 | B14 | O15 | 121.55(10) |
| O14 | B14 | O19 | 117.78(10) |
| O19 | B14 | O15 | 120.66(10) |
| O16 | B15 | O15 | 120.86(10) |
| O20 | B15 | O15 | 116.88(10) |
| O20 | B15 | O16 | 122.26(11) |
| B2 | O1 | B1 | 122.75(9) |
| B2 | O2 | B3 | 118.86(9) |
| B3 | O3 | B1 | 123.23(9) |
| B4 | O4 | B1 | 124.65(9) |
| B4 | O5 | B5 | 119.39(9) |
| B5 | O6 | B1 | 124.17(9) |
| O1 | B1 | O4 | 107.48(9) |
| O1 | B1 | O6 | 109.13(9) |
| O3 | B1 | O1 | 111.47(9) |
| O3 | B1 | O4 | 109.90(9) |
| O3 | B1 | O6 | 109.06(9) |
| O6 | B1 | O4 | 109.79(9) |
| O1 | B2 | O2 | 120.97(11) |
| O7 | B2 | O1 | 122.26(11) |
| O7 | B2 | O2 | 116.76(10) |
| O3 | B3 | O2 | 121.52(11) |
| O3 | B3 | O8 | 118.61(11) |
| O8 | B3 | O2 | 119.86(10) |
| O4 | B4 | O5 | 120.93(10) |
| O4 | B4 | O9 | 122.38(10) |
| O9 | B4 | O5 | 116.67(10) |
| O6 | B5 | O5 | 120.95(11) |
| O10 | B5 | O5 | 115.96(10) |
| O10 | B5 | O6 | 123.09(11) |

**Table 5**: Torsion Angles in ^°^ for **MAB8**.

| **Atom** | **Atom** | **Atom** | **Atom** | **Angle/^°^** |
| --- | --- | --- | --- | --- |
| N1 | C1 | C2 | C3 | 175.18(9) |
| C1 | C2 | C3 | N2 | 178.58(9) |
| C4 | N1 | C1 | C2 | -62.67(12) |
| C5 | N1 | C1 | C2 | 59.63(12) |
| C6 | N1 | C1 | C2 | 178.50(9) |
| C7 | N2 | C3 | C2 | -62.55(12) |
| C8 | N2 | C3 | C2 | 178.49(9) |
| C9 | N2 | C3 | C2 | 59.89(13) |
| B11 | O11 | B12 | O12 | 2.12(17) |
| B11 | O11 | B12 | O17 | -177.25(10) |
| B11 | O13 | B13 | O12 | 7.91(17) |
| B11 | O13 | B13 | O18 | -174.77(11) |
| B11 | O14 | B14 | O15 | 2.60(17) |
| B11 | O14 | B14 | O19 | -177.17(10) |
| B11 | O16 | B15 | O15 | -3.57(17) |
| B11 | O16 | B15 | O20 | 175.87(10) |
| B12 | O11 | B11 | O13 | 8.01(14) |
| B12 | O11 | B11 | O14 | -111.56(11) |
| B12 | O11 | B11 | O16 | 127.13(10) |
| B12 | O12 | B13 | O13 | 3.86(17) |
| B12 | O12 | B13 | O18 | -173.63(10) |
| B13 | O12 | B12 | O11 | -8.76(16) |
| B13 | O12 | B12 | O17 | 170.65(10) |
| B13 | O13 | B11 | O11 | -13.05(15) |
| B13 | O13 | B11 | O14 | 106.81(11) |
| B13 | O13 | B11 | O16 | -130.67(11) |
| B14 | O14 | B11 | O11 | -132.74(10) |
| B14 | O14 | B11 | O13 | 107.53(11) |
| B14 | O14 | B11 | O16 | -13.95(15) |
| B14 | O15 | B15 | O16 | -9.21(16) |
| B14 | O15 | B15 | O20 | 171.32(10) |
| B15 | O15 | B14 | O14 | 9.75(16) |
| B15 | O15 | B14 | O19 | -170.48(10) |
| B15 | O16 | B11 | O11 | 134.51(10) |
| B15 | O16 | B11 | O13 | -106.66(11) |
| B15 | O16 | B11 | O14 | 14.43(15) |
| B1 | O1 | B2 | O2 | -8.90(17) |
| B1 | O1 | B2 | O7 | 170.34(11) |
| B1 | O3 | B3 | O2 | 0.99(17) |
| B1 | O3 | B3 | O8 | -177.91(10) |
| B1 | O4 | B4 | O5 | -2.84(17) |
| B1 | O4 | B4 | O9 | 175.74(10) |
| B1 | O6 | B5 | O5 | -2.23(18) |
| B1 | O6 | B5 | O10 | 177.54(11) |
| B2 | O1 | B1 | O3 | 12.87(15) |
| B2 | O1 | B1 | O4 | -107.61(11) |
| B2 | O1 | B1 | O6 | 133.38(10) |
| B2 | O2 | B3 | O3 | 4.26(17) |
| B2 | O2 | B3 | O8 | -176.86(10) |
| B3 | O2 | B2 | O1 | -0.30(17) |
| B3 | O2 | B2 | O7 | -179.59(10) |
| B3 | O3 | B1 | O1 | -8.95(15) |
| B3 | O3 | B1 | O4 | 110.10(11) |
| B3 | O3 | B1 | O6 | -129.50(10) |
| B4 | O4 | B1 | O1 | -118.70(11) |
| B4 | O4 | B1 | O3 | 119.83(11) |
| B4 | O4 | B1 | O6 | -0.12(15) |
| B4 | O5 | B5 | O6 | -0.94(17) |
| B4 | O5 | B5 | O10 | 179.28(11) |
| B5 | O5 | B4 | O4 | 3.41(17) |
| B5 | O5 | B4 | O9 | -175.25(10) |
| B5 | O6 | B1 | O1 | 120.20(11) |
| B5 | O6 | B1 | O3 | -117.82(11) |
| B5 | O6 | B1 | O4 | 2.65(15) |

**Table 6**: Hydrogen Fractional Atomic Coordinates (×10^4^) and Equivalent Isotropic Displacement Parameters (Å^2^×10^3^) for **MAB8**. *U_eq_* is defined as 1/3 of the trace of the orthogonalised *U_ij_*.

| **Atom** | **x** | **y** | **z** | ***U_eq_*** |
| --- | --- | --- | --- | --- |
| H1A | 5842.78 | 1396.56 | 5527.06 | 22 |
| H1B | 5726.37 | 1449.48 | 6360.51 | 22 |
| H2A | 6308.86 | 3146.93 | 5750.57 | 22 |
| H2B | 6161.85 | 3293.81 | 6566.33 | 22 |
| H3A | 6579.16 | 1513.47 | 6987.38 | 23 |
| H3B | 6715.84 | 1340.63 | 6173.75 | 23 |
| H4A | 5105.69 | 2831.08 | 6638.95 | 35 |
| H4B | 4776.43 | 3625.77 | 5974.98 | 35 |
| H4C | 5363.92 | 3927.1 | 6329.56 | 35 |
| H5A | 5546.43 | 3898.59 | 5070.84 | 34 |
| H5B | 4962.66 | 3598.93 | 4688.78 | 34 |
| H5C | 5409.82 | 2784.98 | 4530.18 | 34 |
| H6A | 4959.35 | 1151.5 | 4940.96 | 32 |
| H6B | 4537.38 | 1990.06 | 5152.85 | 32 |
| H6C | 4854.94 | 1126.95 | 5784.25 | 32 |
| H7A | 7385.78 | 2524.34 | 5821.74 | 38 |
| H7B | 7700.9 | 3443.72 | 6414.49 | 38 |
| H7C | 7122.27 | 3712.99 | 5986.24 | 38 |
| H8A | 7446.18 | 1251.74 | 7635.34 | 35 |
| H8B | 7894.83 | 1962.22 | 7389.49 | 35 |
| H8C | 7576.5 | 1017 | 6821.28 | 35 |
| H9A | 6917.35 | 4035.26 | 7222.31 | 38 |
| H9B | 7491.9 | 3736.02 | 7655.52 | 38 |
| H9C | 7025.32 | 3043.55 | 7863.27 | 38 |
| H17 | 5925.66 | 9079.39 | 3034.55 | 34 |
| H18 | 6208.27 | 3939.41 | 2969.8 | 38 |
| H19 | 6041.67 | 5673.38 | 6566.33 | 31 |
| H20 | 7727.37 | 7042.03 | 5672.93 | 36 |
| H7 | 4808.7 | 1255.95 | 1769.58 | 39 |
| H8 | 6565.79 | 845.54 | 829.26 | 35 |
| H9 | 6321.6 | -1070.99 | 4462.84 | 32 |
| H10 | 6542.32 | 4089.35 | 4329.44 | 43 |
| H21A | 4808.83 | 3957.97 | 3072.43 | 103 |
| H21B | 5185.07 | 3830.11 | 2634.12 | 103 |
| H22A | 7271.52 | -411.23 | 5588.91 | 186 |
| H22B | 7788.52 | -313.39 | 5889.01 | 186 |

**Table 7**: Hydrogen Bond information for **MAB8**.

| **D** | **H** | **A** | **d(D-H)/Å** | **d(H-A)/Å** | **d(D-A)/Å** | **D-H-A/deg** |
| --- | --- | --- | --- | --- | --- | --- |
| O17 | H17 | O4^1^ | 0.84 | 1.86 | 2.6941(11) | 168.9 |
| O18 | H18 | O6 | 0.84 | 1.85 | 2.6851(11) | 170.3 |
| O19 | H19 | O18^2^ | 0.84 | 1.99 | 2.7903(11) | 158.5 |
| O20 | H20 | O16^3^ | 0.84 | 2.02 | 2.8347(11) | 164.0 |
| O7 | H7 | O1^4^ | 0.84 | 1.90 | 2.7371(12) | 175.0 |
| O8 | H8 | O9^5^ | 0.84 | 1.95 | 2.7745(11) | 165.7 |
| O9 | H9 | O11^6^ | 0.84 | 1.84 | 2.6766(11) | 174.3 |
| O10 | H10 | O13 | 0.84 | 1.82 | 2.6399(11) | 166.7 |
| O21 | H21A | O18^4^ | 0.87 | 2.66 | 3.377(5) | 140.2 |
| O22 | H22A | O8^7^ | 0.87 | 2.25 | 2.830(9) | 123.7 |
| O22 | H22A | O9 | 0.87 | 2.51 | 3.206(7) | 138.0 |
| O22 | H22B | O13^8^ | 0.87 | 2.16 | 2.956(7) | 151.2 |
| O22 | H22B | O16^8^ | 0.87 | 2.38 | 2.981(6) | 126.8 |

––––

^1^+x,1+y,+z; ^2^+x,1-y,1/2+z; ^3^3/2-x,3/2-y,1-z; ^4^1-x,+y,1/2-z; ^5^+x,-y,-1/2+z; ^6^+x,-1+y,+z; ^7^+x,-y,1/2+z; ^8^3/2-x,1/2-y,1-z

**Table 8**: Atomic Occupancies for all atoms that are not fully occupied in **MAB8**.

| **Atom** | **Occupancy** |
| --- | --- |
| O21 | 0.264(3) |
| H21A | 0.264(3) |
| H21B | 0.264(3) |
| O22 | 0.236(3) |
| H22A | 0.236(3) |
| H22B | 0.236(3) |
