# Supplementary material for: Pentaborate(1-) Salts and a Tetraborate(2-) Salt Derived from C2- or C3-Linked Bis(alkylammonium) Dications: Synthesis, Characterization, and Structural (XRD) Studies
Source: Molecules. 2019 Dec 23;25(1):53. doi: 10.3390/molecules25010053 (PMC6982793; doi:10.3390/molecules25010053)
Supplement: Supplementary file 1 [file molecules-25-00053-s001.zip › MAB9.docx]

**MAB9**


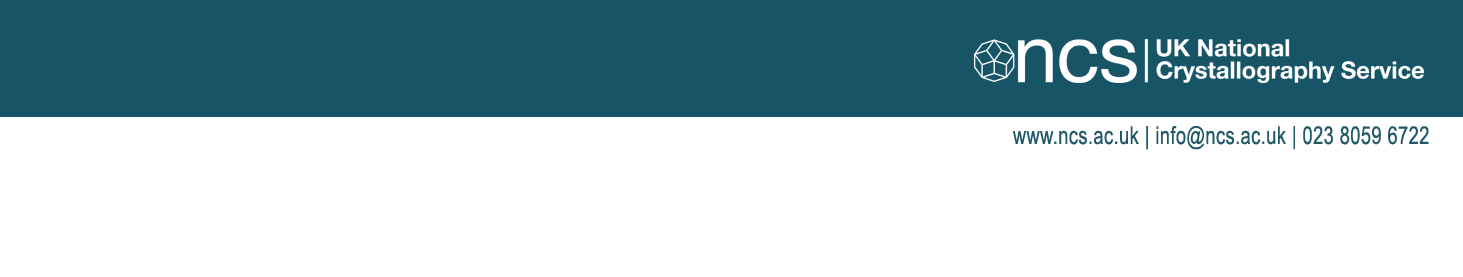


Submitted by: **None**

None

Solved by: **None**

Sample ID: **MAB9**

***R_1_*=3.03%**

Crystal Data and Experimental


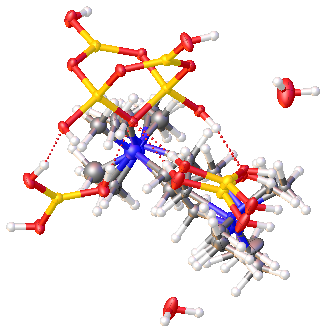


**Experimental.** Single colourless plate crystals of **MAB9** recrystallised from water. A suitable crystal with dimensions 0.130 × 0.080 × 0.025 mm^3^ was selected and mounted on a Rigaku FRE+ equipped with VHF Varimax confocal mirrors and an AFC12 goniometer and HyPix 6000 detector diffractometer. The crystal was kept at a steady *T* = 100(2) K during data collection. The structure was solved with the **ShelXT** 2018/2 (Sheldrick, 2018) solution program using dual methods and by using **Olex2** (Dolomanov et al., 2009) as the graphical interface. The model was refined with **ShelXL** 2018/3 (Sheldrick, 2015) using full matrix least squares minimisation on ***F*^2^**.

**Crystal Data.** C_8_H_36_B_6_N_2_O_17_, *M_r_* = 497.25, monoclinic, *P*2_1_ (No. 4), a = 9.0242(2) Å, b = 12.0350(3) Å, c = 11.1688(4) Å, *β* = 109.811(3)^°^, *α* = *γ* = 90^°^, *V* = 1141.21(6) Å^3^, *T* = 100(2) K, *Z* = 2, *Z'* = 1, *μ*(Mo K*_α_*) = 0.131, 24237 reflections measured, 5229 unique (*R_int_* = 0.0265) which were used in all calculations. The final *wR_2_* was 0.0802 (all data) and *R_1_* was 0.0303 (I > 2(I)).

| **Compound** | **MAB9** |
| --- | --- |
|  |  |
| Formula | C_8_H_36_B_6_N_2_O_17_ |
| *D_calc._*/ g cm^-3^ | 1.447 |
| *μ*/mm^-1^ | 0.131 |
| Formula Weight | 497.25 |
| Colour | colourless |
| Shape | plate |
| Size/mm^3^ | 0.130×0.080×0.025 |
| *T*/K | 100(2) |
| Crystal System | monoclinic |
| Flack Parameter | -0.2(2) |
| Space Group | *P*2_1_ |
| *a*/Å | 9.0242(2) |
| *b*/Å | 12.0350(3) |
| *c*/Å | 11.1688(4) |
| *α*/^°^ | 90 |
| *β*/^°^ | 109.811(3) |
| *γ*/^°^ | 90 |
| V/Å^3^ | 1141.21(6) |
| *Z* | 2 |
| *Z'* | 1 |
| Wavelength/Å | 0.71075 |
| Radiation type | Mo K*_α_* |
| *Θ_min_*/^°^ | 1.938 |
| *Θ_max_*/^°^ | 27.480 |
| Measured Refl's. | 24237 |
| Ind't Refl's | 5229 |
| Refl's with I > 2(I) | 4895 |
| *R_int_* | 0.0265 |
| Parameters | 449 |
| Restraints | 974 |
| Largest Peak | 0.201 |
| Deepest Hole | -0.386 |
| GooF | 1.059 |
| *wR_2_* (all data) | 0.0802 |
| *wR_2_* | 0.0785 |
| *R_1_* (all data) | 0.0337 |
| *R_1_* | 0.0303 |

**Table 1**: Fractional Atomic Coordinates (×10^4^) and Equivalent Isotropic Displacement Parameters (Å^2^×10^3^) for **MAB9**. *U_eq_* is defined as 1/3 of the trace of the orthogonalised *U_ij_*.

| **Atom** | **x** | **y** | **z** | ***U_eq_*** |
| --- | --- | --- | --- | --- |
| O1 | 1822.0(14) | 7556.1(11) | 3043.7(12) | 11.6(3) |
| O2 | 3025.0(14) | 5822.0(11) | 3954.5(12) | 13.1(3) |
| O3 | 753.9(15) | 5814.4(12) | 2067.7(13) | 15.5(3) |
| O4 | 2211.0(15) | 7014.0(12) | 1106.0(13) | 14.5(3) |
| O5 | 4418.0(14) | 7144.0(11) | 3041.6(12) | 12.9(3) |
| O6 | 4023.4(15) | 7489.6(11) | 5032.8(12) | 13.9(3) |
| O7 | -389.5(14) | 7488.6(11) | 1068.0(12) | 13.8(3) |
| O8 | 1315.3(16) | 4250.3(13) | 3372.5(14) | 20.1(3) |
| O9 | 4691.7(15) | 7378.8(15) | 1006.6(13) | 23.2(3) |
| O11 | -2249.5(15) | 7654.1(13) | 2401.7(14) | 19.8(3) |
| O12 | -2619.2(16) | 8046.8(14) | 4350.5(14) | 20.7(3) |
| O13 | -88.3(16) | 8165.8(14) | 4269.4(14) | 22.6(3) |
| O21 | 2414(7) | 9667(3) | 2654(5) | 24.3(10) |
| O22 | 4536(17) | 9687(12) | 4617(9) | 20(2) |
| O23 | 3847(8) | 11293(3) | 3262(4) | 26.9(11) |
| O32 | 195(2) | 9459.8(14) | 10086.8(16) | 32.3(4) |
| B1 | 3315(2) | 7022.5(17) | 3777(2) | 11.6(4) |
| B2 | 1105(2) | 6981.0(17) | 1817(2) | 12.2(4) |
| B3 | 1712(2) | 5305.6(19) | 3138(2) | 13.8(4) |
| B4 | 3768(2) | 7190.1(18) | 1742(2) | 13.7(4) |
| B11 | -1636(2) | 7949.7(18) | 3651(2) | 15.0(4) |
| B21 | 3647(7) | 10206(4) | 3534(6) | 18.2(10) |
| O31 | 6875(2) | 6604(2) | 9340(2) | 50.6(6) |
| N1 | 1668(9) | 6363(6) | 7726(8) | 16.9(15) |
| N2 | 3381(6) | 9267(4) | 7646(5) | 13.8(9) |
| C1 | 2930(5) | 7244(4) | 8044(4) | 20.2(9) |
| C2 | 2423(5) | 8235(4) | 7160(4) | 17.3(9) |
| C3 | 1178(10) | 6017(6) | 6357(6) | 24.7(15) |
| C4 | 2337(10) | 5387(8) | 8598(9) | 30(2) |
| C5 | 238(12) | 6771(9) | 8010(11) | 22.1(19) |
| C6 | 5069(8) | 9030(6) | 7865(7) | 22.6(13) |
| C7 | 3535(16) | 9660(11) | 9023(10) | 55(3) |
| C8 | 2793(7) | 10125(7) | 6635(8) | 27.2(14) |
| N1B | 1299(15) | 6486(10) | 7410(12) | 28(4) |
| N2B | 3430(9) | 9318(6) | 8176(8) | 14.8(17) |
| C1B | 1994(8) | 7579(6) | 7138(7) | 19.2(15) |
| C2B | 2371(8) | 8368(6) | 8261(7) | 17.8(15) |
| C3B | 611(13) | 5912(9) | 6168(9) | 25(3) |
| C4B | 2575(13) | 5801(10) | 8321(12) | 30(4) |
| C5B | -30(20) | 6667(19) | 7930(20) | 22(4) |
| C6B | 5043(12) | 8911(9) | 8338(12) | 28(3) |
| C7B | 3518(11) | 10089(9) | 9243(10) | 27(2) |
| C8B | 2751(17) | 9900(16) | 6961(12) | 27(4) |
| N1C | 1420(20) | 6458(15) | 7610(20) | 23(5) |
| N2C | 3905(11) | 9115(7) | 7970(9) | 10(2) |
| C1C | 1922(10) | 7647(8) | 7977(9) | 20.2(19) |
| C2C | 3550(9) | 7886(7) | 7911(8) | 16.0(17) |
| C3C | 1623(19) | 6064(14) | 6381(15) | 28(4) |
| C4C | 2450(20) | 5660(15) | 8560(20) | 15(3) |
| C5C | -207(16) | 6318(14) | 7622(15) | 44(4) |
| C6C | 5544(13) | 9198(10) | 8060(12) | 15(3) |
| C7C | 3133(16) | 9686(14) | 8799(12) | 13(3) |
| C8C | 2875(16) | 9738(13) | 6796(14) | 21(4) |
| B21A | 3900(20) | 10181(15) | 3253(19) | 18.2(10) |
| O21A | 2821(17) | 9585(9) | 2349(15) | 17(3) |
| O22A | 4570(50) | 9690(40) | 4420(30) | 14(3) |
| O23A | 4348(18) | 11175(8) | 2956(16) | 23(3) |

**Table 2**: Anisotropic Displacement Parameters (×10^4^) for **MAB9**. The anisotropic displacement factor exponent takes the form: *-2π^2^[h^2^a*^2^ × U_11_+ ... +2hka* × b* × U_12_]*

| **Atom** | ***U_11_*** | ***U_22_*** | ***U_33_*** | ***U_23_*** | ***U_13_*** | ***U_12_*** |
| --- | --- | --- | --- | --- | --- | --- |
| O1 | 9.0(6) | 11.5(6) | 13.6(6) | -1.0(5) | 2.9(5) | 1.3(5) |
| O2 | 12.3(6) | 11.1(6) | 13.4(6) | 1.1(5) | 1.0(5) | -0.8(5) |
| O3 | 13.0(6) | 12.8(6) | 16.3(7) | 0.4(5) | -0.5(5) | -1.4(5) |
| O4 | 10.3(6) | 18.4(7) | 13.5(6) | -2.3(5) | 2.2(5) | 0.4(5) |
| O5 | 9.2(6) | 13.6(6) | 14.6(6) | 0.8(5) | 2.2(5) | 0.3(5) |
| O6 | 13.1(6) | 10.8(6) | 15.0(7) | -1.6(5) | 1.2(5) | -0.7(5) |
| O7 | 9.7(6) | 15.4(6) | 14.6(7) | 2.3(5) | 1.8(5) | 1.0(5) |
| O8 | 19.9(7) | 16.4(7) | 17.9(7) | 4.3(6) | -1.5(6) | -5.2(6) |
| O9 | 10.8(6) | 43.0(10) | 14.9(7) | 1.1(7) | 3.4(5) | 0.4(6) |
| O11 | 10.6(6) | 31.6(8) | 17.7(7) | -1.7(6) | 5.7(5) | -0.8(5) |
| O12 | 11.9(6) | 30.0(8) | 20.3(7) | -9.7(6) | 5.6(5) | -3.8(6) |
| O13 | 13.4(6) | 33.1(8) | 21.7(7) | -10.8(6) | 6.5(6) | -2.2(6) |
| O21 | 27.6(19) | 14.9(13) | 20.3(16) | 3.9(11) | -5.3(13) | -5.7(12) |
| O22 | 17(2) | 14.8(18) | 18(3) | 0(2) | -7(2) | -5.6(16) |
| O23 | 37(2) | 17.2(11) | 15.2(15) | 1.1(9) | -5.5(13) | -11.1(12) |
| O32 | 36.7(10) | 23.7(9) | 22.2(8) | 9.7(7) | -8.9(7) | -11.4(7) |
| B1 | 9.6(8) | 10.6(9) | 13.5(9) | -0.1(7) | 2.4(7) | 1.1(7) |
| B2 | 9.7(8) | 11.1(9) | 13.8(9) | 0.3(7) | 1.4(7) | 0.3(7) |
| B3 | 12.1(9) | 14.0(9) | 14.1(10) | -0.8(8) | 2.7(8) | -1.0(7) |
| B4 | 12.3(9) | 14.0(9) | 14.2(9) | 0.5(8) | 3.7(7) | 2.1(7) |
| B11 | 13.6(9) | 13.3(10) | 17.6(10) | -1.6(8) | 4.6(8) | 1.0(8) |
| B21 | 21(2) | 15.4(11) | 15(2) | -0.6(14) | 2.2(14) | -3.0(12) |
| O31 | 38.4(10) | 79.1(16) | 32.6(11) | -6.1(11) | 9.6(8) | -20.0(10) |
| N1 | 18(3) | 10(2) | 20(3) | -6.5(18) | 3(2) | -10(2) |
| N2 | 7.8(19) | 16.1(19) | 14(2) | -5.8(17) | -0.6(18) | -2.0(15) |
| C1 | 15.7(19) | 19(2) | 23(2) | -1.5(16) | 2.7(16) | -6.2(16) |
| C2 | 18.2(19) | 14.2(18) | 18(2) | -3.6(15) | 3.8(15) | -4.7(15) |
| C3 | 13(4) | 41(4) | 19(3) | -4(2) | 4(3) | -9(3) |
| C4 | 40(4) | 20(3) | 31(3) | 7(3) | 12(2) | 2(3) |
| C5 | 21(3) | 19(3) | 26(4) | 3(2) | 8(3) | -6(3) |
| C6 | 12(2) | 25(3) | 23(4) | -9(2) | -3(2) | -5(2) |
| C7 | 93(9) | 43(5) | 50(5) | -22(4) | 50(6) | -10(5) |
| C8 | 24(3) | 16(3) | 39(4) | 4(3) | 8(3) | 1(2) |
| B21A | 21(2) | 15.4(11) | 15(2) | -0.6(14) | 2.2(14) | -3.0(12) |
| O21A | 14(4) | 9(3) | 22(5) | 2(3) | -3(3) | 2(3) |
| O22A | 16(6) | 9(5) | 17(5) | -2(5) | 5(5) | -3(4) |
| O23A | 23(5) | 15(3) | 23(5) | 2(3) | -4(4) | -5(3) |

**Table 3**: Bond Lengths in Å for **MAB9**.

| **Atom** | **Atom** | **Length/Å** |
| --- | --- | --- |
| O1 | B1 | 1.466(2) |
| O1 | B2 | 1.475(2) |
| O2 | B1 | 1.493(2) |
| O2 | B3 | 1.375(3) |
| O3 | B2 | 1.487(2) |
| O3 | B3 | 1.360(3) |
| O4 | B2 | 1.471(2) |
| O4 | B4 | 1.359(2) |
| O5 | B1 | 1.497(2) |
| O5 | B4 | 1.370(3) |
| O6 | B1 | 1.444(2) |
| O7 | B2 | 1.458(2) |
| O8 | B3 | 1.369(3) |
| O9 | B4 | 1.373(2) |
| O11 | B11 | 1.362(3) |
| O12 | B11 | 1.372(2) |
| O13 | B11 | 1.357(3) |
| O21 | B21 | 1.373(5) |
| O22 | B21 | 1.356(9) |
| O23 | B21 | 1.368(5) |
| N1 | C1 | 1.507(7) |
| N1 | C3 | 1.499(8) |
| N1 | C4 | 1.514(10) |
| N1 | C5 | 1.512(11) |
| N2 | C2 | 1.506(6) |
| N2 | C6 | 1.486(8) |
| N2 | C7 | 1.569(9) |
| N2 | C8 | 1.489(9) |
| C1 | C2 | 1.517(6) |
| N1B | C1B | 1.531(13) |
| N1B | C3B | 1.484(13) |
| N1B | C4B | 1.499(13) |
| N1B | C5B | 1.517(15) |
| N2B | C2B | 1.513(9) |
| N2B | C6B | 1.487(11) |
| N2B | C7B | 1.492(12) |
| N2B | C8B | 1.464(13) |
| C1B | C2B | 1.518(10) |
| N1C | C1C | 1.514(17) |
| N1C | C3C | 1.517(18) |
| N1C | C4C | 1.501(18) |
| N1C | C5C | 1.488(17) |
| N2C | C2C | 1.510(10) |
| N2C | C6C | 1.452(12) |
| N2C | C7C | 1.500(14) |
| N2C | C8C | 1.523(14) |
| C1C | C2C | 1.523(10) |
| B21A | O21A | 1.35(2) |
| B21A | O22A | 1.37(3) |
| B21A | O23A | 1.338(19) |

**Table 4**: Bond Angles in ^°^ for **MAB9**.

| **Atom** | **Atom** | **Atom** | **Angle/^°^** |
| --- | --- | --- | --- |
| B1 | O1 | B2 | 110.13(14) |
| B3 | O2 | B1 | 119.97(15) |
| B3 | O3 | B2 | 118.56(15) |
| B4 | O4 | B2 | 119.50(15) |
| B4 | O5 | B1 | 117.36(14) |
| O1 | B1 | O2 | 109.19(14) |
| O1 | B1 | O5 | 107.86(15) |
| O2 | B1 | O5 | 110.27(14) |
| O6 | B1 | O1 | 113.11(15) |
| O6 | B1 | O2 | 106.71(15) |
| O6 | B1 | O5 | 109.70(15) |
| O1 | B2 | O3 | 108.82(15) |
| O4 | B2 | O1 | 108.93(14) |
| O4 | B2 | O3 | 110.74(15) |
| O7 | B2 | O1 | 111.43(15) |
| O7 | B2 | O3 | 106.79(14) |
| O7 | B2 | O4 | 110.11(15) |
| O3 | B3 | O2 | 121.85(18) |
| O3 | B3 | O8 | 117.16(17) |
| O8 | B3 | O2 | 120.99(18) |
| O4 | B4 | O5 | 122.88(17) |
| O4 | B4 | O9 | 116.32(17) |
| O5 | B4 | O9 | 120.76(17) |
| O11 | B11 | O12 | 119.23(17) |
| O13 | B11 | O11 | 124.25(17) |
| O13 | B11 | O12 | 116.52(18) |
| O22 | B21 | O21 | 120.7(7) |
| O22 | B21 | O23 | 123.8(7) |
| O23 | B21 | O21 | 115.4(4) |
| C1 | N1 | C4 | 106.8(6) |
| C1 | N1 | C5 | 110.6(6) |
| C3 | N1 | C1 | 112.0(6) |
| C3 | N1 | C4 | 110.9(6) |
| C3 | N1 | C5 | 109.0(8) |
| C5 | N1 | C4 | 107.4(7) |
| C2 | N2 | C7 | 116.8(6) |
| C6 | N2 | C2 | 109.4(4) |
| C6 | N2 | C7 | 98.7(6) |
| C6 | N2 | C8 | 109.9(5) |
| C8 | N2 | C2 | 106.6(5) |
| C8 | N2 | C7 | 115.2(7) |
| N1 | C1 | C2 | 111.3(5) |
| N2 | C2 | C1 | 113.9(4) |
| C3B | N1B | C1B | 106.5(9) |
| C3B | N1B | C4B | 111.1(10) |
| C3B | N1B | C5B | 106.9(12) |
| C4B | N1B | C1B | 109.6(9) |
| C4B | N1B | C5B | 110.1(12) |
| C5B | N1B | C1B | 112.6(12) |
| C6B | N2B | C2B | 110.9(7) |
| C6B | N2B | C7B | 108.8(8) |
| C7B | N2B | C2B | 106.4(7) |
| C8B | N2B | C2B | 110.2(9) |
| C8B | N2B | C6B | 110.8(9) |
| C8B | N2B | C7B | 109.6(10) |
| C2B | C1B | N1B | 111.5(7) |
| N2B | C2B | C1B | 113.0(6) |
| C1C | N1C | C3C | 115.3(14) |
| C4C | N1C | C1C | 110.9(14) |
| C4C | N1C | C3C | 102.0(15) |
| C5C | N1C | C1C | 107.9(13) |
| C5C | N1C | C3C | 113.3(14) |
| C5C | N1C | C4C | 107.2(15) |
| C2C | N2C | C8C | 112.7(9) |
| C6C | N2C | C2C | 105.5(8) |
| C6C | N2C | C7C | 126.9(10) |
| C6C | N2C | C8C | 109.6(9) |
| C7C | N2C | C2C | 109.9(9) |
| C7C | N2C | C8C | 91.7(9) |
| N1C | C1C | C2C | 111.6(9) |
| N2C | C2C | C1C | 112.1(7) |
| O21A | B21A | O22A | 117(2) |
| O23A | B21A | O21A | 119.5(14) |
| O23A | B21A | O22A | 123(2) |

**Table 5**: Torsion Angles in ^°^ for **MAB9**.

| **Atom** | **Atom** | **Atom** | **Atom** | **Angle/^°^** |
| --- | --- | --- | --- | --- |
| B1 | O1 | B2 | O3 | 62.12(17) |
| B1 | O1 | B2 | O4 | -58.71(18) |
| B1 | O1 | B2 | O7 | 179.61(14) |
| B1 | O2 | B3 | O3 | 8.3(3) |
| B1 | O2 | B3 | O8 | -171.69(17) |
| B1 | O5 | B4 | O4 | -7.1(3) |
| B1 | O5 | B4 | O9 | 175.42(18) |
| B2 | O1 | B1 | O2 | -56.83(18) |
| B2 | O1 | B1 | O5 | 63.01(18) |
| B2 | O1 | B1 | O6 | -175.48(14) |
| B2 | O3 | B3 | O2 | -3.0(3) |
| B2 | O3 | B3 | O8 | 177.02(16) |
| B2 | O4 | B4 | O5 | 12.0(3) |
| B2 | O4 | B4 | O9 | -170.44(17) |
| B3 | O2 | B1 | O1 | 21.9(2) |
| B3 | O2 | B1 | O5 | -96.42(18) |
| B3 | O2 | B1 | O6 | 144.50(16) |
| B3 | O3 | B2 | O1 | -31.7(2) |
| B3 | O3 | B2 | O4 | 88.0(2) |
| B3 | O3 | B2 | O7 | -152.10(15) |
| B4 | O4 | B2 | O1 | 20.9(2) |
| B4 | O4 | B2 | O3 | -98.7(2) |
| B4 | O4 | B2 | O7 | 143.42(17) |
| B4 | O5 | B1 | O1 | -29.9(2) |
| B4 | O5 | B1 | O2 | 89.26(19) |
| B4 | O5 | B1 | O6 | -153.49(16) |
| N1 | C1 | C2 | N2 | 162.7(5) |
| C3 | N1 | C1 | C2 | 54.6(7) |
| C4 | N1 | C1 | C2 | 176.1(5) |
| C5 | N1 | C1 | C2 | -67.2(8) |
| C6 | N2 | C2 | C1 | 57.9(6) |
| C7 | N2 | C2 | C1 | -53.0(8) |
| C8 | N2 | C2 | C1 | 176.6(5) |
| N1B | C1B | C2B | N2B | 165.1(7) |
| C3B | N1B | C1B | C2B | 165.9(8) |
| C4B | N1B | C1B | C2B | -73.8(10) |
| C5B | N1B | C1B | C2B | 49.1(14) |
| C6B | N2B | C2B | C1B | -69.0(9) |
| C7B | N2B | C2B | C1B | 172.8(7) |
| C8B | N2B | C2B | C1B | 54.1(11) |
| N1C | C1C | C2C | N2C | 166.5(11) |
| C3C | N1C | C1C | C2C | -48.9(17) |
| C4C | N1C | C1C | C2C | 66.3(16) |
| C5C | N1C | C1C | C2C | -176.6(11) |
| C6C | N2C | C2C | C1C | 173.0(8) |
| C7C | N2C | C2C | C1C | 33.2(11) |
| C8C | N2C | C2C | C1C | -67.5(11) |

**Table 6**: Hydrogen Fractional Atomic Coordinates (×10^4^) and Equivalent Isotropic Displacement Parameters (Å^2^×10^3^) for **MAB9**. *U_eq_* is defined as 1/3 of the trace of the orthogonalised *U_ij_*.

| **Atom** | **x** | **y** | **z** | ***U_eq_*** |
| --- | --- | --- | --- | --- |
| H6 | 4291.72 | 8132.25 | 4972.51 | 21 |
| H7 | -221.05 | 8072.07 | 757.32 | 21 |
| H8 | 1909.78 | 4044.05 | 4069.54 | 30 |
| H9 | 5614.57 | 7437.34 | 1466.62 | 35 |
| H11 | -1545.53 | 7638.68 | 2092.12 | 30 |
| H12 | -3496.31 | 7813.59 | 3933.22 | 31 |
| H13 | 423.35 | 7987.57 | 3817.14 | 34 |
| H21 | 2273.88 | 9064.43 | 2942.18 | 37 |
| H22 | 5225.1 | 10110.24 | 5042.65 | 30 |
| H23 | 4605.49 | 11553.73 | 3827.86 | 40 |
| H32A | -150.85 | 9907.67 | 9464.51 | 49 |
| H32B | 1009.65 | 9734.99 | 10637.61 | 49 |
| H31A | 7802.16 | 6814 | 9769.84 | 76 |
| H31B | 6788.3 | 6549.48 | 8560.44 | 76 |
| H1A | 3888 | 6932.1 | 7973.53 | 24 |
| H1B | 3153.95 | 7485.8 | 8915.9 | 24 |
| H2A | 2502.86 | 8040.79 | 6340.99 | 21 |
| H2B | 1325.75 | 8395.33 | 7028.47 | 21 |
| H3A | 647.77 | 6622.97 | 5825.21 | 37 |
| H3B | 478.93 | 5391.62 | 6218.47 | 37 |
| H3C | 2092.5 | 5814.29 | 6152.18 | 37 |
| H4A | 3230.03 | 5092.77 | 8421.61 | 45 |
| H4B | 1546.79 | 4821.06 | 8457.89 | 45 |
| H4C | 2658.07 | 5629.41 | 9467.78 | 45 |
| H5A | 569.21 | 7145.1 | 8816.7 | 33 |
| H5B | -417.25 | 6150.26 | 8037.97 | 33 |
| H5C | -345.27 | 7276.83 | 7355.18 | 33 |
| H6A | 5483.2 | 8564.13 | 8601.22 | 34 |
| H6B | 5647.1 | 9714.89 | 8000.36 | 34 |
| H6C | 5165.42 | 8657.43 | 7135.18 | 34 |
| H7A | 2504.78 | 9743.31 | 9083.15 | 83 |
| H7B | 4078.01 | 10359.11 | 9198.32 | 83 |
| H7C | 4117.78 | 9116.98 | 9630.8 | 83 |
| H8A | 2839.79 | 9839.79 | 5845.82 | 41 |
| H8B | 3436.16 | 10778.52 | 6870.19 | 41 |
| H8C | 1722.76 | 10309.52 | 6536.39 | 41 |
| H1BA | 2947.62 | 7424.12 | 6950.99 | 23 |
| H1BB | 1247.71 | 7929.1 | 6395.15 | 23 |
| H2BA | 1395.68 | 8669.45 | 8308.58 | 21 |
| H2BB | 2880.15 | 7956.02 | 9038.89 | 21 |
| H3BA | -200.1 | 6368.21 | 5603.15 | 37 |
| H3BB | 167.92 | 5215.12 | 6293.86 | 37 |
| H3BC | 1418.73 | 5783.14 | 5804.85 | 37 |
| H4BA | 3419.89 | 5703.95 | 7989.73 | 45 |
| H4BB | 2160.19 | 5087.57 | 8427.12 | 45 |
| H4BC | 2963.93 | 6172.09 | 9128.02 | 45 |
| H5BA | 401.2 | 6827.25 | 8825.61 | 34 |
| H5BB | -664.99 | 6007.58 | 7803.46 | 34 |
| H5BC | -669.18 | 7279.11 | 7497.59 | 34 |
| H6BA | 5432.39 | 8481.8 | 9106.29 | 41 |
| H6BB | 5726.59 | 9533.52 | 8389.49 | 41 |
| H6BC | 5010.15 | 8455.23 | 7624.53 | 41 |
| H7BA | 2503.08 | 10417.59 | 9099.34 | 41 |
| H7BB | 4274.35 | 10662.18 | 9288.15 | 41 |
| H7BC | 3833.27 | 9682.44 | 10029.44 | 41 |
| H8BA | 2887.44 | 9457.95 | 6290.77 | 41 |
| H8BB | 3271.63 | 10601.52 | 7001.29 | 41 |
| H8BC | 1647.88 | 10022.27 | 6795.43 | 41 |
| H1CA | 1932.31 | 7782.28 | 8836.06 | 24 |
| H1CB | 1162.47 | 8148.13 | 7410.37 | 24 |
| H2CA | 4337.61 | 7514.91 | 8611.97 | 19 |
| H2CB | 3614.19 | 7585.04 | 7123.98 | 19 |
| H3CA | 1170.46 | 6599.55 | 5721.35 | 43 |
| H3CB | 1101.63 | 5362.35 | 6138.52 | 43 |
| H3CC | 2724.04 | 5980.36 | 6507.73 | 43 |
| H4CA | 3491.13 | 5663.61 | 8509.83 | 22 |
| H4CB | 2016.31 | 4925.21 | 8389.13 | 22 |
| H4CC | 2505.26 | 5879.01 | 9403.75 | 22 |
| H5CA | -223.76 | 6463.28 | 8462.79 | 66 |
| H5CB | -555.09 | 5571.45 | 7380.5 | 66 |
| H5CC | -896.59 | 6829.58 | 7032.45 | 66 |
| H6CA | 6183.49 | 8761.14 | 8764.62 | 22 |
| H6CB | 5873.7 | 9960.81 | 8186.25 | 22 |
| H6CC | 5660.11 | 8928.43 | 7287.37 | 22 |
| H7CA | 2009.86 | 9630.91 | 8415.31 | 20 |
| H7CB | 3435.57 | 10454.83 | 8891.64 | 20 |
| H7CC | 3461.07 | 9338.28 | 9621.27 | 20 |
| H8CA | 2760.61 | 9302.55 | 6049.91 | 31 |
| H8CB | 3358.79 | 10436.35 | 6734.06 | 31 |
| H8CC | 1857.14 | 9868.52 | 6863.89 | 31 |
| H21A | 2575.69 | 9035.52 | 2675 | 26 |
| H22A | 5406.4 | 9996.76 | 4806.96 | 21 |
| H23A | 5109.86 | 11398.42 | 3546.6 | 35 |

**Table 7**: Hydrogen Bond information for **MAB9**.

| **D** | **H** | **A** | **d(D-H)/Å** | **d(H-A)/Å** | **d(D-A)/Å** | **D-H-A/deg** |
| --- | --- | --- | --- | --- | --- | --- |
| O6 | H6 | O22 | 0.82 | 1.94 | 2.751(14) | 169.5 |
| O6 | H6 | O22A | 0.82 | 2.02 | 2.82(4) | 166.8 |
| O7 | H7 | O32^1^ | 0.82 | 1.92 | 2.738(2) | 178.0 |
| O8 | H8 | O12^2^ | 0.82 | 2.05 | 2.815(2) | 155.2 |
| O9 | H9 | O11^3^ | 0.82 | 1.87 | 2.6909(19) | 174.7 |
| O11 | H11 | O7 | 0.82 | 1.80 | 2.6020(18) | 165.7 |
| O12 | H12 | O5^4^ | 0.82 | 1.98 | 2.7958(18) | 174.9 |
| O13 | H13 | O1 | 0.82 | 1.83 | 2.6427(19) | 170.9 |
| O21 | H21 | O1 | 0.82 | 1.87 | 2.662(4) | 161.2 |
| O22 | H22 | O2^5^ | 0.82 | 1.81 | 2.622(14) | 169.5 |
| O23 | H23 | O6^5^ | 0.82 | 1.83 | 2.629(3) | 164.1 |
| O32 | H32A | O3^6^ | 0.85 | 1.95 | 2.790(2) | 171.8 |
| O32 | H32B | O21^7^ | 0.85 | 2.18 | 2.899(4) | 142.3 |
| O32 | H32B | O21A^7^ | 0.85 | 2.06 | 2.822(11) | 149.0 |
| O31 | H31A | O7^8^ | 0.85 | 1.95 | 2.779(2) | 163.2 |
| O31 | H31B | O23^9^ | 0.85 | 1.94 | 2.780(5) | 167.8 |
| O31 | H31B | O23A^9^ | 0.85 | 1.72 | 2.479(14) | 148.0 |
| O21A | H21A | O1 | 0.82 | 2.00 | 2.801(14) | 166.3 |
| O22A | H22A | O2^5^ | 0.82 | 1.89 | 2.68(4) | 161.2 |
| O23A | H23A | O6^5^ | 0.82 | 2.00 | 2.733(13) | 147.9 |

––––

^1^+x,+y,-1+z; ^2^-x,-1/2+y,1-z; ^3^1+x,+y,+z; ^4^-1+x,+y,+z; ^5^1-x,1/2+y,1-z; ^6^-x,1/2+y,1-z; ^7^+x,+y,1+z; ^8^1+x,+y,1+z; ^9^1-x,-1/2+y,1-z

**Table 8**: Atomic Occupancies for all atoms that are not fully occupied in **MAB9**.

| **Atom** | **Occupancy** |
| --- | --- |
| O21 | 0.76(2) |
| H21 | 0.76(2) |
| O22 | 0.76(2) |
| H22 | 0.76(2) |
| O23 | 0.76(2) |
| H23 | 0.76(2) |
| B21 | 0.76(2) |
| N1 | 0.474(4) |
| N2 | 0.474(4) |
| C1 | 0.474(4) |
| H1A | 0.474(4) |
| H1B | 0.474(4) |
| C2 | 0.474(4) |
| H2A | 0.474(4) |
| H2B | 0.474(4) |
| C3 | 0.474(4) |
| H3A | 0.474(4) |
| H3B | 0.474(4) |
| H3C | 0.474(4) |
| C4 | 0.474(4) |
| H4A | 0.474(4) |
| H4B | 0.474(4) |
| H4C | 0.474(4) |
| C5 | 0.474(4) |
| H5A | 0.474(4) |
| H5B | 0.474(4) |
| H5C | 0.474(4) |
| C6 | 0.474(4) |
| H6A | 0.474(4) |
| H6B | 0.474(4) |
| H6C | 0.474(4) |
| C7 | 0.474(4) |
| H7A | 0.474(4) |
| H7B | 0.474(4) |
| H7C | 0.474(4) |
| C8 | 0.474(4) |
| H8A | 0.474(4) |
| H8B | 0.474(4) |
| H8C | 0.474(4) |
| N1B | 0.287(4) |
| N2B | 0.287(4) |
| C1B | 0.287(4) |
| H1BA | 0.287(4) |
| H1BB | 0.287(4) |
| C2B | 0.287(4) |
| H2BA | 0.287(4) |
| H2BB | 0.287(4) |
| C3B | 0.287(4) |
| H3BA | 0.287(4) |
| H3BB | 0.287(4) |
| H3BC | 0.287(4) |
| C4B | 0.287(4) |
| H4BA | 0.287(4) |
| H4BB | 0.287(4) |
| H4BC | 0.287(4) |
| C5B | 0.287(4) |
| H5BA | 0.287(4) |
| H5BB | 0.287(4) |
| H5BC | 0.287(4) |
| C6B | 0.287(4) |
| H6BA | 0.287(4) |
| H6BB | 0.287(4) |
| H6BC | 0.287(4) |
| C7B | 0.287(4) |
| H7BA | 0.287(4) |
| H7BB | 0.287(4) |
| H7BC | 0.287(4) |
| C8B | 0.287(4) |
| H8BA | 0.287(4) |
| H8BB | 0.287(4) |
| H8BC | 0.287(4) |
| N1C | 0.239(4) |
| N2C | 0.239(4) |
| C1C | 0.239(4) |
| H1CA | 0.239(4) |
| H1CB | 0.239(4) |
| C2C | 0.239(4) |
| H2CA | 0.239(4) |
| H2CB | 0.239(4) |
| C3C | 0.239(4) |
| H3CA | 0.239(4) |
| H3CB | 0.239(4) |
| H3CC | 0.239(4) |
| C4C | 0.239(4) |
| H4CA | 0.239(4) |
| H4CB | 0.239(4) |
| H4CC | 0.239(4) |
| C5C | 0.239(4) |
| H5CA | 0.239(4) |
| H5CB | 0.239(4) |
| H5CC | 0.239(4) |
| C6C | 0.239(4) |
| H6CA | 0.239(4) |
| H6CB | 0.239(4) |
| H6CC | 0.239(4) |
| C7C | 0.239(4) |
| H7CA | 0.239(4) |
| H7CB | 0.239(4) |
| H7CC | 0.239(4) |
| C8C | 0.239(4) |
| H8CA | 0.239(4) |
| H8CB | 0.239(4) |
| H8CC | 0.239(4) |
| B21A | 0.24(2) |
| O21A | 0.24(2) |
| H21A | 0.24(2) |
| O22A | 0.24(2) |
| H22A | 0.24(2) |
| O23A | 0.24(2) |
| H23A | 0.24(2) |
