# Supplementary material for: Pentaborate(1-) Salts and a Tetraborate(2-) Salt Derived from C2- or C3-Linked Bis(alkylammonium) Dications: Synthesis, Characterization, and Structural (XRD) Studies
Source: Molecules. 2019 Dec 23;25(1):53. doi: 10.3390/molecules25010053 (PMC6982793; doi:10.3390/molecules25010053)

checkCIF/PLATON report

Structure factors have been supplied for datablock(s) MAB9

THIS REPORT IS FOR GUIDANCE ONLY. IF USED AS PART OF A REVIEW PROCEDURE FOR PUBLICATION, IT SHOULD NOT REPLACE THE EXPERTISE OF AN EXPERIENCED CRYSTALLOGRAPHIC REFEREE.

No syntax errors found. CIF dictionary Interpreting this report

Datablock: MAB9

```
Bond precision:  O- B = 0.0023 A         Wavelength=0.71075

Cell:            a=9.0242(2)    b=12.0350(3)        c=11.1688(4)
                 alpha=90       beta=109.811(3)     gamma=90
Temperature:     100 K
```

	Calculated	Reported
Volume	1141.21(6)	1141.21(6)
Space group	P 21	P 1 21 1
Hall group	P 2yb	P 2yb
Moiety formula	B4 H4 O9, C8 H22 N2, 2(B H3 O3), 2(H2 O)	B4 H4 O9, 2(B H3 O3), 2(H2 O), 1(C8 H22 N2)
Sum formula	C8 H36 B6 N2 O17	C8 H36 B6 N2 O17
Mr	497.25	497.25
Dx,g cm-3	1.447	1.447
Z	2	2
Mu (mm-1)	0.131	0.131
F000	528.0	528.0
F000’	528.38	
h,k,lmax	11,15,14	11,15,14
Nref	5256[2754]	5229
Tmin,Tmax	0.988,0.997	0.772,1.000
Tmin’	0.983	

```
Correction method= # Reported T Limits: Tmin=0.772 Tmax=1.000
AbsCorr = MULTI-SCAN

Data completeness= 1.90/0.99      Theta(max)= 27.480

R(reflections)= 0.0303( 4895)     wR2(reflections)= 0.0802( 5229)

S = 1.059                  Npar= 449
```

The following ALERTS were generated. Each ALERT has the format
test-name_ALERT_alert-type_alert-level.
Click on the hyperlinks for more details of the test.

Alert level C

```
PLAT089_ALERT_3_C Poor Data / Parameter Ratio (Zmax < 18) ........       6.13 Note
```

Alert level G

```
PLAT002_ALERT_2_G Number of Distance or Angle Restraints on AtSite         31 Note
PLAT003_ALERT_2_G Number of Uiso or Uij Restrained non-H Atoms ...         55 Report
PLAT007_ALERT_5_G Number of Unrefined Donor-H Atoms ..............         17 Report
PLAT042_ALERT_1_G Calc. and Reported MoietyFormula Strings  Differ     Please Check
PLAT171_ALERT_4_G The CIF-Embedded .res File Contains EADP Records          1 Report
PLAT172_ALERT_4_G The CIF-Embedded .res File Contains DFIX Records          1 Report
PLAT175_ALERT_4_G The CIF-Embedded .res File Contains SAME Records          2 Report
PLAT178_ALERT_4_G The CIF-Embedded .res File Contains SIMU Records          1 Report
PLAT187_ALERT_4_G The CIF-Embedded .res File Contains RIGU Records          1 Report
PLAT302_ALERT_4_G Anion/Solvent/Minor-Residue Disorder (Resd  2  )       100% Note
PLAT302_ALERT_4_G Anion/Solvent/Minor-Residue Disorder (Resd  3  )       100% Note
PLAT302_ALERT_4_G Anion/Solvent/Minor-Residue Disorder (Resd  4  )       100% Note
PLAT302_ALERT_4_G Anion/Solvent/Minor-Residue Disorder (Resd  6  )       100% Note
PLAT302_ALERT_4_G Anion/Solvent/Minor-Residue Disorder (Resd  7  )       100% Note
PLAT304_ALERT_4_G Non-Integer Number of Atoms in ...... Resd  2         15.17 Check
PLAT304_ALERT_4_G Non-Integer Number of Atoms in ...... Resd  3          9.18 Check
PLAT304_ALERT_4_G Non-Integer Number of Atoms in ...... Resd  4          7.65 Check
PLAT304_ALERT_4_G Non-Integer Number of Atoms in ...... Resd  6          5.32 Check
PLAT304_ALERT_4_G Non-Integer Number of Atoms in ...... Resd  7          1.68 Check
PLAT432_ALERT_2_G Short Inter X...Y Contact  O31      ..C7B              2.52 Ang.
                                             1-x,-1/2+y,2-z  =      2_647 Check
PLAT720_ALERT_4_G Number of Unusual/Non-Standard Labels ..........         44 Note
PLAT790_ALERT_4_G Centre of Gravity not Within Unit Cell: Resd.  #          5 Note
              B H3 O3
PLAT811_ALERT_5_G No ADDSYM Analysis: Too Many Excluded Atoms ....          ! Info
PLAT860_ALERT_3_G Number of Least-Squares Restraints .............        974 Note
```

```
   0 ALERT level A = Most likely a serious problem - resolve or explain
   0 ALERT level B = A potentially serious problem, consider carefully
   1 ALERT level C = Check. Ensure it is not caused by an omission or oversight
  24 ALERT level G = General information/check it is not something unexpected

   1 ALERT type 1 CIF construction/syntax error, inconsistent or missing data
   3 ALERT type 2 Indicator that the structure model may be wrong or deficient
   2 ALERT type 3 Indicator that the structure quality may be low
  17 ALERT type 4 Improvement, methodology, query or suggestion
   2 ALERT type 5 Informative message, check
```

It is advisable to attempt to resolve as many as possible of the alerts in all categories. Often the minor alerts point to easily fixed oversights, errors and omissions in your CIF or refinement strategy, so attention to these fine details can be worthwhile. In order to resolve some of the more serious problems it may be necessary to carry out additional measurements or structure refinements. However, the purpose of your study may justify the reported deviations and the more serious of these should normally be commented upon in the discussion or experimental section of a paper or in the "special_details" fields of the CIF. checkCIF was carefully designed to identify outliers and unusual parameters, but every test has its limitations and alerts that are not important in a particular case may appear. Conversely, the absence of alerts does not guarantee there are no aspects of the results needing attention. It is up to the individual to critically assess their own results and, if necessary, seek expert advice.

Publication of your CIF in IUCr journals

A basic structural check has been run on your CIF. These basic checks will be run on all CIFs submitted for publication in IUCr journals (*Acta Crystallographica*, *Journal of Applied Crystallography*, *Journal of Synchrotron Radiation*); however, if you intend to submit to *Acta Crystallographica Section C* or *E* or *IUCrData*, you should make sure that full publication checks are run on the final version of your CIF prior to submission.

Publication of your CIF in other journals

Please refer to the *Notes for Authors* of the relevant journal for any special instructions relating to CIF submission.

PLATON version of 07/08/2019; check.def file version of 30/07/2019

Datablock MAB9 - ellipsoid plot

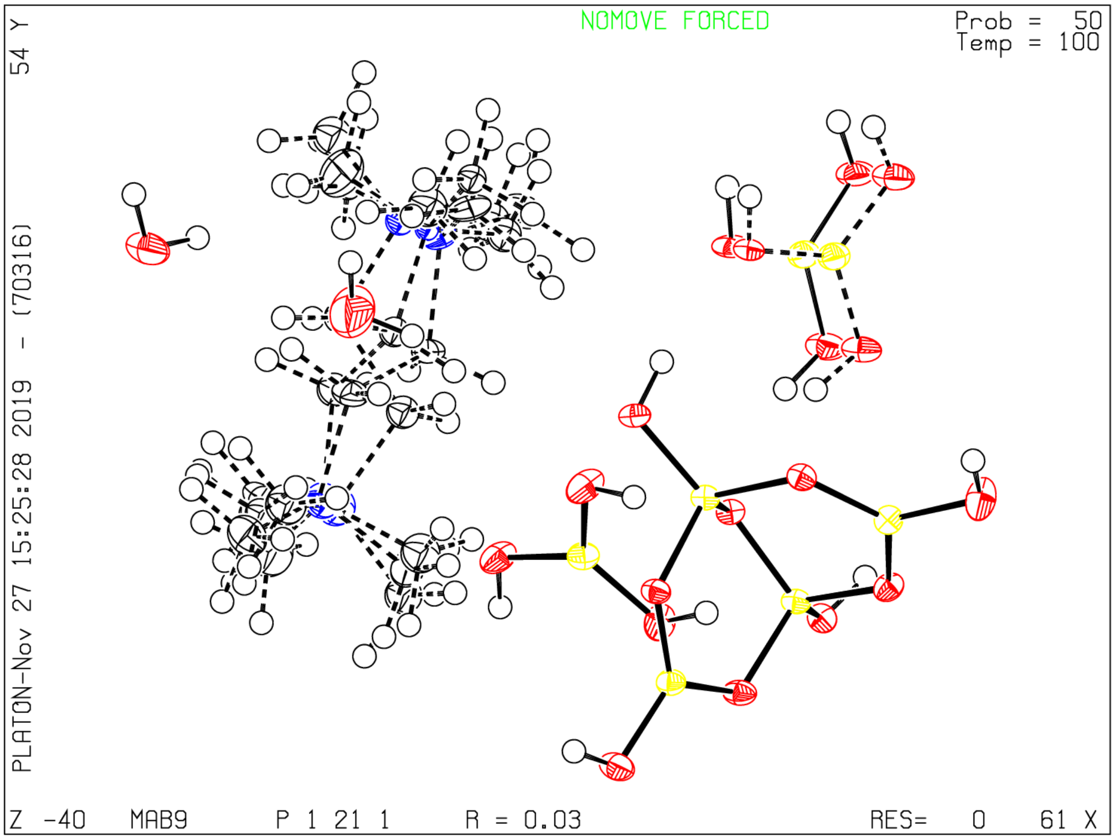

Supplement: Supplementary file 1 [file molecules-25-00053-s001.zip › MAB9_checkcif.pdf]
